# Constructing a cancer stem cell related prognostic model for predicting immune landscape and drug sensitivity in colorectal cancer

**DOI:** 10.3389/fphar.2023.1200017

**Published:** 2023-06-12

**Authors:** Jianfang Chen, Shuang Wu, Yu Peng, Yang Zhao, Yan Dong, Fengwei Ran, Haofei Geng, Kang Zhang, Jianjun Li, Shuo Huang, Zhe Wang

**Affiliations:** Department of Oncology and Southwest Cancer Center, Southwest Hospital, Third Military Medical University (Army Medical University), Chongqing, China

**Keywords:** colorectal cancer, cancer stem cells, single-cell analysis, CSC marker genes, clustering, prognostic model, oxidative stress, drug sensitivity

## Abstract

**Background:** Colorectal cancer (CRC) ranks the second malignancy with high incidence and mortality worldwide. Cancer stem cells (CSCs) function critically in cancer progression and metastasis via the interplay with immune cells in tumor microenvironment. This study aimed to identify important CSC marker genes and parsed the role of these marker genes in CRC.

**Materials and methods:** CRC samples’ single-cell RNA sequencing data and bulk transcriptome data were utilized. Seurat R package annotated CSCs and identified CSC marker genes. Consensus clustering subtyped CRC samples based on CSC marker genes. Immune microenvironment, pathway and oxidative stress analysis was performed using ESTIMATE, MCP-counter analysis and ssGSEA analysis. A prognostic model was established by Lasso and stepAIC. Sensitivity to chemotherapeutic drugs was determined by the biochemical half maximal inhibitory concentration with pRRophetic R package.

**Results:** We identified a total of 29 CSC marker genes related to disease-specific survival (DSS). Two clusters (CSC1 and CSC2) were determined, and CSC2 showed shorter DSS, a larger proportion of late-stage samples, and higher oxidative stress response. Two clusters exhibited differential activation of biological pathways associated with immune response and oncogenic signaling. Drug sensitivity analysis showed that 44 chemotherapy drugs were more sensitive to CSC2 that those in CSC1. We constructed a seven-gene prognostic model (DRD4, DPP7, UCN, INHBA, SFTA2, SYNPO2, and NXPH4) that was effectively to distinguish high-risk and low-risk patients. 14 chemotherapy drugs were more sensitive to high-risk group and 13 chemotherapy drugs were more sensitive to low-risk group. Combination of higher oxidative stress and risk score indicated dismal prognosis.

**Conclusion:** The CSC marker genes we identified may help to further decipher the role of CSCs in CRC development and progression. The seven-gene prognostic model could serve as an indicator for predicting the response to immunotherapy and chemotherapy as well as prognosis of CRC patients.

## Introduction

According to global cancer statistics in 2020, colorectal cancer (CRC) is one of the most contributable malignancies worldwide, resulting in around 9.8% of new cancer cases and 9.2% of new cancer deaths worldwide (Sung et al., 2021). Males have both higher incidence and mortality rates than females, which may result from more frequent smoking in males. The gender disparity also varies greatly by age. For instance, incidence of aging from 55–74 is 40%–50% higher in male population than in females, while the close incidence is shown between men and women in ages beneath 45 years (Murphy et al., 2011). The incidence markedly escalates with the increasing age from 40 years, presented with almost or even over double increase per 10 years (Siegel et al., 2020). With the developing and popularization of screening methods like colonoscopy, the incidence rate of CRC drastically declined from late 20th century to 2017 (Laiyemo et al., 2010; Fedewa et al., 2017). Nevertheless, survival rate for 5 years is still extremely low, about 12% for metastatic CRC patients (Siegel et al., 2019). Further investigation on molecular mechanisms and screening or prognosis predicting methods is needed for facilitating survival of CRC patients.

In recent years, molecular stratification therapy based on tumor biological characteristics has improved the prognosis of patients with advanced colorectal cancer to some extent. For example, anti-D-1 and anti-CTLA-4 monoclonal antibodies for metastatic disease with MSI or high TMB (Hong et al., 2016; Tamura, 2018) and verofinil for colorectal cancer with BRAFV600E. Dienstmann et al. (Dienstmann et al., 2017) pointed out that precision therapy for colorectal cancer will shift from single-gene single-drug to multi-gene-multi-drug as well as multi-molecular multi-drug, i.e., from a clonal perspective to a clone-stromal-immune perspective, which represents the future direction of colorectal cancer treatment.

The substantial proliferation and invasion of cancer cells are tightly linked to cancer stem cells (CSCs). CSCs possess a solid self-renewal ability to expand cancer cell growth and promote tumorigenesis (Bjerkvig et al., 2005). In addition to the self-renew, CSCs can also differentiate into other cell types such as endothelial cells that are responsible for angiogenesis (Xiong et al., 2009; Ricci-Vitiani et al., 2010). Therefore, CSCs generate intra-tumor heterogeneity by differentiating a range of different cell types. On top of that, CSCs interact with epithelial–mesenchymal transformation (EMT) process to promote cancer cell invasion and migration (Kong et al., 2011). CSCs share some of same pathways with normal stem cells, such as Hedgehog signaling, Wnt/β-catenin, and Notch signaling pathways that maintain their self-renewal ability as well as confer the resistance to chemotherapy and radiotherapy in CRC (Baumann et al., 2008; Dylla et al., 2008; Colak et al., 2014; Yang et al., 2020). A Phase II clinical trial study confirmed the effect of metformin on CSCs in ovarian cancer, suggesting that epigenetic changes in tumor stroma may drive platinum sensitivity *in vitro* (Brown et al., 2020). Overall, the properties of CSCs endow them to complicate tumor microenvironment and enhance resistance to clinical therapy. Consequently, targeting CSCs could be a promising strategy for CRC treatment. For example, a phase I/II clinical trial employed CSC-loaded dendritic cells as vaccine using in metastatic CRC patients (https://clinicaltrials.gov/ct2/show/NCT02176746).

Given that CSCs function critically in cancer progression and therapy, we sought to emphasize and decipher the role of CSC markers in CRC development and therapy. Single-cell RNA sequencing data of CRC samples was analyzed for accurately annotating CRC marker genes. We identified two clusters through molecular subtyping based on CRC marker genes and parsing the difference of two clusters from various aspects including prognosis, immune microenvironment, biological pathways, and response to clinical therapy. Importantly, we established a CRC-based prognostic model which was reliable and effective for the survival prediction of CRC.

## Materials and methods

### The acquisition and preprocessing of bulk transcriptome data

The bulk RNA sequencing (RNA-seq) data of CRC samples and para-cancerous (normal) samples were obtained from The Cancer Genome Atlas (TCGA) database through Sangerbox platform in 30 September 2022 (named as TCGA dataset) (Tomczak et al., 2015; Shen et al., 2022). Microarray data of CRC samples (GSE17538 and GSE39582) were downloaded from Gene Expression Omnibus (GEO, specific links please see https://www.ncbi.nlm.nih.gov/geo/query/acc.cgi?acc=GSE17538, https://www.ncbi.nlm.nih.gov/geo/query/acc.cgi?acc=GSE39582) (Clough and Barrett, 2016).

For RNA-seq data of TCGA dataset, we removed the samples without clinical information and survival information. Ensembl IDs were transferred to gene symbols, and averaged expression levels were selected in the condition that one gene had multiple Ensembl IDs. Finally, 438 CRC samples were included in TCGA dataset (Supplementary Table S1). For microarray data, only samples with survival information were remained. Probes were transferred to gene symbols. We eliminated probes matching to multiple genes and selected averaged expression value when one gene had multiple probes. After preprocessing, a total of 232 and 556 CRC samples were remained in GSE17538 and GSE39582 datasets, respectively (Supplementary Tables S2, S3).

### The acquisition and processing of single-cell RNA sequencing data

Single-cell RNA sequencing (scRNA-seq) dataset (GSE200997) was downloaded from GEO. We retained 16 CRC samples in the dataset. ScRNA-seq data was filtered under following conditions: 1) each gene expressed at least in three cells; 2) each cell expressed at least 250 genes; 3) the percentage of mitochondria is less than 10%; 4) UMI of each cell >500 and log10(GenesPerUMI) > 0.8. After preprocessing, we analyzed the scRNA-seq data using Seurat R package according to following procedures (Gribov et al., 2010). Firstly, the expression profiles were log-normalized. Then we removed the batch effects of 16 tumor samples using FindVariableFeatures and FindIntegrationAnchors functions, and integrated data through IntegrateData function. Next, ScaleData function was conducted to scale data and identify the anchor for principal component analysis (PCA). Single cells were clustered with dim = 40 and Resolution = 0.5 based on FindNeighbors and FindClusters functions. Subsequently, we annotated the cell clusters according to the cell markers of eight cell types (B cells, T cells, CSCs, endothelial cells, fibroblasts, mast cells, myeloid cells, NK cells, and T cells) from CellMarker 2.0 and previous studies (Supplementary Table S4) (Peng et al., 2019; Zhang et al., 2019; Lee et al., 2021; Su et al., 2021). Finally, FindAllMarkers function was performed to discriminate differentially expressed genes (DEGs) among eight cell types.

### Analysis of cancer stemness

We used mRNA stemness index (mRNAsi) to measure cancer stemness at RNA expression level. Following a previous study, one-class logistic regression (OCLR) machine-learning algorithm was used to calculate the mRNAsi (Malta et al., 2018). The DEGs of CSCs were determined as CSC marker genes. Single sample gene set enrichment analysis (ssGSEA) calculated the score of CSC marker genes through GSVA R package (Hänzelmann et al., 2013). The mRNAsi and ssGSEA score of CSC marker genes were calculated for each tumor and normal sample in TCGA, GSE17538 and GSE39582 datasets. Pearson correlation analysis assessed the correlation between mRNAsi and CSC marker genes using Hmisc R package.

### Mutation analysis

Copy number variation (CNV) and single nucleotide variation (SNV) data were obtained from TCGA dataset, where SNV data had been processed by mutect2 software. Genes mutated in more than three tumor samples were retained and examined by Fisher’s exact test to determine significantly mutated genes (*p* < 0.05). The top 15 highly mutated genes were visualized.

### Molecular subtyping based on CSC marker genes

First of all, to identify disease-specific survival (DSS)-associated CSC marker genes (*p* < 0.05), we performed univariate Cox regression analysis. Then based on the expression profiles of DSS-associated CSC genes, tumor samples were subtyped by unsupervised consensus clustering in ConsensusClusterPlus R package with parameter settings were as follows: reps = 50, pItem = 0.8, pFeature = 1, and distance = Euclidean (Wilkerson and Hayes, 2010). We determined the optimal cluster number k referring to cumulative distribution function (CDF) curves, relative area change under CDF curves, and consensus matrix.

### Immune and pathway analysis

We obtained a group of gene sets of 28 immune cells, innate and adaptive immunity from previous research (Charoentong et al., 2017; He et al., 2018), and measured their enrichment scores using ssGSEA. ESTIMTAE algorithm evaluated the enrichment scores of immune cells and stromal cells, and outputted an ESTIMATE score representing the combined immune and stromal scores (Yoshihara et al., 2013). Microenvironment Cell Populations (MCP)-counter method was employed to assess the enrichment scores of nine immune cells and fibroblasts (Becht et al., 2016). We obtained a total of 47 immune checkpoint genes from a previous study (Danilova et al., 2019). For pathway analysis, hallmark pathways (h.all.v7.4. symbols.gmt) were collected from Molecular Signature Database (MSigDB) (Liberzon et al., 2015). The ssGSEA score for each pathway was calculated and compared between different groups.

### Assessment of oxidative stress

Oxidative stress related genes were collected from “GOBP_RESPONSE_TO_OXIDATIVE_STRESS” in MSigDB. Distribution of this GOBP gene set was analyzed in GSE17538, GSE39582, and TCGA. Pearson’s correlation analysis was performed to evaluate the relationship between risk score and oxidative stress. surv_cutpoint function embedded in survminer package was employed to determine the optimal cutoff and generate survival curves.

### Predicting the response to immunotherapy and chemotherapy

Tumor Immune Dysfunction and Exclusion (TIDE) algorithm (http://tide.dfci.harvard.edu/) was implemented to estimate the potential response of tumor samples to immune checkpoint inhibitors (ICIs) (Jiang et al., 2018). A higher TIDE score is positively correlated with a higher possibility of immune escape from ICIs. T cell exclusion and T cell dysfunction were examined by TIDE, and the enrichment scores of immunosuppressive cells including tumor-associated macrophages (TAM), myeloid-derived suppressor cells (MDSC), cancer-associated fibroblasts (CAF) were also calculated. The sensitivity to chemotherapeutic drugs was determined by the biochemical half maximal inhibitory concentration with pRRophetic R package (Geeleher et al., 2014).

### Constructing and validating a prognostic model

Under the threshold of |fold change| > 1.5 and false discovery rate (FDR) < 0.05, DEGs between different clusters were identified by limma R package (Ritchie et al., 2015). WebGestaltR package was used to annotate significantly enriched KEGG pathways for DEGs (Liao et al., 2019). Then TCGA dataset was randomly assigned at a ratio of 1:1 into training and testing groups. We screened DSS-associated DEGs through univariate Cox regression analysis in the training group (*p* < 0.01). To reach an optimal prognostic model, we conducted least absolute shrinkage and selection operator (Lasso) with glmnet package and stepwise Akaike information criterion (stepAIC) with MASS package to determine the most contributable genes to the model (Friedman et al., 2010; Zhang, 2016). The prognostic model was defined as: risk score = Σβi×Expi, where β indicates Lasso coefficients and Exp indicates the expression levels of prognostic genes (i).

According to the optimal cut-off determined by survminer R package, each tumor sample obtained a risk score and was classified into high-risk and low-risk groups. Survival time between two risk groups was shown by Kaplan-Meier survival analysis. Receiver operation characteristic (ROC) curve analysis was used to predict the efficiency of the prognostic model in predicting different survival time through timeROC R package (Blanche et al., 2013). The effectiveness and reliability of the model was validated in TCGA and GSE17538 datasets.

### Statistical analysis

The statistical analysis in this study was conducted and outputted by R software (version 4.1.0). Two-group statistical difference was examined by Wilcoxon test. Log-rank test was used in survival analysis and univariate Cox regression analysis. We considered *p* < 0.05 as statistically significant.

## Results

### Identification of CSC markers and their relation with mRNAsi

**FIGURE 1:**
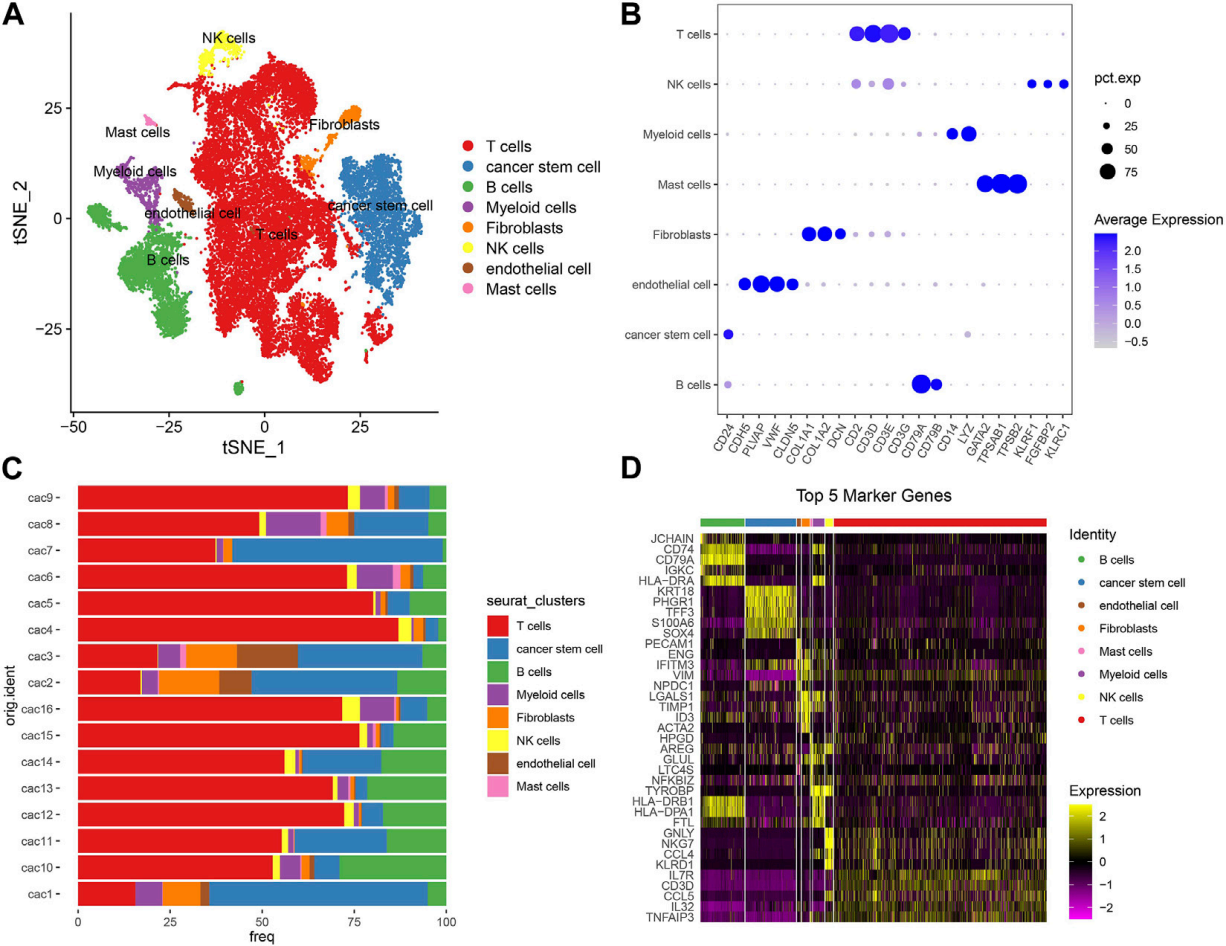
Analysis of scRNA-seq data. **(A)** T-SNE plot showed the distribution of eight cell types with different colors. **(B)** The expression of cell markers in different cell types. Pct. exp (dot) indicates the percentage of cells expressing marker genes. Blue color from light to dark indicates the expression from low to high. **(C)** The percentage of eight cell types in 16 tumor samples. 16 samples were indicated in the left and percentage was indicated in the bottom. **(D)** The top five DEGs of eight cell types. Yellow and purple represents high and low normalized expression respectively.

First of all, we used scRNA-seq data to identify different cell types based on their markers. Single cells were filtered to ensure the quality of data (see details in materials and methods). The gene counts, UMI counts, and mitochondrial percentage of 16 CRC samples before and after quality control were shown in Supplementary Figure S1. After quality control, we normalized the data and removed the batch effects based on highly variable genes (Supplementary Figure S2). Then single cells were further scaled and grouped into 21 clusters (Supplementary Figure S3). Using cell markers from CellMarker 2.0 and based on previous research, we annotated cells into eight cell types including B cells, T cells, endothelial cells, fibroblasts, mast cells, cancer stem cells, myeloid cells, and NK cells (Figures 1A, B; Supplementary Table S4). T cells contributed the largest proportion followed by B cells and CSCs in most tumor samples (Figure 1C). Subsequently, differentially expressed genes (DEGs) were identified from each cell type and the top five DEGs (bright yellow) were visualized (Figure 1D). For CSCs, we identified a total of 257 DEGs (Supplementary Table S5).

**FIGURE 2:**
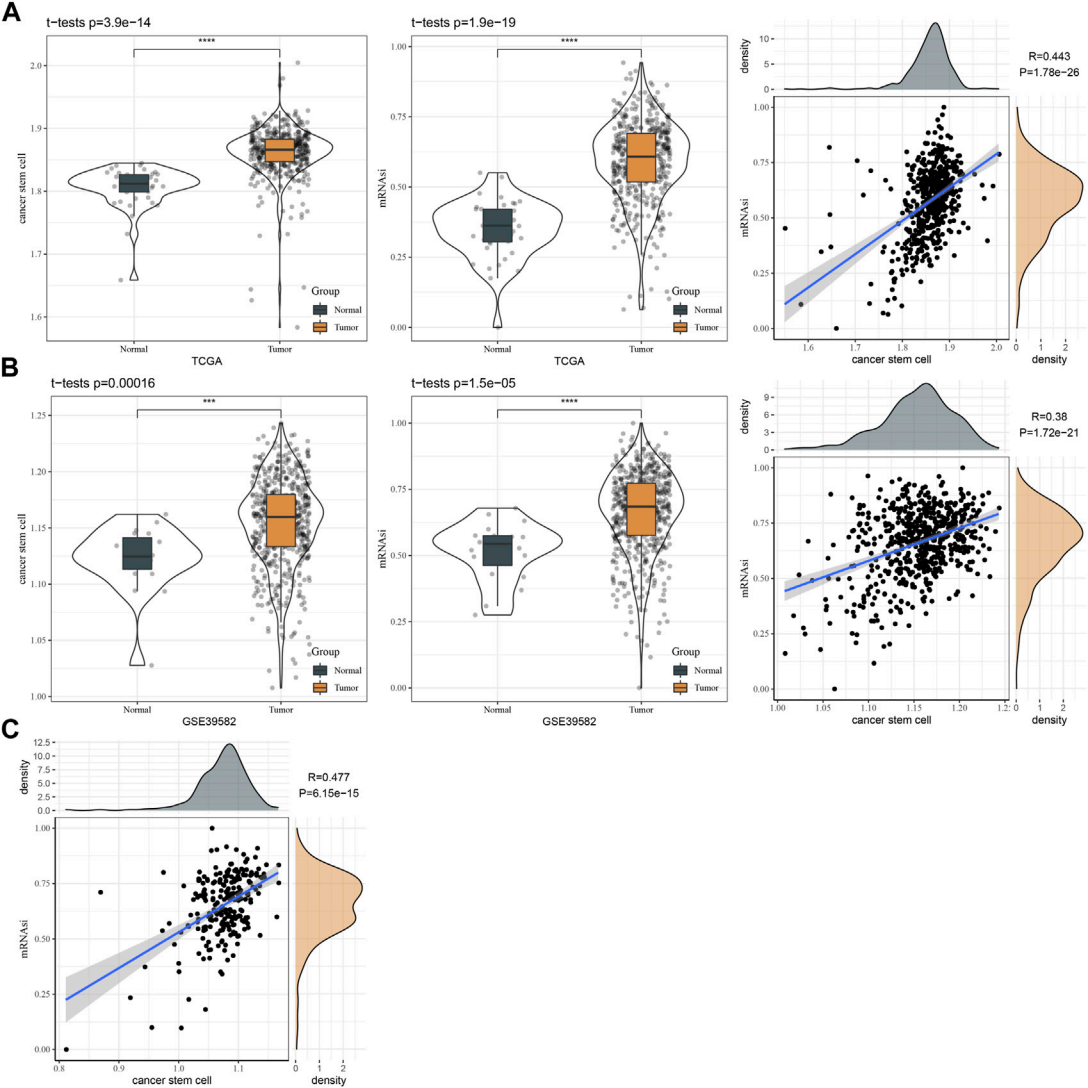
The relation between CSC markers and mRNAsi. **(A)** The ssGSEA score of CSC markers and mRNAsi in normal and tumor samples in TCGA dataset. Pearson correlation analysis between CSC markers and mRNAsi. **(B)** The ssGSEA score of CSC markers and mRNAsi in normal and tumor samples in GSE39582 dataset. Pearson correlation analysis between CSC markers and mRNAsi. **(C)** Pearson correlation analysis between CSC markers and mRNAsi in GSE17538 dataset.

To evaluate the reliability of 257 DEGs as marker genes of CSCs, we introduced mRNA stemness index (mRNAsi) to assess the correlation between 257 DEGs and mRNAsi. We firstly calculated the ssGSEA score of 257 DEGs and mRNAsi score in three independent datasets (TCGA, GSE17538, and GSE39582). By comparing the ssGSEA score and mRNAsi score in normal and cancer samples, we observed that cancer samples had higher scores of both ssGSEA and mRNAsi than normal samples in TCGA and GSE39582 datasets (*p* < 0.001, Figures 2A, B). In addition, the ssGSEA score of CSC marker genes was significantly positively related to mRNAsi score, with coefficients of 0.443, 0.380, and 0.477 in TCGA, GSE39582, and GSE17538, respectively (*p* < 0.0001, Figures 2A–C). Therefore, it is reasonable to determine the 257 DEGs as CSC marker genes.

### Identification of molecular subtypes based on CSC marker genes

To identify which CSC marker genes were associated with CRC progression, we performed univariate Cox regression based on DSS time. Of 257 CSC marker genes, we identified a total of 29 genes (20 risk genes and 9 protective genes) significantly associating with DSS (Supplementary Figure S4A; Supplementary Table S6). Within these 29 genes, 22 of them were differently expressed in cancer and para-cancerous samples (Supplementary Figure S4B). We also analyzed the gene mutations and genomic variations of 29 genes in cancer samples. PLEC, PLCG2, and LENG8 were the top three frequently mutated genes, with mutation frequencies of 10%, 7%, and 5%, respectively (Supplementary Figure S4C). CNV results showed that the frequency of gain of CNVs was larger than that of loss of CNVs (Supplementary Figure S4D). Especially, BRI3, CEBPB, HSPB1, and PLEC had frequencies of gain of CNVs over than 25%.

**FIGURE 3:**
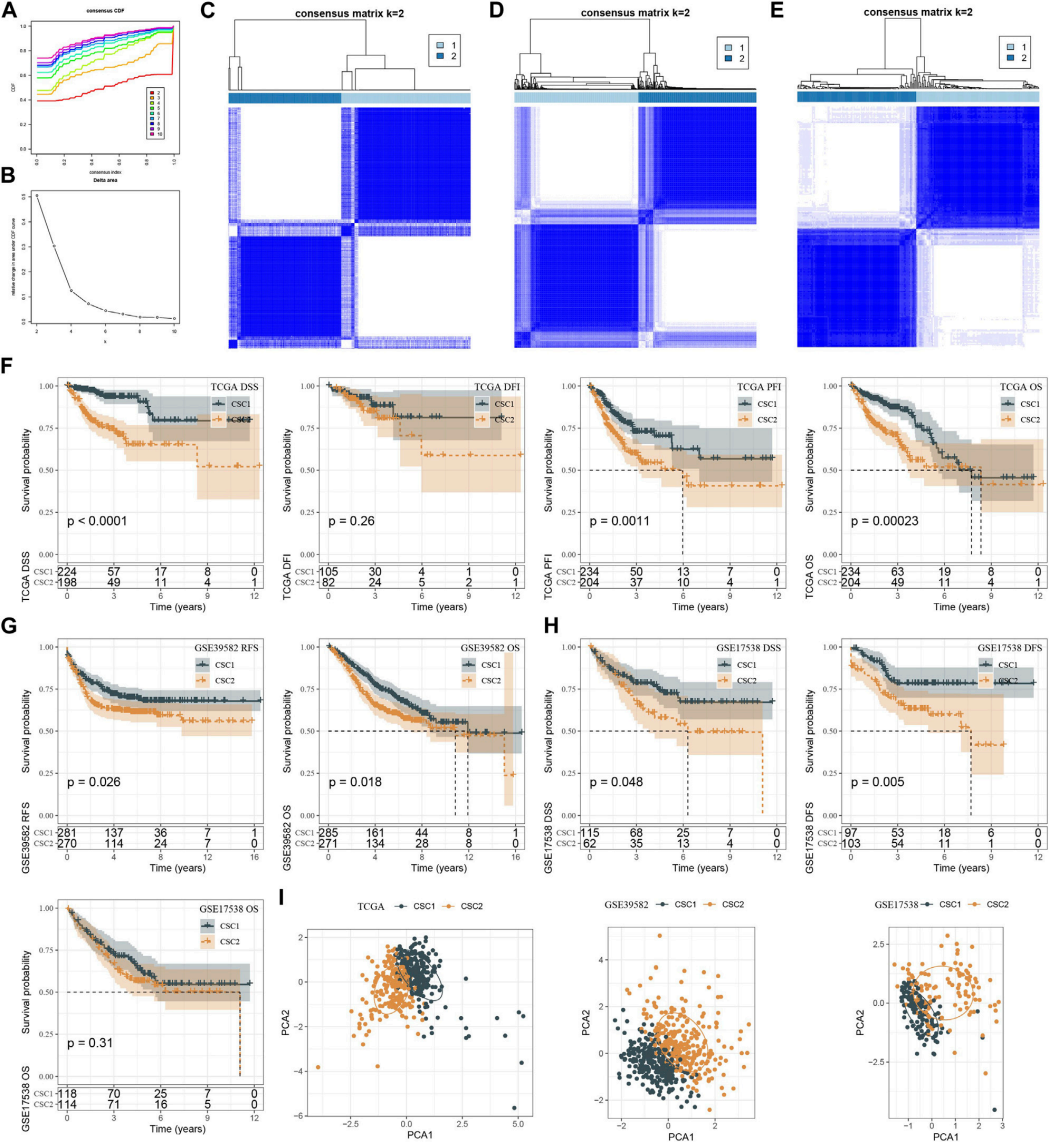
Molecular subtyping based on CSC markers. **(A, B)** CDF curves and relative change under CDF curves when cluster number k was 2–10 in TCGA dataset. **(C–E)** Consensus matrix when k = 2 in TCGA (C), GSE39582 **(D)** and GSE17538 **(E)** datasets. **(F–H)** Kaplan-Meier survival curves of CSC1 and CSC2 for different survival time in TCGA **(F)** GSE39582 **(G)** and GSE17538 **(H)** datasets. **(I)** PCA plots of CSC1 and CSC2 in three datasets. OS, overall survival; DSS, disease-specific survival; DFI, disease-free interval; PFI, progression-free interval; RFS, recurrence-free survival; DFS, disease-free survival.

Given that 29 CSC marker genes were closely related to patients’ prognosis, we then studied the role of these marker genes in CRC. Therefore, the expression profiles of 29 CSC marker genes in TCGA dataset were used in consensus clustering on CRC samples. According to CDF curve and consensus matrix, cluster number k = 2 was determined as the optimal and samples were classified into two clusters (CSC1 and CSC2) (Figures 3A–C). In GSE17538 and GSE39582 datasets, we used the same method to cluster samples and consensus matrix results showed that samples were evidently divided into two clusters (Figures 3D, E). Then we compared the prognosis of two clusters in three datasets. In TCGA dataset, CSC1 and CSC2 showed significantly different disease-specific survival (DSS) (*p* < 0.0001), progression-free interval (PFI) (*p* = 0.0011), and overall survival (OS) (*p* = 0.00023) (Figure 3F). In GSE39582 dataset, CSC1 and CSC2 had different prognosis on recurrence-free survival (RFS) (*p* = 0.026) and OS (*p* = 0.018) (Figure 3G). In GSE17538 dataset, two clusters had different DSS (*p* = 0.048) and disease-free survival (DFS) (*p* = 0.005), but no significant difference on OS (Figure 3H). Overall, CSC1 had better prognosis than CSC2. PCA plot presented that two clusters were evidently separated (Figure 3I). Therefore, we considered that the clustering of CRC samples based on 29 CSC marker genes was effective and reliable.

### Mutation and clinical characteristics of two clusters

We assessed the mutation data of TCGA dataset, and identified a total of 380 genes that had significantly higher mutation frequencies in CRC samples that in normal samples. The top 15 mutated genes were visualized, where XIRP2 and SCN1A had frequencies of over than 10% (Supplementary Figure S5A). However, there was no significant difference on tumor mutation burden between cancer and normal samples (Supplementary Figure S5B).

In addition, we compared the clinical characteristics including gender, age, stage Ⅰ to Ⅳ, TNM stage in two clusters. The distribution of different ages and genders did not show significant differences between two clusters (Supplementary Figure S5C, D). Noteworthy, CSC2 had markedly larger proportions of the samples with late stages than CSC1, with ratios of 0.14 and 0.09 in T4 stage, 0.24 and 0.12 in N2 stage, 0.22 and 0.10 in M1 stage, 0.21 and 0.09 in stage Ⅳ for CSC2 and CSC1 respectively (Supplementary Figure S5E–H). The findings suggested that CSC marker genes may have an influence on the progression of CRC.

### Immune microenvironment and oxidative stress differences of CSC1 and CSC2 clusters

**FIGURE 4:**
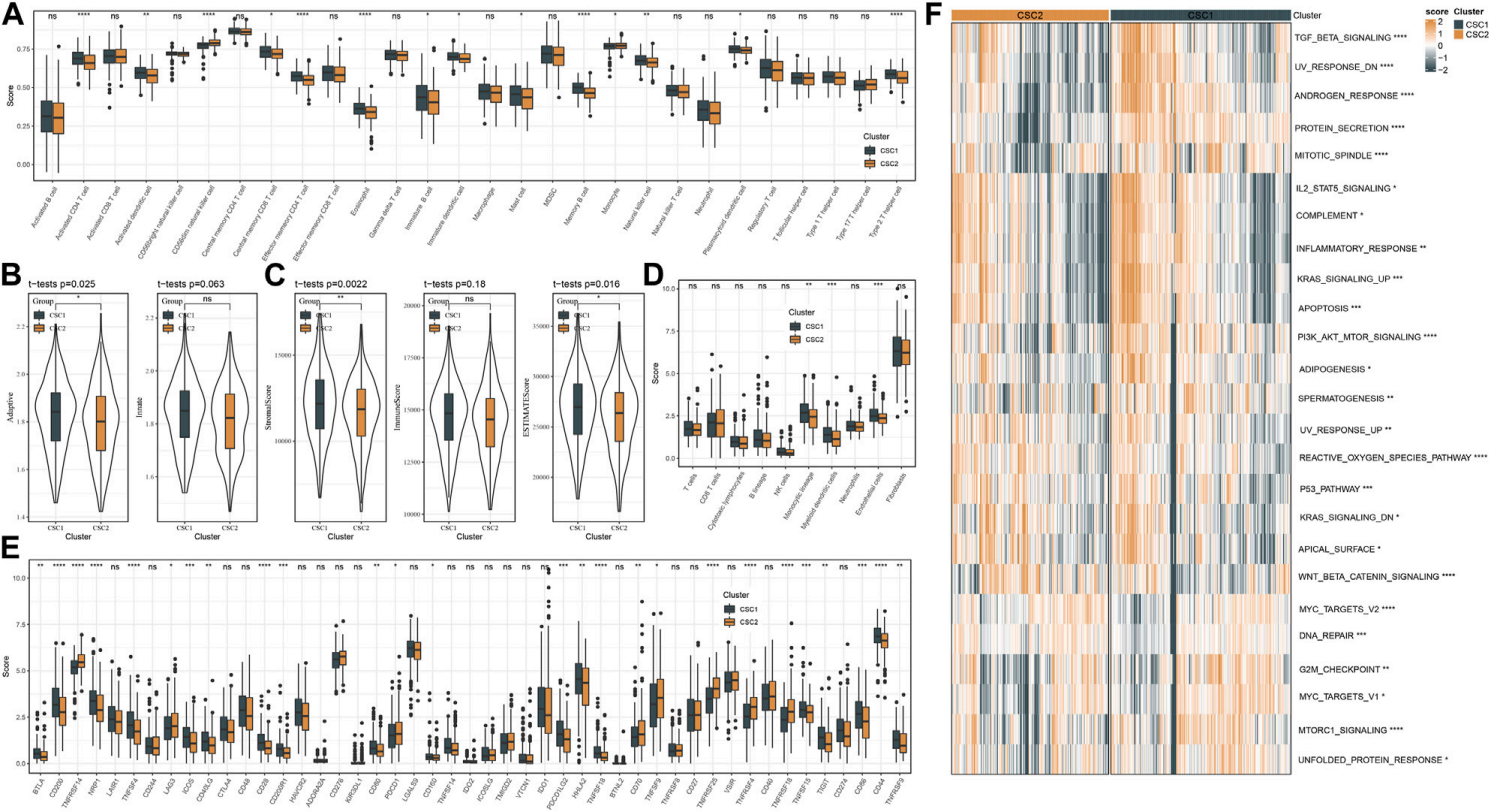
Immune microenvironment and pathway analysis of two clusters in TCGA dataset. **(A)** The estimated proportion of 28 immune-related cells by ssGSEA. **(B)** The ssGSEA score of adaptive and innate immune cells. **(C)** The stromal and immune scores measured by ESTIMATE. **(D)** MCP-counter assessed the enrichment score of 10 immune-related cells. **(E)** The expression levels of immune checkpoints. **(F)** A heatmap showed the z-score expression levels of differentially enriched pathways between two clusters. ns, not significant. *p < 0.05, **p < 0.01, ***p < 0.001, ****p < 0.0001.

We applied different methods to evaluate the immune microenvironment in CSC1 and CSC2. SsGSEA on the gene sets of 28 immune cells showed that 14 immune cells were differently enriched in two clusters, and CSC1 had higher enrichment scores in most of them such as natural killer cells, activated CD4 T cells, memory B cells (Figure 4A). In the response of adaptive and innate immunity, CSC1 also performed higher enrichment score than CSC2 but the difference was not significant in the innate immune response (Figure 4B). ESTIMATE analysis revealed higher infiltration of immune cells and stromal cells in CSC1 than that in CSC2 (Figure 4C). Moreover, MCP-counter manifested that of 10 immune-related cells, three cell types including monocytic lineage, endothelial cells, myeloid dendritic cells had noticeably higher enrichment scores in CSC1 compared with CSC2 (Figure 4D). The above results assessed by different methods were consistent with each other, suggesting a difference of immune cell infiltration and immune microenvironment between two clusters. Immune checkpoints are essential linkage of different immune cells for enhancing or inhibiting the cytotoxicity of immune cells. The expression levels of a total of 47 immune checkpoints were compared in two clusters. As a result, 26 of 47 immune checkpoints showed a significant difference between two clusters, with most of them were higher expressed in CSC1 (Figure 4E). Different expression levels of these immune checkpoints may contribute to the difference immune response between two clusters.

**FIGURE 5:**
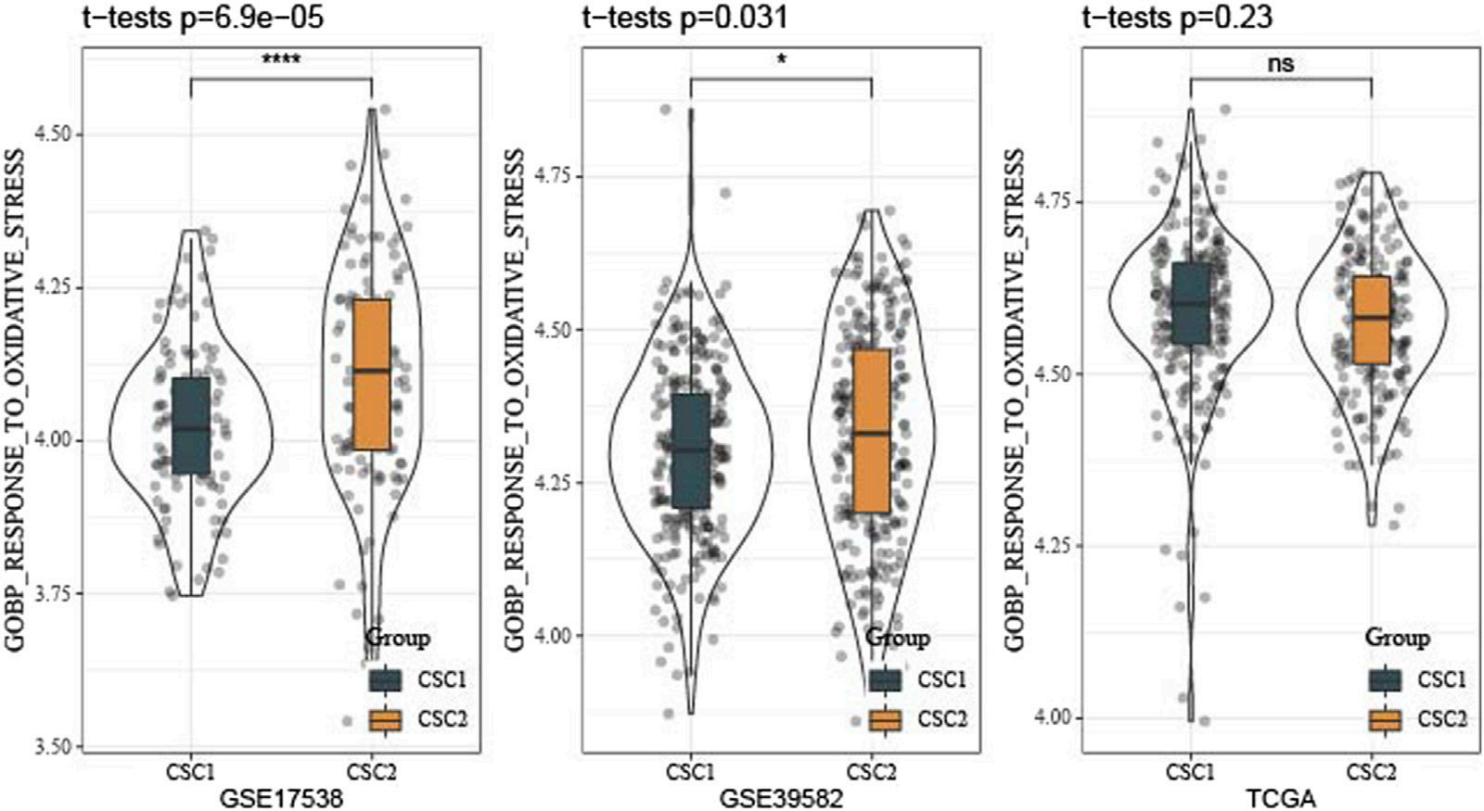
Difference of oxidative stress response of two clusters in TCGA, GSE17538 and GSE39582 datasets.

Furthermore, we reckoned the scores of hallmark pathways using ssGSEA to unveil the potential molecular mechanisms resulting in different prognosis in two clusters. As a result, 25 pathways were differently enriched between two clusters (Figure 4F). CSC1 displayed relatively enhanced activation of immune-correlated pathways, for example, complement and inflammatory response, IL2-STAT5 signaling, in accordant with the result of immune analysis. In addition, reactive oxygen species pathway, p53 signaling pathway and Wnt signaling pathway that were associated with oncogenesis were more activated in CSC2 compared with CSC1. Moreover, Figure 5 revealed that the score of “GOBP_RESPONSE_TO_OXIDATIVE_STRESS” was significantly increased in CSC2 in GSE17538 and GSE39582.

### The predicted response of two clusters to immunotherapy and chemotherapy

**FIGURE 6:**
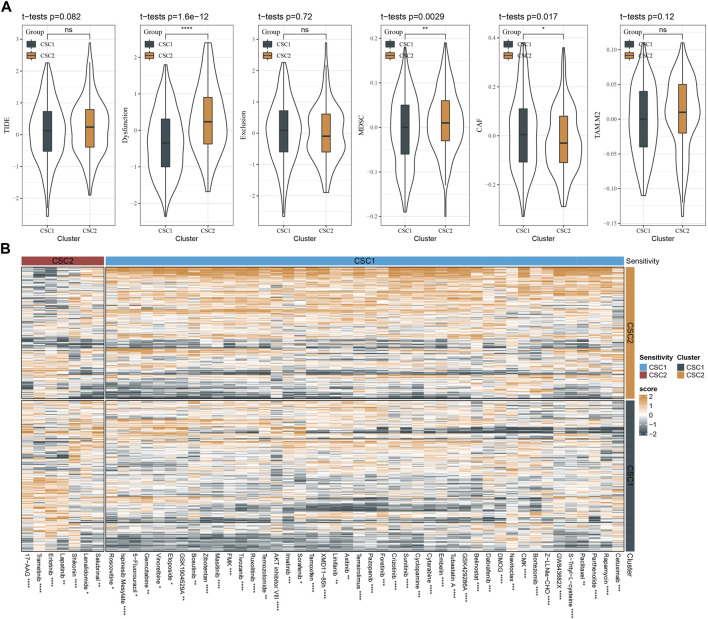
The sensitivity of two clusters to immunotherapy and chemotherapy. **(A)** TIDE analysis showed the scores of TIDE, T cell function, and infiltration of immunosuppressive cells. **(B)** A heatmap showed the estimated half maximal inhibitory concentration (IC50) of two clusters to different chemotherapeutic drugs. The drugs with significantly different IC50 in two clusters were visualized. MDSC, myeloid-derived suppressor cells; CAF, cancer-associated fibroblasts; TAM. M2, M2 tumor-associated macrophages. ns, not significant. *p < 0.05, **p < 0.01, ***p < 0.001, ****p < 0.0001.

We employed TIDE analysis to estimate the response to immunotherapy for two clusters. No significant difference was detected in TIDE score between two clusters. Higher TIDE score suggested lower sensitivity to immunotherapy. Although two clusters showed similar response to immunotherapy, CSC2 had higher score of T cell dysfunction and higher enrichment of MDSC, but lower score of CAF than CSC1 (Figure 6A). The function of T cells and infiltration levels of immunosuppressive cells (MDSC and CAF) can affect the response to immunotherapy.

In the predicted response of two clusters to chemotherapy, we evaluated a number of chemotherapeutic drugs using pRRophetic package. We identified a total of 51 chemotherapeutic drugs with different sensitivities to two clusters, where 44 drugs were more sensitive to CSC2 and 7 drugs were more sensitive to CSC1 (Figure 6B). Therefore, we inferred that CSC marker genes for molecular subtyping may be involved in the response to these chemotherapeutic drugs.

### Constructing a prognostic model based on DEGs between CSC1 and CSC2

In the above sections, we illustrated that CSC1 and CSC2 exhibited different prognosis, immune microenvironment and activated pathways. To identify which genes had a difference to the outcome of clusters, we performed differential analysis on the expression profiles between CSC1 and CSC2and screened DEGs under |log Foldchange (FC)| > 1.5 and FDR <0.05. Consequently, 598 DEGs including 214 downregulated genes and 384 upregulated genes were identified in CSC1 (Supplementary Figure S6A). The DEGs were significantly enriched in pathways like drug metabolism, TGF-β signaling pathway, and gap junction, as shown by KEGG pathway analysis (Supplementary Figure S6B).

**FIGURE 7:**
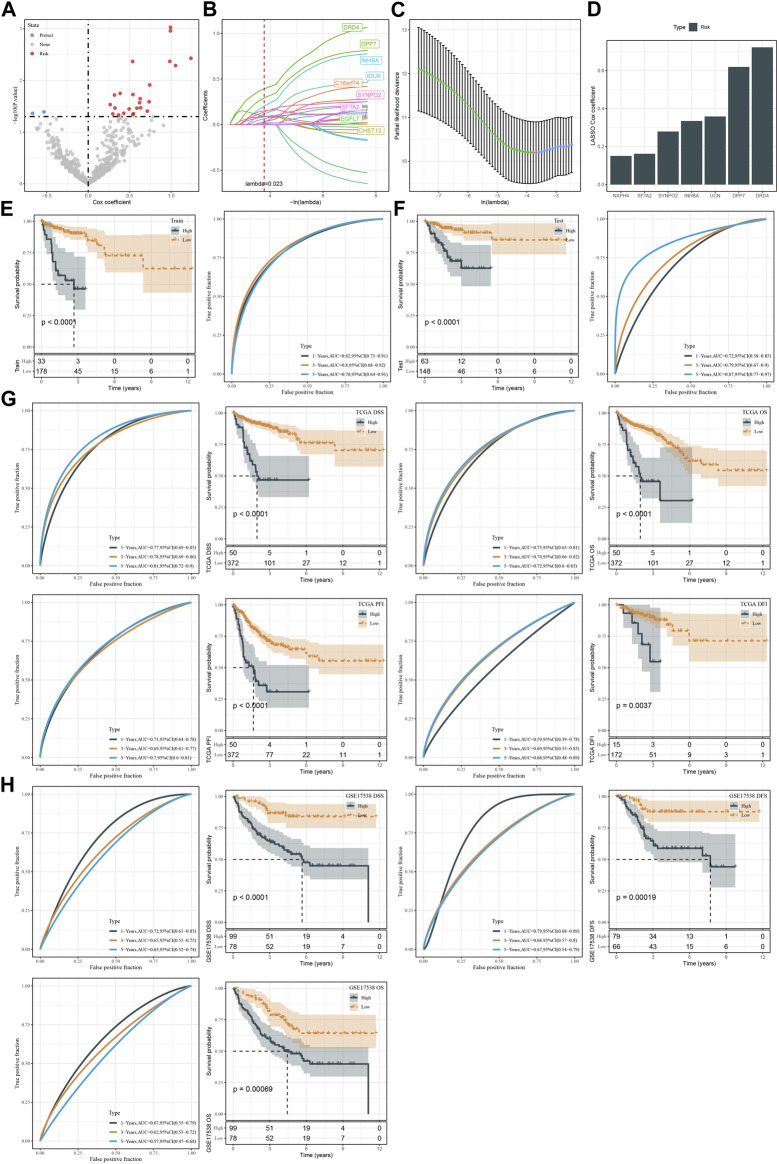
Construction and validation of the prognostic model. **(A)** Volcano plot of 26 CSC marker genes significantly associated with DSS in the training group. **(B, C)** Lasso regression analysis on 26 CSC marker genes. The coefficients of marker genes close to zero with the increasing value of lambda. Red dotted line and red dot represents the optimal lambda value of the model. **(D)** The Lasso coefficients of seven prognostic genes in the prognostic model. **(E, F)** Kaplan-Meier survival plots based on DSS of high-risk and low-risk groups in the TCGA training and testing groups. **(G)** Kaplan-Meier survival plots for DSS, OS, PFI, and DFI of high-risk and low-risk groups in TCGA dataset. **(H)** Kaplan-Meier survival plots for DSS, DFS and OS of high-risk and low-risk groups in GSE17538 dataset.

TCGA dataset was randomly divided into two groups, training and testing groups at a ratio of 1:1. To determine prognostic genes, we performed univariate Cox regression on 598 DEGs in the training group and screened 26 genes significantly related to DSS (Figure 7A). Furthermore, we used Lasso and stepAIC to decrease the number of prognostic genes for constructing a prognostic model efficiently applied in clinics. Lasso regression analysis determined 14 prognostic genes when the lambda value reached the optimal (lambda = 0.023, Figures 7B, C). Then stepAIC compressed 14 genes to 7 for the final prognostic genes in the model. Finally, the prognostic model was defined as: risk score = 0.722*DRD4 + 0.619*DPP7 +0.358*UCN +0.335*INHBA +0.162*SFTA2 + 0.279*SYNPO2 + 0.151*NXPH4.

We calculated risk score for each sample in TCGA dataset and classified samples into two groups (high risk and low risk) by the optimal cut-off determined by survminer package. In both training and testing groups, high-risk group showed evidently inferior DSS than low-risk group (*p* < 0.0001, Figures 7D, E). ROC curve analysis presented favorable AUC values of the model in predicting 1-year, 3-year, and 5-year DSS with over than 0.70 (Figures 7E, F). We verified the effectiveness of the prognostic model in the whole TCGA dataset. The model showed a good performance in predicting patient survival with different status (Figure 7G). Moreover, we used an independent dataset (GSE17538) to validate the reliability of the prognostic model (Figure 7H). In the DSS, DFS, and OS prediction and classification, the model showed a good efficiency (Figure 7H). In addition, samples with advanced T stage, N stage, M stage and Stage had a higher risk score in TCGA dataset, and similarly situation was observed in GSE17538 dataset samples along with Stage and Grade (Supplementary Figure S7).

### Pathway analysis of two risk groups

**FIGURE 8:**
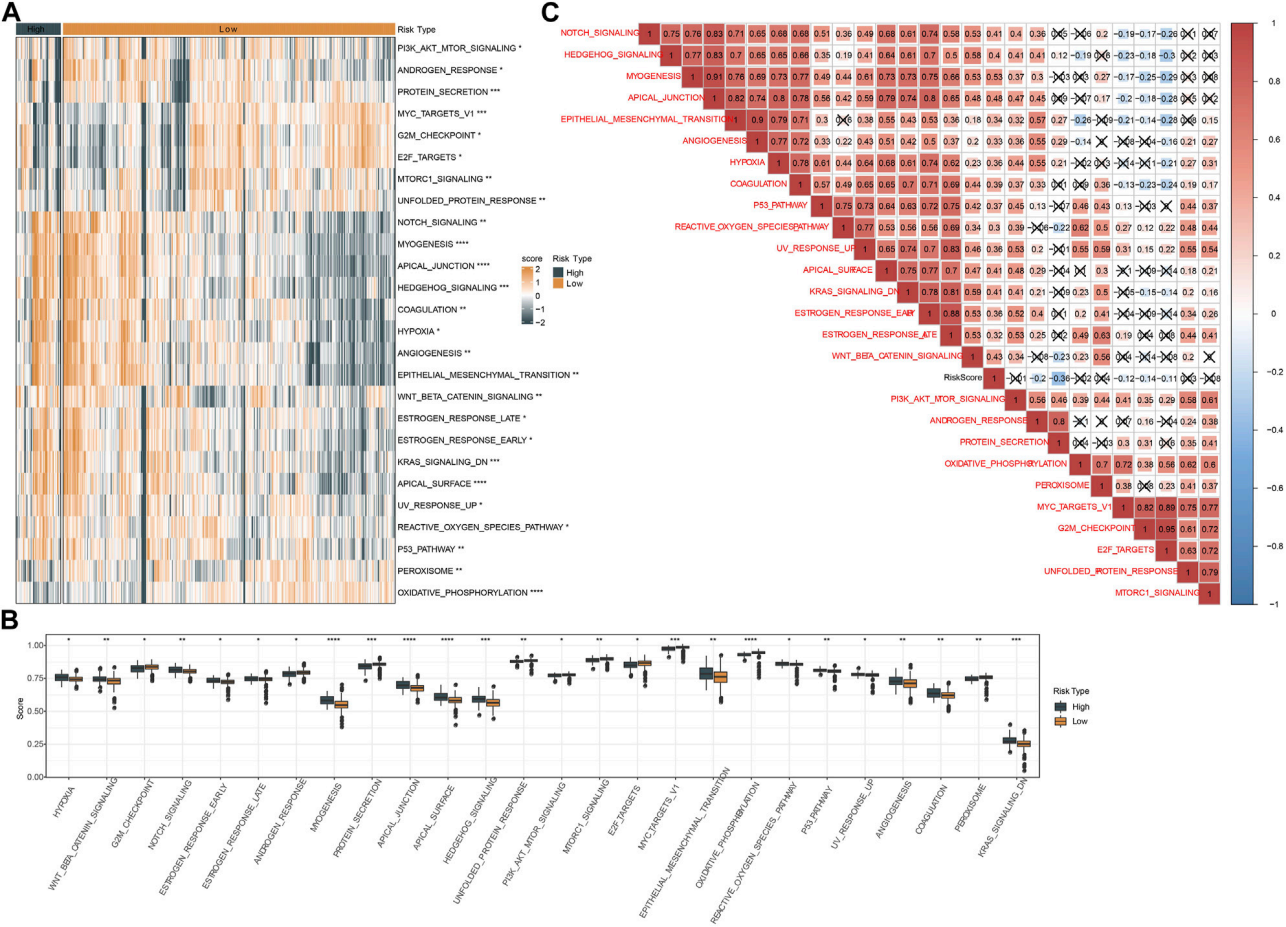
Analysis of hallmark pathways in two risk groups in TCGA dataset. **(A)** A heatmap displayed the normalized ssGSEA score of 26 pathways in two risk groups. **(B)** Box plots of ssGSEA score of 26 pathways in two risk groups. **(C)** Pearson correlation analysis between risk score and 26 pathways. Red and blue represents positive and negative correlation respectively. Fork indicates not significant.

Next, we assessed the enrichment of biological pathways in two risk groups to identify key pathways in tumor progression. Using ssGSEA we distinguished a total of 26 pathways that were differentially enriched in high-risk and low-risk groups (*p* < 0.05, Figures 8A, B). High-risk group exhibited relatively more activated oncogenic pathways than low-risk group, such as P53 signaling, angiogenesis, EMT, hypoxia, and Notch signaling pathways. Also, we examined the correlation between risk score and these pathways delineated by a heatmap. The result showed that risk score was positively correlated with most of these pathways, such as Notch signaling (R = 0.41), Hedgehog signaling (R = 0.40), apical junction (R = 0.48), EMT (R = 0.34), angiogenesis (R = 0.33), hypoxia (R = 0.36), P53 signaling (R = 0.37), reactive oxygen species pathway (R = 0.30), KRAS signaling down (R = 0.41) and Wnt-beta catnin signaling (R = 0.43) (Figure 8C).

### Oxidative stress analysis of two risk groups

**FIGURE 9:**
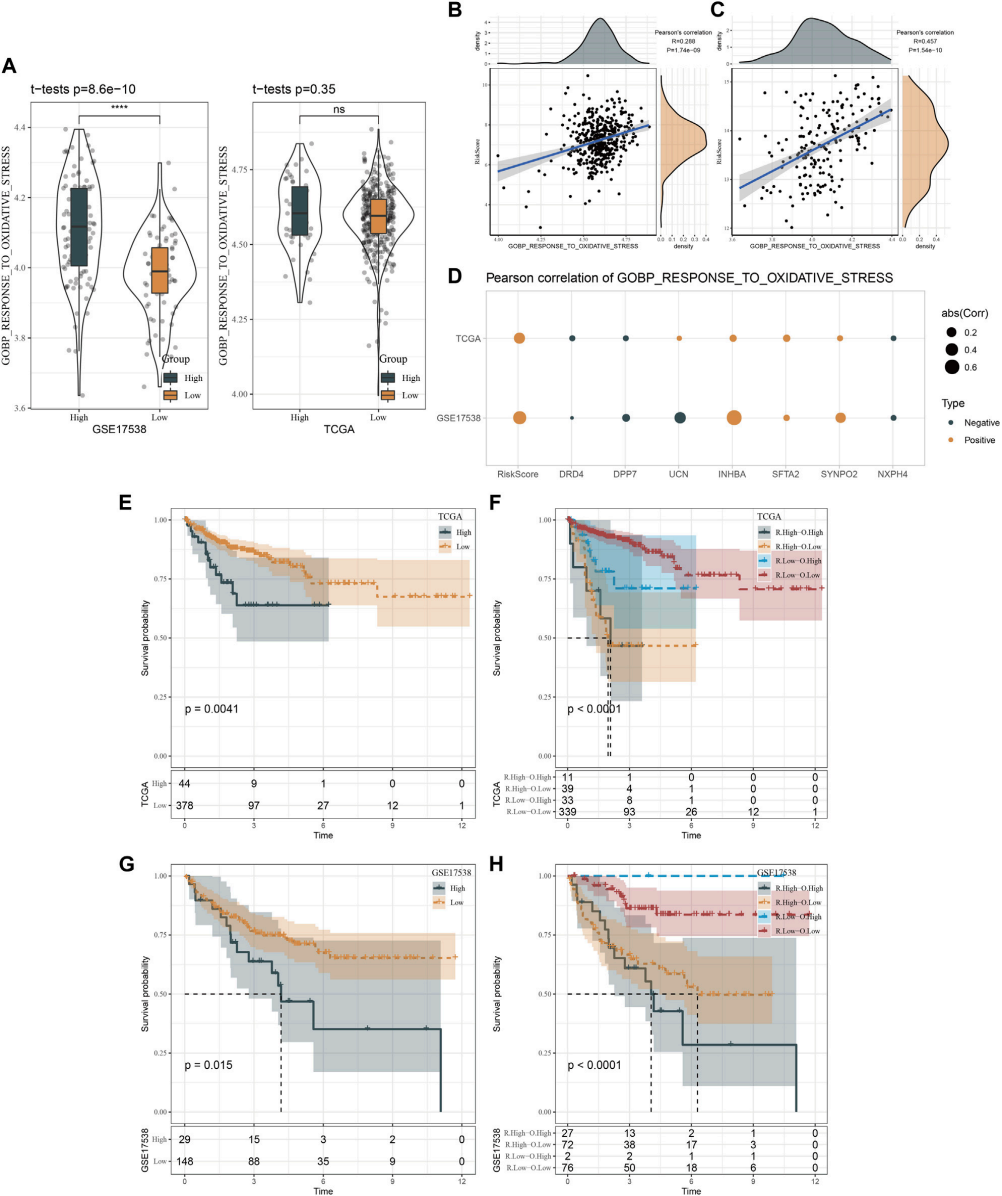
Oxidative stress analysis of two risk groups. **(A)**, Distribution of GOBP gene set in TCGA and GSE17538 datasets. **(B, C)**, Pearson correlation analysis of risk score with the response to oxidative stress in TCGA and GSE17538 datasets. **(D)**, Pearson correlation analysis of risk score and risk genes with the response to oxidative stress in TCGA and GSE17538 datasets. **(E, F)**, Kaplan-Meier survival curves of two risk groups or combination of risk score and oxidative stress in TCGA. **(G, H)**, Kaplan-Meier survival curves of two risk groups or combination of risk score and oxidative stress in GSE17538.

In addition, we emphatically analyzed the relation between risk score and response to oxidative stress. Figure 9A showed that high risk patients in GSE17538 possessed higher oxidative stress score that of low risk patients (*p* = 8.6e-10). Not surprisingly, risk score was positively correlated with GOBP response to oxidative stress in both TCGA (R = 0.288, *p* = 1.74e-09) and GSE17538 datasets (R = 0.457, *p* = 1.54e-10) (Figures 9B, C). Besides, GOBP response to oxidative stress was positively correlated with INHBA, SFTA2, and SYNPO2 both in TCGA and GSE17538 datasets (Figure 9D). Furthermore, we found that high patients exhibited dismal prognosis in TCGA (*p* = 0.0041) and GSE17538 (*p* = 0.015). Meanwhile, patients with high risk combined with high oxidative stress had the poorest prognosis (Figures 9E–H).

### The responses of two risk groups to immunotherapy and chemotherapy

**FIGURE 10:**
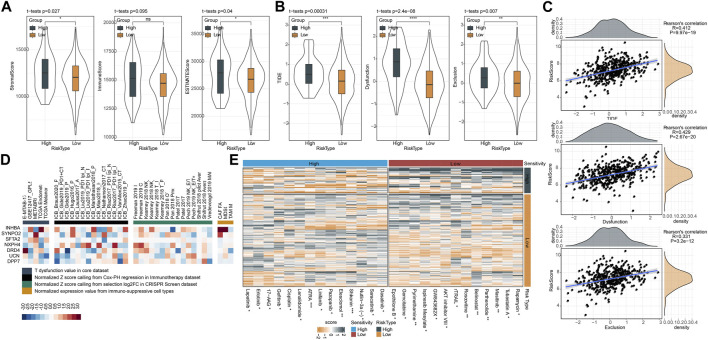
Prediction of response of two risk groups to immunotherapy and chemotherapy in TCGA dataset. **(A)** ESTIMATE analysis calculated the stromal score and immune score of two risk groups. **(B)** TIDE analysis predicted the response to immune checkpoint inhibitors. **(C)** Pearson correlation analysis of risk score with TIDE score, T cell dysfunction and T cell exclusion. **(D)** Enrichment of seven prognostic genes in T cell dysfunction score (how a gene interacts with cytotoxic T cells to influence patient survival outcome), ICB outcome (genes whose activities are correlated with ICB benefit), log-fold change (logFC) in CRISPR screens (the efficacy of lymphocyte-mediated tumor killing in cancer models) and T cell exclusion score (the gene expression levels in immunosuppressive cell types). Colors from red to blue indicates expression levels from high to low. **(E)** The estimated IC50 of two risk groups shown as heatmap. ns, not significant. *p < 0.05, **p < 0.01, ***p < 0.001, ****p < 0.0001.

Similarly, we applied ESTIMATE algorithm to evaluate immune cell infiltration and stromal cell infiltration in two risk groups. High-risk group manifested both higher stromal score and immune score than low-risk group, but immune score was not significantly different (Figure 10A). Generally, high immune infiltration is beneficial to immune response and prognosis. To address this puzzle, we further used TIDE analysis to predict the T cell function. As a result, high-risk group displayed more significant impairment of T cell function, where higher scores of T cell dysfunction and exclusion were shown in high-risk group in comparison to low-risk group (Figure 10B). Accordingly, high-risk group had higher TIDE score, indicating a higher immune escape possibility in the high-risk group. Supportively, risk score had a highly positive correlation with TIDE, T cell exclusion, T cell dysfunction, in accordant with the above findings (Figure 10C). The result also demonstrated that risk score was a potential indicator to predict the response to immunotherapy and T cell function. Furthermore, TIDE analysis generated the association of seven prognostic genes with T cell dysfunction, T cell exclusion, immune checkpoint blockade (ICB) outcome, and the efficiency of tumor killing in CRISPR-based models (Figure 10D). but, no significance of TMB was observed in high-risk group and low-risk group (Supplementary Figure S8A). 17 of 47 immune checkpoint genes expressions were enhanced in high-risk group (Supplementary Figure S8B). Drug sensitivity analysis revealed that two risk groups had different sensitivity to 27 chemotherapeutic drugs in which 14 drugs were more sensitive to high-risk group and 13 drugs were more sensitive to low-risk group (Figure 10E). Based on the above findings, we could speculate that risk score was predictive to indicate the response of CRC patients to different chemotherapeutic drugs.

## Discussion

The important roles of CSCs in cancer development and metastasis have been substantially demonstrated in the previous studies (Ayob and Ramasamy, 2018; Prager et al., 2019). In this study, we focused on CSCs and screened a group of CSC marker genes based on scRNA-seq data of CRC samples. Using the expression profiles of CSC marker genes, we subtyped CRC samples into two clusters (CSC1 and CSC2). We compared clinical characteristics, immune microenvironment and biological pathways in two clusters. Based on DEGs between CSC1 and CSC2, we established a prognostic model for predicting the prognosis and therapeutic response of CRC patients.

From scRNA-seq data, we detected 257 CSC marker genes that showed a consistent performance with mRNAsi score. The mRNAsi score denotes the stemness degree using expression profiles (Malta et al., 2018). Compared with the normal samples, the ssGSEA score of CSC marker genes and mRNAsi score were both significantly higher in CRC samples. Moreover, CSC marker genes was noticeably positively related to mRNAsi, which proved the reliability of identification method for CSC marker genes. Of 257 CSC marker genes, 29 of them were found to be significantly related to DSS. Some CSC marker genes showed extremely disparate expression levels between normal and tumor samples, such as PLCG2, DDX11, IER5L, LENG8, HAGHL and CPNE7 (Supplementary Figure S4). They were reported to contribute cancer progression and metastasis. For example, small cell lung cancer cells with PLCG2-high phenotype had stem-like and pro-metastatic features (Chan et al., 2021). DDX11 is essential for DNA replication and genomic stability, and is considered to have an oncogenic role (Mahtab et al., 2021). Some marker genes had a large percentage of gain of CNVs, particularly BRI3, CEBPB, HSPB1, and PLEC. CEBPB was identified as a prognostic biomarker in CRC and was found to participate CRC metastasis (Rahman et al., 2019; Shao et al., 2021). HSPB1 was highly expressed in tumor tissues correlating with poor prognosis in CRC (Nagaraja et al., 2012). However, a few studies reported the roles of these CSC marker genes in cancer stemness. We considered these CSC marker genes as important candidates for exploiting the mechanisms of CSCs in CRC.

To figure out the effects of 29 CSC marker genes in CRC prognosis and tumor microenvironment, we used consensus clustering to subtype tumor samples into two clusters (CSC1 and CSC2) based on the expression profiles of 29 genes. CSC1 had evidently longer disease-specific survival than CSC2 in both TCGA and GSE17538 datasets, indicating these CSC marker genes were involved in CRC progression. The speculation was further demonstrated by the distribution of clinical characteristics in two clusters. Tumor samples with late stages like T4, N2, M1, and stage Ⅳ had substantially higher proportion in CSC2 than that in CSC1. Therefore, the 29 CSC marker genes played important roles in CRC progression and metastasis.

Previous studies have outlined the intense linkage between CSCs and tumor microenvironment (Zhang et al., 2018; Khosravi et al., 2020), which enables targeting CSCs as a possible strategy to eradicate CRC (Jahanafrooz et al., 2020). The inflammatory cytokines, for instance, interferons (IFN), transforming growth factor (TGF)-β, tumor necrosis factor (TNF)-α secreted from immune cells especially TAMs of M2 phenotype exert profound effects on maintaining the stemness of CSCs and promoting immunosuppression through pathways such as NF-κB, STAT3, and Notch (Zhang et al., 2018; Bayik and Lathia, 2021). Reciprocally, CSCs can recruit TAMs through expressing immunomodulatory factors thereby intertwining with CSC stemness programming and transcriptional activity. In comparison on immune microenvironment between CSC1 and CSC2, we observed discrepant immune infiltration and stromal infiltration. CSC1 showed higher infiltration of immune cells such as monocytic lineage, dendritic cells, activated CD4 T cells, and natural killer cells than CSC2. Although two clusters had similar proportions of CD8 T cells and cytotoxic lymphocytes, CSC2 presented more severely impaired T cell function, which resulted in its poor prognosis. Notably, CSC2 also showed a higher proportion of MDSCs and M2 TAMs than CSC1. CSC-TAM and CSC-MDSC crosstalk promoting stemness and immunosuppression have been underlined by previous studies. TAMs can facilitate CSC phenotypes by mediators such as IL-6, TGF-β, and WNT ligands (Jinushi et al., 2011; Fan et al., 2014; Wan et al., 2014). Mechanistic analysis suggested that Nos2 and nitric oxide (NO) produced by MDSCs fostered CSC phenotypes via activating Notch and STAT3 pathways in cancer cells (Peng et al., 2016; Ouzounova et al., 2017). In addition, pathway analysis revealed that tumor-associated pathways such as TGF-β and Wnt-β catenin signaling, cell cycle-related pathways such as MYC, and immune-related pathways such as inflammatory response and IL2-STAT5 signaling were distinctly enriched in CSC1 and CSC2, which was responsible for their different anti-cancer response and prognosis. It's worth noting that difference in TIDE score between two clusters was not been observed. Although two clusters showed similar response to immunotherapy, CSC2 had higher score of T cell dysfunction and higher enrichment of MDSC, but lower score of CAF than CSC1. The function of T cells and infiltration levels of immunosuppressive cells (MDSC and CAF) can affect the response to immunotherapy (Tamura, 2018).

Given the discrepant clinical characteristics and molecular features between CSC1 and CSC2, we established a prognostic model based on DEGs between two clusters. Finally, we confirmed seven prognostic genes in the model, including DRD4, DPP7, UCN, INHBA, SFTA2, SYNPO2, and NXPH4. DRD4 belongs to dopamine receptor (DR) family that is associated with the progressive phenotypes of cancer (Wang et al., 2019). A machine learning study identified DRD4 as a survival-related candidate gene for CRC patients (Lee et al., 2022). DPP7 is a member of dipeptidyl peptidase (DPP) family, a high expression of which was related to a favorable prognosis in breast cancer (Choy et al., 2021). Ahluwalia et al. developed a four-gene signature where DPP7 was included for predicting survival of CRC patients (Ahluwalia et al., 2019). INHBA is a member of TGF-β superfamily and can accelerate migration and invasion of gastric cancer cells via TGF-β signaling pathway (Chen et al., 2019). INHBA was identified as an independent risk factor for both OS and DFS in colon cancer (Li et al., 2020). SFTA2 was also identified as a prognostic gene for colon cancer (Li et al., 2018; Gong et al., 2020). Other three genes were few reported in the relation with CRC.

The seven-gene prognostic model effectively classified CRC patients into two risk groups. Specifically, high-risk group showed evidently inferior OS and DSS than low-risk group. In addition to different prognosis, two risk groups also manifested different activation of biological pathways and different response to immunotherapy and chemotherapy. Oncogenic pathways such as Wnt-β catenin, hypoxia, EMT, angiogenesis, Hedgehog signaling, and Notch signaling were more activated in high-risk group than in low-risk group. Simultaneously, risk score showed a positive correlation with the above pathways. High-risk group was less responsive to ICB therapy, resulting from T cell exclusion and dysfunction. Moreover, two risk groups performed different sensitivity to different chemotherapeutic drugs.

## Conclusion

In conclusion, this study harnessed scRNA-seq data to identify CSC marker genes in CRC and demonstrated the important roles of CSC marker genes in CRC progression by delineating CSC-based subtyping (CSC1 and CSC2). The 29 CSC marker genes were considered as candidate genes for further exploring the mechanism of CSC in CRC. Importantly, we developed a seven-gene prognostic model for not only predicting OS and DSS of CRC patients, but also guiding immunotherapy and chemotherapy in clinics for CRC treatment.

## Data Availability

The datasets presented in this study can be found in online repositories. The names of the repository/repositories and accession number(s) can be found in the article/supplementary material.

## Abbreviations

CSCs, cancer stem cells; CAF, cancer-associated fibroblasts; CRC, colorectal cancer; CNV, copy number variation; CDF, cumulative distribution function; DEGs, differentially expressed genes; DFS, disease-free survival; DSS, disease-specific survival; EMT, epithelial–mesenchymal transformation; FDR, false discovery rate; GEO, Gene Expression Omnibus; Lasso, least absolute shrinkage and selection operator; MCP, Microenvironment Cell Populations; mRNAsi, mRNA stemness index; MDSC, myeloid-derived suppressor cells; OCLR, one-class logistic regression; PCA, principle component analysis; ROC, receiver operation characteristic; RFS, recurrence-free survival; SNV, Single nucleotide variation; ssGSEA, single sample gene set enrichment analysis; scRNA-seq, Single-cell RNA sequencing; stepAIC, stepwise Akaike information criterion; TCGA, The Cancer Genome Atlas; TGF, transforming growth factor; TIDE, Tumor Immune Dysfunction and Exclusion; TNF, tumor necrosis factor; TAM, tumor-associated macrophages.
