# Supplementary material for: Constructing a cancer stem cell related prognostic model for predicting immune landscape and drug sensitivity in colorectal cancer
Source: Front Pharmacol. 2023 Jun 12;14:1200017. doi: 10.3389/fphar.2023.1200017 (PMC10292801; doi:10.3389/fphar.2023.1200017)
Supplement: Supplementary file 1 [file DataSheet1.pdf]

Table S1. The clinical information of TCGA dataset

Samples	type	OS.time	OS	PFI.time	PFI	DFI.time	DFI	DSS.time	DSS	A3_T	A4_N	A5_M	A6_Stage	Gender	Age	Age1
TCGA-AA-	COAD	212	0	212	0	212	0	212	0	T3	N0	M0	II	Male	42	<=65
TCGA-DM	COAD	2895	0	2895	0	NA	NA	2895	0	T3	N0	M0	II	Male	63	<=65
TCGA-DM	COAD	4126	0	4126	0	NA	NA	4126	0	T3	N1	M0	III	Male	75	>65
TCGA-A6-	COAD	926	0	926	0	926	0	926	0	T2	N0	M0	I	Male	82	>65
TCGA-CK-	COAD	2523	0	2523	0	2523	0	2523	0	T3	N0	NA	II	Female	87	>65
TCGA-AA-	COAD	638	0	638	0	638	0	638	0	T2	N2	M0	III	Male	38	<=65
TCGA-AD-	COAD	834	0	834	0	834	0	834	0	T3	N0	NA	NA	Male	58	<=65
TCGA-A6-	COAD	1127	0	976	1	NA	NA	1127	0	T2	N0	M0	I	Female	75	>65
TCGA-CM	COAD	153	1	31	1	NA	NA	153	1	T3	N1	M1	IV	Male	80	>65
TCGA-DM	COAD	1348	1	1348	0	NA	NA	1348	NA	T3	N0	M0	II	Male	68	>65
TCGA-A6-	COAD	1331	0	735	1	NA	NA	1331	0	T3	N2	M1	IV	Male	71	>65
TCGA-A6-	COAD	598	0	598	0	598	0	598	0	T4	N1	NA	III	Male	43	<=65
TCGA-QG-	COAD	256	1	256	0	NA	NA	256	0	T3	N1	NA	III	Male	71	>65
TCGA-A6-	COAD	996	0	996	0	NA	NA	996	0	T4	N0	M0	II	Female	71	>65
TCGA-WS	COAD	2130	0	2130	0	2130	0	2130	0	T3	N0	NA	II	Female	52	<=65
TCGA-AA-	COAD	730	0	730	0	730	0	730	0	T3	N0	M0	II	Female	79	>65
TCGA-CM	COAD	488	0	488	0	NA	NA	488	0	T3	N0	M0	II	Male	74	>65
TCGA-G4-	COAD	858	1	517	1	NA	NA	858	1	T3	N1	M1	IV	Male	75	>65
TCGA-AA-	COAD	518	0	518	0	518	0	518	0	T2	N0	M0	I	Female	78	>65
TCGA-QG-	COAD	1003	0	1003	0	1003	0	1003	0	T3	N0	NA	II	Female	61	<=65
TCGA-AA-	COAD	547	0	547	0	547	0	547	0	T3	N0	M0	II	Male	66	>65
TCGA-DM	COAD	2988	0	2988	0	NA	NA	2988	0	T3	N0	M0	II	Male	75	>65
TCGA-CM	COAD	396	0	396	0	NA	NA	396	0	T3	N0	M0	II	Male	67	>65
TCGA-D5-	COAD	521	0	521	0	NA	NA	521	0	T3	N2	M0	III	Female	79	>65
TCGA-CK-	COAD	475	0	475	0	NA	NA	475	0	T4	N2	M0	III	Female	48	<=65
TCGA-A6-	COAD	627	0	627	0	NA	NA	627	0	T3	N0	M0	II	Female	69	>65
TCGA-3L-	COAD	475	0	475	0	475	0	475	0	T2	N0	M0	I	Female	61	<=65
TCGA-G4-	COAD	1883	0	1883	0	NA	NA	1883	0	T3	N1	M1	IV	Male	66	>65
TCGA-A6-	COAD	1014	0	1014	0	NA	NA	1014	0	T3	N0	M0	II	Male	73	>65
TCGA-A6-	COAD	995	0	509	1	509	1	995	0	T4	N0	M0	II	Male	78	>65
TCGA-DM	COAD	236	1	236	0	NA	NA	236	NA	T3	N0	M0	II	Female	70	>65
TCGA-AA-	COAD	1247	0	1005	1	1005	1	1247	0	T2	N0	M0	I	Female	73	>65
TCGA-A6-	COAD	612	0	612	0	612	0	612	0	T3	N0	NA	II	Male	74	>65
TCGA-D5-	COAD	1701	0	1701	0	1701	0	1701	0	T3	N1	M0	III	Male	63	<=65
TCGA-T9-	COAD	362	0	81	1	81	1	362	0	T3	N0	M0	II	Male	82	>65
TCGA-G4-	COAD	1935	0	1935	0	1935	0	1935	0	T3	N1	M0	III	Male	69	>65
TCGA-AA-	COAD	454	0	365	1	NA	NA	454	0	T3	N2	M0	III	Female	78	>65
TCGA-CM	COAD	397	0	337	1	NA	NA	397	0	T3	N2	M1	IV	Male	35	<=65
TCGA-AA-	COAD	395	0	395	0	NA	NA	395	0	T3	N0	M0	II	Male	83	>65
TCGA-AU-	COAD	441	0	441	0	NA	NA	441	0	T3	N0	M0	II	Female	80	>65
TCGA-A6-	COAD	766	0	686	1	NA	NA	766	0	T3	N0	M1	IV	Male	56	<=65
TCGA-D5-	COAD	621	0	621	0	NA	NA	621	0	T2	N0	M0	I	Male	53	<=65
TCGA-A6-	COAD	740	1	740	0	NA	NA	740	0	T3	N2	M0	III	Female	68	>65
TCGA-DM	COAD	405	1	80	1	NA	NA	405	NA	T3	N0	M0	II	Male	82	>65
TCGA-F4-	COAD	1456	0	1456	0	1456	0	1456	0	T3	N0	M0	II	Male	64	<=65
TCGA-AA-	COAD	943	0	943	0	943	0	943	0	T2	N0	M0	I	Male	76	>65
TCGA-AA-	COAD	580	0	580	0	580	0	580	0	T2	N0	M0	I	Male	80	>65
TCGA-A6-	COAD	255	0	255	0	NA	NA	255	0	T3	N0	M0	II	Male	31	<=65
TCGA-A6-	COAD	1419	0	1419	0	1419	0	1419	0	T3	N1	M0	III	Female	82	>65
TCGA-CM	COAD	337	0	337	0	NA	NA	337	0	T3	N0	M0	II	Female	75	>65
TCGA-DM	COAD	1849	1	1849	0	NA	NA	1849	NA	T3	N0	M0	II	Male	75	>65
TCGA-AD-	COAD	370	0	370	0	370	0	370	0	T2	N0	NA	I	Male	67	>65
TCGA-CM	COAD	699	0	699	0	NA	NA	699	0	T3	N1	M0	III	Male	72	>65
TCGA-D5-	COAD	474	0	474	0	NA	NA	474	0	T3	N0	M0	II	Male	49	<=65
TCGA-A6-	COAD	671	0	529	1	NA	NA	671	0	T3	N0	M0	II	Female	84	>65
TCGA-AA-	COAD	914	0	914	0	914	0	914	0	T3	N0	M0	II	Male	82	>65
TCGA-F4-	COAD	47	0	47	0	NA	NA	47	0	T3	N2	NA	III	Male	60	<=65
TCGA-CA-	COAD	377	0	377	0	377	0	377	0	T3	N0	M0	II	Female	52	<=65
TCGA-AA-	COAD	335	1	335	1	NA	NA	335	1	T4	N2	M1	IV	Female	67	>65
TCGA-CK-	COAD	643	1	292	1	NA	NA	643	1	T1	N0	M0	I	Female	71	>65
TCGA-AY-	COAD	542	0	542	0	542	0	542	0	T3	N1	M0	III	Female	66	>65
TCGA-A6-	COAD	617	0	617	0	617	0	617	0	T4	N0	NA	II	Male	82	>65
TCGA-AA-	COAD	1004	0	1004	0	1004	0	1004	0	T4	N0	M0	II	Female	60	<=65
TCGA-AA-	COAD	30	1	30	1	NA	NA	30	1	T3	N0	M0	II	Female	78	>65
TCGA-NH	COAD	543	0	543	0	NA	NA	543	0	T3	N0	NA	II	Female	53	<=65
TCGA-D5-	COAD	275	0	275	0	NA	NA	275	0	T4	N1	M0	III	Male	65	<=65
TCGA-D5-	COAD	406	0	406	0	NA	NA	406	0	T3	N0	M0	II	Male	67	>65
TCGA-AA-	COAD	424	0	424	0	424	0	424	0	T3	N0	M0	II	Male	72	>65
TCGA-AA-	COAD	518	0	518	0	NA	NA	518	0	T3	N0	M0	II	Female	74	>65
TCGA-A6-	COAD	1881	1	612	1	NA	NA	1881	1	T3	N1	M1	IV	Male	56	<=65
TCGA-AA-	COAD	549	0	549	0	NA	NA	549	0	T4	N1	M0	III	Male	80	>65
TCGA-AA-	COAD	31	0	31	0	NA	NA	31	0	T3	N0	M0	II	Female	83	>65
TCGA-AZ-	COAD	1503	1	1503	0	1503	0	1503	0	T3	N0	NA	II	Female	77	>65
TCGA-CM	COAD	518	0	518	0	NA	NA	518	0	T4	N1	M1	IV	Female	59	<=65
TCGA-F4-	COAD	262	1	262	0	NA	NA	262	0	T3	N2	M0	III	Female	61	<=65
TCGA-D5-	COAD	1661	1	1007	1	NA	NA	1661	1	T3	N1	M0	III	Female	60	<=65
TCGA-CK-	COAD	2134	1	480	1	NA	NA	2134	0	T3	N0	M0	II	Female	79	>65
TCGA-AA-	COAD	1035	0	1035	0	NA	NA	1035	0	T3	N0	M0	II	Male	65	<=65
TCGA-AA-	COAD	608	0	608	0	608	0	608	0	T3	N2	M0	III	Female	72	>65
TCGA-CM	COAD	457	0	335	1	NA	NA	457	0	T3	N0	M0	II	Female	63	<=65
TCGA-CM	COAD	395	0	395	0	NA	NA	395	0	T3	N0	M0	II	Female	84	>65
TCGA-CM	COAD	456	0	456	0	NA	NA	456	0	T3	N2	M0	III	Female	57	<=65
TCGA-F4-	COAD	1260	0	336	1	336	1	1260	0	T2	N0	M0	I	Female	59	<=65
TCGA-D5-	COAD	614	0	386	1	NA	NA	614	0	T3	N0	M0	II	Male	69	>65
TCGA-G4-	COAD	672	0	672	0	NA	NA	672	0	T2	N1	NA	III	Female	60	<=65
TCGA-AA-	COAD	822	0	822	0	822	0	822	0	T2	N0	M0	I	Female	89	>65
TCGA-G4-	COAD	4051	0	4051	0	4051	0	4051	0	T3	N1	M0	III	Female	49	<=65
TCGA-QL-	COAD	666	0	666	0	666	0	666	0	T2	N0	NA	I	Female	84	>65
TCGA-G4-	COAD	2275	0	1003	1	1003	1	2275	0	T3	N0	M0	II	Male	84	>65
TCGA-AZ-	COAD	1002	0	688	1	NA	NA	1002	0	T3	N1	M0	III	Male	84	>65
TCGA-CK-	COAD	2599	0	2599	0	2599	0	2599	0	T3	N1	M0	III	Female	68	>65
TCGA-F4-	COAD	1047	0	1047	0	1047	0	1047	0	T3	N0	M0	II	Female	58	<=65
TCGA-G4-	COAD	2506	0	439	1	NA	NA	2506	0	T3	N2	M1	IV	Female	55	<=65
TCGA-A6-	COAD	672	0	183	1	NA	NA	672	0	T4	N2	NA	III	Male	80	>65
TCGA-CK-	COAD	1493	1	1493	0	1493	0	1493	0	T2	N0	NA	I	Male	81	>65
TCGA-AA-	COAD	1856	0	1856	0	1856	0	1856	0	T3	N0	M0	II	Male	68	>65
TCGA-A6-	COAD	1020	0	1020	0	1020	0	1020	0	T3	N0	M0	II	Female	80	>65
TCGA-AZ-	COAD	899	1	899	0	899	0	899	0	T2	N1	NA	NA	Female	77	>65
TCGA-AZ-	COAD	680	1	680	1	NA	NA	680	1	T3	N0	M1	IV	Male	61	<=65
TCGA-AM	COAD	14	0	14	0	NA	NA	14	0	T4	N2	M1	IV	Female	59	<=65
TCGA-CM	COAD	457	0	457	0	NA	NA	457	0	T3	N1	M0	III	Female	60	<=65
TCGA-AA-	COAD	1216	0	1216	0	1216	0	1216	0	T2	N0	M0	I	Male	73	>65
TCGA-G4-	COAD	419	0	419	0	NA	NA	419	0	NA	N0	NA	I	Male	50	<=65
TCGA-AU-	COAD	824	0	824	0	NA	NA	824	0	T2	N0	M0	I	Female	69	>65
TCGA-AA-	COAD	761	0	761	0	761	0	761	0	T2	N0	NA	NA	Male	65	<=65
TCGA-D5-	COAD	775	0	775	0	775	0	775	0	T4	N0	M0	NA	Female	68	>65
TCGA-A6-	COAD	735	0	735	0	735	0	735	0	T3	N1	M0	III	Male	66	>65
TCGA-AY-	COAD	525	0	396	1	396	1	525	0	T2	N0	M0	I	Female	74	>65
TCGA-F4-	COAD	16	0	16	0	16	0	16	0	T3	N0	M0	II	Female	77	>65
TCGA-AA-	COAD	945	0	945	0	945	0	945	0	T3	N1	M0	III	Female	53	<=65
TCGA-DM	COAD	1162	1	1162	0	NA	NA	1162	NA	T3	N2	M0	III	Female	68	>65
TCGA-AA-	COAD	1431	0	1431	0	1431	0	1431	0	T3	N0	M0	II	Male	84	>65
TCGA-F4-	COAD	1074	0	1074	0	1074	0	1074	0	T2	N0	M0	I	Male	45	<=65
TCGA-QG-	COAD	952	0	952	0	952	0	952	0	T2	N0	M0	I	Male	61	<=65
TCGA-DM	COAD	3641	0	3641	0	NA	NA	3641	0	T3	N0	M0	II	Female	71	>65
TCGA-AA-	COAD	911	0	91	1	91	1	911	0	T3	N0	M0	II	Female	81	>65
TCGA-D5-	COAD	229	0	229	0	NA	NA	229	0	T2	N0	M0	I	Female	51	<=65
TCGA-AA-	COAD	580	0	580	0	580	0	580	0	T2	N0	M0	I	Male	57	<=65
TCGA-CM	COAD	670	0	670	0	NA	NA	670	0	T3	N1	M0	III	Female	39	<=65
TCGA-AA-	COAD	882	0	882	0	882	0	882	0	T3	N0	M0	II	Female	78	>65
TCGA-CK-	COAD	669	0	669	0	NA	NA	669	0	T3	N1	NA	III	Male	81	>65
TCGA-D5-	COAD	312	0	312	0	NA	NA	312	0	T2	N0	M0	I	Female	79	>65
TCGA-AY-	COAD	1004	0	1004	0	NA	NA	1004	0	T3	N1	M1	IV	Female	65	<=65
TCGA-CA-	COAD	383	0	383	0	383	0	383	0	T3	N0	M0	II	Male	56	<=65
TCGA-A6-	COAD	293	0	293	0	293	0	293	0	T3	N1	M0	III	Male	59	<=65
TCGA-A6-	COAD	718	0	495	1	NA	NA	718	0	T3	N2	M1	IV	Male	46	<=65
TCGA-NH	COAD	588	0	588	0	588	0	588	0	T3	N2	M0	III	Male	69	>65
TCGA-CM	COAD	427	0	427	0	NA	NA	427	0	T1	N0	M0	I	Male	74	>65
TCGA-AD-	COAD	176	1	176	0	176	0	176	0	T4	N2	NA	III	Male	84	>65
TCGA-AA-	COAD	579	0	579	0	NA	NA	579	0	T3	N0	M1	IV	Male	68	>65
TCGA-AY-	COAD	6	0	6	0	NA	NA	6	0	T3	N2	NA	III	Male	47	<=65
TCGA-AD-	COAD	2532	1	1516	1	1516	1	2532	0	T3	N0	M0	II	Male	76	>65
TCGA-CM	COAD	457	0	457	0	NA	NA	457	0	T2	N0	M0	I	Male	60	<=65
TCGA-F4-	COAD	1309	0	1309	0	1309	0	1309	0	T3	N2	M0	III	Female	51	<=65
TCGA-D5-	COAD	380	0	380	0	NA	NA	380	0	T3	N0	M0	NA	Female	45	<=65
TCGA-AA-	COAD	822	0	822	0	NA	NA	822	0	T3	N1	M0	III	Male	75	>65
TCGA-QG-	COAD	1301	0	1301	0	1301	0	1301	0	T4	N1	NA	III	Female	64	<=65
TCGA-A6-	COAD	742	0	742	0	742	0	742	0	T2	N0	M0	I	Male	82	>65
TCGA-AA-	COAD	700	0	700	0	NA	NA	700	0	T2	N0	M0	I	Male	78	>65
TCGA-A6-	COAD	494	1	301	1	NA	NA	494	1	T3	N2	NA	III	Female	34	<=65
TCGA-AA-	COAD	914	0	914	0	914	0	914	0	T3	N0	M0	II	Male	72	>65
TCGA-AA-	COAD	580	0	580	0	580	0	580	0	T2	N0	M0	I	Male	73	>65
TCGA-CM	COAD	974	0	974	0	NA	NA	974	0	T3	N0	M0	II	Male	44	<=65
TCGA-D5-	COAD	460	0	460	0	NA	NA	460	0	T3	N1	NA	III	Female	80	>65
TCGA-QG-	COAD	896	0	896	0	896	0	896	0	T3	N2	NA	III	Female	55	<=65
TCGA-AA-	COAD	730	0	730	0	730	0	730	0	T3	N0	M0	II	Male	84	>65
TCGA-A6-	COAD	888	0	888	0	888	0	888	0	T3	N2	M0	III	Male	73	>65
TCGA-AY-	COAD	543	0	543	0	543	0	543	0	T3	N0	M0	II	Female	55	<=65
TCGA-D5-	COAD	1706	0	1706	0	1706	0	1706	0	T3	N0	M0	II	Male	73	>65
TCGA-D5-	COAD	555	0	555	0	NA	NA	555	0	T3	N0	M0	II	Male	61	<=65
TCGA-AA-	COAD	731	0	731	0	NA	NA	731	0	T3	N0	M0	II	Female	86	>65
TCGA-SS-	COAD	1829	0	506	1	506	1	1829	0	T4	N0	M0	II	Female	44	<=65
TCGA-CM	COAD	335	0	0	1	NA	NA	335	0	T4	N1	M1	IV	Female	63	<=65
TCGA-F4-	COAD	1442	0	1442	0	1442	0	1442	0	T3	N0	M0	II	Female	70	>65
TCGA-AZ-	COAD	159	1	159	0	NA	NA	159	0	T4	N1	M0	III	Male	77	>65
TCGA-CK-	COAD	3780	0	3780	0	NA	NA	3780	0	T2	N0	NA	I	Female	88	>65
TCGA-CA-	COAD	383	0	383	0	383	0	383	0	T3	N1	M0	III	Male	63	<=65
TCGA-A6-	COAD	1111	0	1111	0	1111	0	1111	0	T3	N0	M0	II	Male	62	<=65
TCGA-D5-	COAD	365	0	365	0	NA	NA	365	0	T4	N2	M0	III	Male	77	>65
TCGA-AA-	COAD	214	1	214	1	NA	NA	214	1	T3	N0	M0	II	Male	75	>65
TCGA-CM	COAD	366	0	366	0	NA	NA	366	0	T3	N2	M0	III	Female	78	>65
TCGA-AA-	COAD	1096	0	1096	0	1096	0	1096	0	T3	N0	M0	II	Male	85	>65
TCGA-A6-	COAD	685	0	685	0	NA	NA	685	0	T2	N0	M0	I	Male	61	<=65
TCGA-DM	COAD	2475	1	1929	1	NA	NA	2475	NA	T3	N0	M0	II	Male	74	>65
TCGA-AA-	COAD	882	0	882	0	NA	NA	882	0	T3	N0	M0	II	Male	63	<=65
TCGA-A6-	COAD	758	1	257	1	NA	NA	758	1	T4	N1	M0	III	Female	55	<=65
TCGA-A6-	COAD	726	0	726	0	726	0	726	0	T3	N1	M0	III	Female	65	<=65
TCGA-AZ-	COAD	1910	1	304	1	NA	NA	1910	1	T3	N0	NA	II	Male	43	<=65
TCGA-AA-	COAD	1127	0	1127	0	1127	0	1127	0	T2	N0	M0	I	Male	79	>65
TCGA-AA-	COAD	1096	0	1096	0	1096	0	1096	0	T3	N0	M0	II	Male	65	<=65
TCGA-D5-	COAD	308	0	308	0	NA	NA	308	0	T3	N1	M0	III	Male	76	>65
TCGA-G4-	COAD	2003	1	872	1	NA	NA	2003	1	T3	N1	M1	IV	Female	54	<=65
TCGA-A6-	COAD	887	0	887	0	887	0	887	0	T3	N1	NA	III	Female	40	<=65
TCGA-F4-	COAD	1087	0	1087	0	1087	0	1087	0	T3	N0	M0	II	Male	51	<=65
TCGA-AY-	COAD	29	1	29	0	NA	NA	29	0	T1	N0	NA	I	Female	63	<=65
TCGA-AA-	COAD	1127	0	1127	0	1127	0	1127	0	T3	N0	M0	II	Male	67	>65
TCGA-DM	COAD	427	1	320	1	NA	NA	427	NA	T3	N2	M0	III	Male	68	>65
TCGA-DM	COAD	1518	1	1518	0	NA	NA	1518	NA	T3	N0	M0	II	Male	88	>65
TCGA-CM	COAD	884	0	884	0	884	0	884	0	T2	N1	M0	III	Female	82	>65
TCGA-AZ-	COAD	206	1	206	0	206	0	206	0	T2	N0	NA	I	Male	72	>65
TCGA-CA-	COAD	388	0	388	0	388	0	388	0	T3	N0	M0	II	Male	57	<=65
TCGA-D5-	COAD	377	0	377	0	NA	NA	377	0	T3	N0	M0	II	Female	77	>65
TCGA-F4-	COAD	1024	0	1024	0	1024	0	1024	0	T1	N0	M0	I	Female	54	<=65
TCGA-G4-	COAD	2047	1	2047	0	NA	NA	2047	0	T3	N0	M0	II	Female	90	>65
TCGA-CA-	COAD	376	0	376	0	376	0	376	0	T3	N0	M0	II	Male	45	<=65
TCGA-CK-	COAD	534	0	21	1	NA	NA	534	0	T4	N1	M0	III	Female	46	<=65
TCGA-CM	COAD	365	0	365	0	NA	NA	365	0	T3	N1	M0	III	Female	48	<=65
TCGA-AA-	COAD	578	0	578	0	NA	NA	578	0	T3	N1	M1	IV	Male	80	>65
TCGA-AA-	COAD	791	0	791	0	791	0	791	0	T3	N1	M0	III	Female	87	>65
TCGA-G4-	COAD	2792	0	2564	1	NA	NA	2792	0	T3	N0	M0	II	Female	77	>65
TCGA-CA-	COAD	386	0	386	0	386	0	386	0	T3	N0	M0	II	Female	42	<=65
TCGA-D5-	COAD	435	0	435	0	NA	NA	435	0	T3	N0	M0	II	Male	68	>65
TCGA-A6-	COAD	824	0	824	0	824	0	824	0	T3	N1	M0	III	Male	55	<=65
TCGA-G4-	COAD	1422	1	1422	0	NA	NA	1422	0	T3	N0	M0	II	Male	90	>65
TCGA-CK-	COAD	61	0	61	0	NA	NA	61	0	T3	N1	M1	IV	Female	45	<=65
TCGA-DM	COAD	3561	0	484	1	NA	NA	3561	0	T3	N2	M0	III	Male	50	<=65
TCGA-AA-	COAD	1096	0	1096	0	1096	0	1096	0	T2	N0	M0	I	Female	71	>65
TCGA-CM	COAD	394	0	394	0	NA	NA	394	0	T3	N0	M0	II	Male	39	<=65
TCGA-DM	COAD	4270	0	4270	0	NA	NA	4270	0	T3	N0	M0	II	Female	67	>65
TCGA-A6-	COAD	1286	0	1286	0	1286	0	1286	0	T3	N1	M0	III	Female	43	<=65
TCGA-CM	COAD	457	0	457	0	NA	NA	457	0	T2	N0	M0	I	Female	73	>65
TCGA-A6-	COAD	442	1	364	1	NA	NA	442	0	T3	N0	M0	II	Male	79	>65
TCGA-G4-	COAD	792	0	789	1	NA	NA	792	0	T3	N1	NA	III	Male	65	<=65
TCGA-F4-	COAD	1087	0	1087	0	1087	0	1087	0	T2	N0	M0	I	Male	60	<=65
TCGA-AA-	COAD	489	0	489	0	489	0	489	0	T3	N1	M0	III	Male	58	<=65
TCGA-AA-	COAD	1915	0	1915	0	1915	0	1915	0	T3	N0	M0	II	Female	54	<=65
TCGA-CM	COAD	396	0	396	0	396	0	396	0	T3	N0	M0	II	Male	58	<=65
TCGA-CA-	COAD	371	0	371	0	371	0	371	0	T3	N0	M0	II	Male	65	<=65
TCGA-CM	COAD	883	0	883	0	NA	NA	883	0	T3	N0	M0	II	Female	46	<=65
TCGA-A6-	COAD	734	0	734	0	734	0	734	0	T3	N0	M0	II	Male	62	<=65
TCGA-CM	COAD	427	0	427	0	NA	NA	427	0	T2	N0	M0	I	Female	77	>65
TCGA-CK-	COAD	1561	0	1561	0	1561	0	1561	0	T3	N0	NA	II	Female	58	<=65
TCGA-AZ-	COAD	59	1	59	0	NA	NA	59	NA	T2	N1	M0	III	Female	55	<=65
TCGA-D5-	COAD	596	0	579	1	NA	NA	596	0	T3	N1	M0	III	Male	60	<=65
TCGA-AY-	COAD	573	0	573	0	NA	NA	573	0	T3	N2	M1	IV	Male	44	<=65
TCGA-CM	COAD	335	0	335	0	NA	NA	335	0	T3	N1	M0	III	Female	70	>65
TCGA-AA-	COAD	1612	0	1612	0	1612	0	1612	0	T3	N0	M0	II	Male	71	>65
TCGA-CA-	COAD	435	0	354	1	NA	NA	435	0	T3	N0	M0	II	Male	77	>65
TCGA-AZ-	COAD	1776	0	1776	0	NA	NA	1776	0	T3	N0	M0	II	Male	61	<=65
TCGA-CM	COAD	306	0	306	0	NA	NA	306	0	T3	N0	M0	II	Male	58	<=65
TCGA-D5-	COAD	146	1	123	1	NA	NA	146	1	T3	N1	NA	III	Male	64	<=65
TCGA-AD-	COAD	650	0	650	0	650	0	650	0	T2	N0	M0	I	Female	81	>65
TCGA-CM	COAD	457	0	457	0	NA	NA	457	0	T2	N0	M0	I	Female	36	<=65
TCGA-AA-	COAD	212	0	59	1	59	1	212	0	T4	N0	M0	II	Male	64	<=65
TCGA-AA-	COAD	1065	0	1065	0	1065	0	1065	0	T2	N0	M0	I	Male	73	>65
TCGA-DM	COAD	2821	1	1920	1	NA	NA	2821	NA	T3	N0	M0	II	Male	80	>65
TCGA-DM	COAD	4233	0	4233	0	NA	NA	4233	0	T3	N0	M0	II	Female	60	<=65
TCGA-A6-	COAD	1321	0	1321	0	1321	0	1321	0	T3	N0	NA	II	Male	78	>65
TCGA-DM	COAD	3648	0	3648	0	NA	NA	3648	0	T3	N0	M0	II	Female	72	>65
TCGA-G4-	COAD	2424	0	2424	0	2424	0	2424	0	T2	N0	M0	I	Male	78	>65
TCGA-CM	COAD	669	0	669	0	NA	NA	669	0	T2	N0	M0	I	Female	48	<=65
TCGA-CM	COAD	915	0	915	0	NA	NA	915	0	T3	N0	M0	II	Female	68	>65
TCGA-AA-	COAD	669	0	669	0	669	0	669	0	T2	N0	M0	I	Female	55	<=65
TCGA-AA-	COAD	1643	0	1643	0	NA	NA	1643	0	T3	N0	M0	II	Female	74	>65
TCGA-G4-	COAD	804	0	804	0	NA	NA	804	0	T3	N1	NA	III	Male	73	>65
TCGA-AA-	COAD	546	0	546	0	546	0	546	0	T3	N0	M0	II	Female	62	<=65
TCGA-NH	COAD	302	1	6	1	NA	NA	302	1	T4	N2	NA	III	Male	58	<=65
TCGA-AZ-	COAD	43	1	43	1	NA	NA	43	1	T4	N2	M1	IV	Male	37	<=65
TCGA-D5-	COAD	1381	1	722	1	722	1	1381	1	T3	N2	NA	NA	Male	83	>65
TCGA-AA-	COAD	1066	0	762	1	762	1	1066	0	T3	N0	M0	II	Female	82	>65
TCGA-AA-	COAD	1124	0	1124	0	1124	0	1124	0	T3	N0	M0	II	Male	66	>65
TCGA-DM	COAD	1094	1	1094	0	NA	NA	1094	NA	T3	N1	M0	III	Male	73	>65
TCGA-NH	COAD	389	0	389	0	NA	NA	389	0	T4	N1	M1	IV	Female	66	>65
TCGA-AZ-	COAD	3042	1	2270	1	NA	NA	3042	1	T3	N0	M0	II	Male	68	>65
TCGA-5M-	COAD	1200	0	810	1	NA	NA	1200	0	T3	N0	M0	II	Male	76	>65
TCGA-A6-	COAD	763	0	654	1	NA	NA	763	0	T3	N1	M1	IV	Female	56	<=65
TCGA-A6-	COAD	1711	1	1678	1	1678	1	1711	0	T3	N1	M0	III	Male	57	<=65
TCGA-AA-	COAD	943	0	943	0	943	0	943	0	T1	N0	M0	I	Female	83	>65
TCGA-DM	COAD	805	1	805	0	NA	NA	805	NA	T3	N2	M0	III	Male	78	>65
TCGA-AA-	COAD	791	0	791	0	791	0	791	0	T2	N1	M0	III	Male	70	>65
TCGA-D5-	COAD	540	0	540	0	NA	NA	540	0	T3	N0	M0	II	Male	75	>65
TCGA-DM	COAD	743	1	484	1	NA	NA	743	NA	T3	N0	M0	II	Male	65	<=65
TCGA-AA-	COAD	731	0	394	1	NA	NA	731	0	T3	N2	M1	IV	Male	74	>65
TCGA-A6-	COAD	1331	1	535	1	NA	NA	1331	1	T3	N2	M1	IV	Male	85	>65
TCGA-AA-	COAD	31	0	31	0	NA	NA	31	0	T2	N0	M0	I	Female	81	>65
TCGA-AD-	COAD	331	1	325	1	NA	NA	331	1	T4	N2	NA	NA	Male	58	<=65
TCGA-CK-	COAD	4502	0	4502	0	4502	0	4502	0	T3	N1	M0	III	Female	45	<=65
TCGA-AA-	COAD	1551	0	1216	1	NA	NA	1551	0	T3	N1	M1	IV	Male	72	>65
TCGA-AA-	COAD	61	0	61	0	NA	NA	61	0	T3	N0	M0	II	Female	89	>65
TCGA-AA-	COAD	1095	1	396	1	NA	NA	1095	1	T3	N2	M1	IV	Female	47	<=65
TCGA-AD-	COAD	472	1	343	1	343	1	472	1	T3	N1	M0	III	Male	73	>65
TCGA-AA-	COAD	396	1	122	1	122	1	396	0	T3	N2	M0	III	Male	85	>65
TCGA-AA-	COAD	1005	0	1005	0	1005	0	1005	0	T3	N1	M0	III	Female	63	<=65
TCGA-A6-	COAD	424	1	381	1	NA	NA	424	1	T4	N1	M1	IV	Male	70	>65
TCGA-AA-	COAD	365	0	365	0	365	0	365	0	T3	N0	M0	II	Male	63	<=65
TCGA-AM	COAD	28	0	28	0	NA	NA	28	0	T3	N0	M0	II	Female	68	>65
TCGA-4N-	COAD	146	0	146	0	NA	NA	146	0	T4	N1	M0	III	Male	67	>65
TCGA-G4-	COAD	2268	0	2268	0	NA	NA	2268	0	T3	N2	M0	III	Male	69	>65
TCGA-AA-	COAD	397	0	397	0	NA	NA	397	0	T4	N1	M1	IV	Male	69	>65
TCGA-NH	COAD	588	0	588	0	588	0	588	0	T3	N0	NA	II	Female	90	>65
TCGA-AA-	COAD	242	1	242	1	NA	NA	242	1	T3	N2	M1	IV	Male	84	>65
TCGA-AA-	COAD	424	0	424	0	424	0	424	0	T3	N0	M0	II	Male	76	>65
TCGA-AA-	COAD	670	1	670	1	NA	NA	670	1	T3	N0	M0	II	Female	84	>65
TCGA-DM	COAD	179	1	179	0	NA	NA	179	NA	T3	N2	M1	IV	Female	71	>65
TCGA-AA-	COAD	579	1	153	1	NA	NA	579	1	T3	N0	M0	II	Male	77	>65
TCGA-AA-	COAD	30	0	30	0	NA	NA	30	0	T2	N0	M0	I	Male	84	>65
TCGA-AA-	COAD	822	1	822	1	NA	NA	822	1	T4	N2	M1	IV	Male	76	>65
TCGA-G4-	COAD	254	0	254	0	NA	NA	254	0	T3	N0	M0	II	Female	70	>65
TCGA-AA-	COAD	153	1	153	1	NA	NA	153	1	T3	N0	M1	IV	Female	70	>65
TCGA-G4-	COAD	796	0	796	0	NA	NA	796	0	T3	N0	M0	II	Female	58	<=65
TCGA-AA-	COAD	485	0	485	0	485	0	485	0	T3	N0	M0	II	Female	63	<=65
TCGA-AA-	COAD	276	0	276	0	276	0	276	0	T3	N1	M0	III	Male	63	<=65
TCGA-A6-	COAD	1001	0	1001	0	1001	0	1001	0	T2	N0	M0	I	Male	74	>65
TCGA-AA-	COAD	31	0	31	0	NA	NA	31	0	T3	N1	M0	III	Female	47	<=65
TCGA-AZ-	COAD	172	1	172	1	NA	NA	172	1	T4	N1	M1	IV	Female	71	>65
TCGA-D5-	COAD	346	0	346	0	NA	NA	346	0	T3	N0	M0	II	Male	69	>65
TCGA-G4-	COAD	715	1	291	1	NA	NA	715	1	T4	N1	NA	III	Male	90	>65
TCGA-AD-	COAD	682	1	468	1	468	1	682	1	T3	N0	NA	NA	Male	78	>65
TCGA-AA-	COAD	791	0	791	0	791	0	791	0	T2	N0	M0	I	Female	54	<=65
TCGA-AA-	COAD	1158	1	762	1	NA	NA	1158	1	T3	N1	M0	III	Female	80	>65
TCGA-AA-	COAD	608	0	608	0	NA	NA	608	0	T3	N2	M0	III	Male	82	>65
TCGA-AA-	COAD	153	1	153	1	NA	NA	153	1	T3	N1	M1	IV	Female	75	>65
TCGA-A6-	COAD	1068	0	1068	0	1068	0	1068	0	T3	N0	M0	II	Female	72	>65
TCGA-A6-	COAD	1305	1	1305	0	1305	0	1305	0	T4	N0	M0	II	Female	75	>65
TCGA-DM	COAD	4000	0	4000	0	NA	NA	4000	0	T3	N2	M0	III	Male	82	>65
TCGA-AA-	COAD	549	1	549	1	NA	NA	549	1	T3	N2	M0	III	Female	90	>65
TCGA-AA-	COAD	578	0	578	0	578	0	578	0	T2	N0	M0	I	Male	70	>65
TCGA-AA-	COAD	28	0	28	0	NA	NA	28	0	T3	N1	M0	III	Female	90	>65
TCGA-4T-	COAD	385	0	385	0	385	0	385	0	T3	N0	NA	II	Female	42	<=65
TCGA-AA-	COAD	1065	0	608	1	NA	NA	1065	0	T3	N2	M0	III	Male	47	<=65
TCGA-CM	COAD	609	0	609	0	609	0	609	0	T2	N0	M0	I	Male	69	>65
TCGA-G4-	COAD	1359	0	1359	0	NA	NA	1359	0	T2	N0	M0	NA	Male	71	>65
TCGA-AZ-	COAD	3324	0	3324	0	3324	0	3324	0	T3	N1	M0	III	Female	47	<=65
TCGA-AA-	COAD	1157	0	1157	0	1157	0	1157	0	T3	N0	M0	II	Male	66	>65
TCGA-AA-	COAD	61	1	61	1	NA	NA	61	1	T3	N2	M0	III	Male	68	>65
TCGA-AA-	COAD	31	0	31	0	NA	NA	31	0	T3	N0	M1	IV	Male	55	<=65
TCGA-CM	COAD	822	0	822	0	822	0	822	0	T3	N1	M0	III	Male	62	<=65
TCGA-AA-	COAD	31	0	31	0	NA	NA	31	0	T2	N0	M0	I	Female	81	>65
TCGA-G4-	COAD	1199	0	1199	0	NA	NA	1199	0	T3	N1	NA	III	Male	80	>65
TCGA-AA-	COAD	456	0	456	0	NA	NA	456	0	T1	N0	M0	I	Male	80	>65
TCGA-AA-	COAD	518	0	518	0	518	0	518	0	T3	N1	M0	III	Male	50	<=65
TCGA-AA-	COAD	245	0	245	0	NA	NA	245	0	T3	N1	M0	III	Male	90	>65
TCGA-AA-	COAD	1186	0	1186	0	1186	0	1186	0	T3	N0	M0	II	Male	67	>65
TCGA-D5-	COAD	378	0	378	0	NA	NA	378	0	T2	N0	M0	I	Male	57	<=65
TCGA-AA-	COAD	730	0	730	0	NA	NA	730	0	T4	N1	M1	IV	Female	84	>65
TCGA-AA-	COAD	549	0	549	0	NA	NA	549	0	T3	N0	M0	II	Male	79	>65
TCGA-AA-	COAD	30	0	30	0	NA	NA	30	0	T2	N0	M0	I	Female	64	<=65
TCGA-AA-	COAD	1035	0	1035	0	1035	0	1035	0	T3	N0	M0	II	Female	75	>65
TCGA-DM	COAD	383	1	383	0	NA	NA	383	NA	T3	N1	NA	NA	Female	50	<=65
TCGA-A6-	COAD	1054	0	1054	0	NA	NA	1054	0	T3	N2	M0	III	Female	53	<=65
TCGA-AA-	COAD	791	0	791	1	NA	NA	791	0	T2	N1	M0	III	Female	80	>65
TCGA-G4-	COAD	1089	0	1089	0	NA	NA	1089	0	T3	N0	M0	II	Female	73	>65
TCGA-AA-	COAD	457	0	457	0	NA	NA	457	0	T3	N2	M1	IV	Male	59	<=65
TCGA-A6-	COAD	1366	0	1366	0	1366	0	1366	0	T3	N0	NA	II	Female	73	>65
TCGA-AA-	COAD	457	0	457	0	NA	NA	457	0	T2	N1	M0	III	Male	75	>65
TCGA-AD-	COAD	746	0	746	0	746	0	746	0	T1	N0	NA	NA	Male	65	<=65
TCGA-AA-	COAD	457	0	457	0	457	0	457	0	T3	N0	M0	II	Female	57	<=65
TCGA-CA-	COAD	306	1	207	1	207	1	306	1	T3	N0	M0	II	Male	46	<=65
TCGA-F4-	COAD	188	1	188	0	NA	NA	188	0	T3	N0	M0	II	Female	78	>65
TCGA-AA-	COAD	1064	0	1064	0	1064	0	1064	0	T4	N0	M0	II	Female	46	<=65
TCGA-AA-	COAD	912	0	912	0	NA	NA	912	0	T3	N2	M1	IV	Female	71	>65
TCGA-AA-	COAD	1188	1	1188	0	1188	0	1188	0	T3	N0	M0	II	Male	81	>65
TCGA-AA-	COAD	306	1	306	1	NA	NA	306	1	T3	N2	M0	III	Female	82	>65
TCGA-D5-	COAD	1316	0	1316	0	1316	0	1316	0	T3	N0	M0	II	Female	62	<=65
TCGA-A6-	COAD	504	1	300	1	NA	NA	504	1	T4	N0	M1	IV	Female	57	<=65
TCGA-AA-	COAD	1127	0	1127	0	1127	0	1127	0	T3	N0	M0	II	Male	69	>65
TCGA-F4-	COAD	338	1	288	1	NA	NA	338	1	T4	N2	M0	III	Female	41	<=65
TCGA-AA-	COAD	1035	0	1035	0	NA	NA	1035	0	T3	N2	M0	III	Male	66	>65
TCGA-AA-	COAD	31	0	31	0	NA	NA	31	0	T2	N0	M0	I	Male	59	<=65
TCGA-AZ-	COAD	1977	0	411	1	NA	NA	1977	0	T3	N2	M1	IV	Male	49	<=65
TCGA-DM	COAD	228	1	228	0	NA	NA	228	NA	T3	N2	M0	III	Female	71	>65
TCGA-AA-	COAD	1006	0	1006	0	NA	NA	1006	0	T3	N0	M0	II	Male	74	>65
TCGA-AA-	COAD	396	1	396	1	NA	NA	396	1	T3	N2	M0	III	Female	74	>65
TCGA-AA-	COAD	1157	0	1157	0	NA	NA	1157	0	T3	N0	M0	II	Male	80	>65
TCGA-G4-	COAD	1631	0	859	1	NA	NA	1631	0	T4	N0	M0	II	Female	66	>65
TCGA-A6-	COAD	962	0	962	0	962	0	962	0	T3	N1	M0	III	Male	65	<=65
TCGA-AA-	COAD	30	0	30	0	NA	NA	30	0	T3	N0	M0	II	Male	59	<=65
TCGA-AA-	COAD	182	0	182	0	182	0	182	0	T3	N1	M0	III	Female	77	>65
TCGA-CM	COAD	244	0	244	0	244	0	244	0	T1	N1	M0	III	Female	34	<=65
TCGA-AZ-	COAD	357	1	357	1	NA	NA	357	1	T4	N2	M1	IV	Male	81	>65
TCGA-AA-	COAD	1581	0	1581	0	1581	0	1581	0	T3	N0	M0	II	Male	67	>65
TCGA-AA-	COAD	1278	0	1278	0	1278	0	1278	0	T4	N1	M0	III	Female	66	>65
TCGA-AA-	COAD	28	0	28	0	NA	NA	28	0	T3	N0	M0	II	Male	82	>65
TCGA-DM	COAD	3974	0	3974	0	NA	NA	3974	0	T3	N0	M0	II	Female	79	>65
TCGA-NH	COAD	553	0	553	0	553	0	553	0	T3	N0	NA	II	Female	68	>65
TCGA-AZ-	COAD	368	1	368	1	NA	NA	368	1	T4	N1	M1	IV	Male	64	<=65
TCGA-D5-	COAD	408	0	408	0	NA	NA	408	0	T3	N1	M1	IV	Female	49	<=65
TCGA-D5-	COAD	491	0	491	0	NA	NA	491	0	T2	N0	M0	I	Male	66	>65
TCGA-AZ-	COAD	2683	0	2683	0	2683	0	2683	0	T1	N0	M0	I	Female	51	<=65
TCGA-AA-	COAD	426	1	306	1	NA	NA	426	1	T4	N2	M1	IV	Male	50	<=65
TCGA-AA-	COAD	426	0	426	0	426	0	426	0	T2	N0	M0	I	Male	68	>65
TCGA-AA-	COAD	61	1	61	1	NA	NA	61	1	T3	N2	M1	IV	Male	66	>65
TCGA-AA-	COAD	945	0	945	0	945	0	945	0	T2	N0	M0	I	Male	67	>65
TCGA-AA-	COAD	1216	0	1216	0	NA	NA	1216	0	T3	N1	M1	IV	Female	68	>65
TCGA-AD-	COAD	500	0	500	0	500	0	500	0	T2	N0	NA	I	Male	51	<=65
TCGA-AA-	COAD	153	1	153	1	NA	NA	153	1	T3	N2	M1	IV	Male	58	<=65
TCGA-A6-	COAD	1133	0	948	1	NA	NA	1133	0	T3	N0	M0	II	Female	48	<=65
TCGA-AA-	COAD	669	0	669	0	669	0	669	0	T3	N0	M0	II	Male	70	>65
TCGA-AA-	COAD	1126	0	396	1	396	1	1126	0	T2	N1	M0	III	Male	51	<=65
TCGA-AA-	COAD	92	1	92	0	NA	NA	92	0	T3	N0	M0	II	Female	90	>65
TCGA-5M-	COAD	49	1	49	1	NA	NA	49	1	T3	N0	M1	IV	Male	74	>65
TCGA-AA-	COAD	395	0	395	0	395	0	395	0	T3	N2	M0	III	Male	69	>65
TCGA-AD-	COAD	763	0	763	0	763	0	763	0	T3	N1	M0	III	Male	84	>65
TCGA-AA-	COAD	61	1	61	1	NA	NA	61	1	T3	N2	M1	IV	Female	74	>65
TCGA-AA-	COAD	975	0	975	0	NA	NA	975	0	T2	N0	M0	I	Male	72	>65
TCGA-NH	COAD	476	0	476	0	NA	NA	476	0	T3	N2	NA	III	Female	71	>65
TCGA-AA-	COAD	334	1	183	1	NA	NA	334	1	T3	N2	M0	III	Female	47	<=65
TCGA-AA-	COAD	761	0	761	0	761	0	761	0	T3	N0	M0	II	Female	41	<=65
TCGA-CM	COAD	701	0	701	0	701	0	701	0	T3	N0	M0	II	Male	69	>65
TCGA-A6-	COAD	1126	1	1126	0	1126	0	1126	0	T3	N0	M0	II	Female	81	>65
TCGA-RU-	COAD	1177	0	256	1	NA	NA	1177	0	T3	N2	NA	III	Male	51	<=65
TCGA-AY-	COAD	652	0	652	0	652	0	652	0	T3	N0	NA	II	Male	60	<=65
TCGA-D5-	COAD	354	0	354	0	NA	NA	354	0	T3	N0	M0	II	Male	80	>65
TCGA-G4-	COAD	1674	0	1674	0	NA	NA	1674	0	T3	N1	M0	III	Female	37	<=65
TCGA-A6-	COAD	1387	0	1150	1	1150	1	1387	0	T3	N0	M0	II	Female	73	>65
TCGA-CM	COAD	337	0	337	0	NA	NA	337	0	T2	N0	M0	I	Male	82	>65
TCGA-AA-	COAD	1036	0	1036	0	1036	0	1036	0	T2	N0	M0	I	Male	80	>65
TCGA-AA-	COAD	426	0	426	0	426	0	426	0	T2	N0	M0	I	Female	36	<=65
TCGA-NH	COAD	511	1	511	1	NA	NA	511	1	T4	N2	M1	IV	Male	79	>65
TCGA-AY-	COAD	496	1	186	1	NA	NA	496	1	T3	N2	M0	III	Female	50	<=65
TCGA-NH	COAD	334	1	276	1	NA	NA	334	1	T4	N0	M1	IV	Male	42	<=65
TCGA-AA-	COAD	90	1	90	1	NA	NA	90	1	T3	N1	M1	IV	Female	82	>65
TCGA-CM	COAD	1126	0	1126	0	1126	0	1126	0	T2	N0	M0	I	Male	61	<=65
TCGA-AZ-	COAD	2310	0	2310	0	2310	0	2310	0	T1	N0	M0	I	Female	51	<=65
TCGA-G4-	COAD	2600	0	2176	1	2176	1	2600	0	T3	N1	M0	III	Female	40	<=65
TCGA-AA-	COAD	974	0	974	0	NA	NA	974	0	T3	N1	M0	III	Male	72	>65
TCGA-G4-	COAD	1095	0	592	1	NA	NA	1095	0	T3	N2	NA	III	Female	51	<=65
TCGA-CM	COAD	792	0	792	0	792	0	792	0	T4	N1	M0	III	Male	53	<=65
TCGA-AA-	COAD	61	1	61	1	NA	NA	61	1	T3	N1	M0	III	Male	68	>65
TCGA-CM	COAD	761	0	761	0	NA	NA	761	0	T4	N1	M1	IV	Male	47	<=65
TCGA-G4-	COAD	1093	0	38	1	NA	NA	1093	0	T3	N2	M1	IV	Female	76	>65
TCGA-AA-	COAD	122	1	122	1	NA	NA	122	1	T4	N0	M0	II	Male	75	>65
TCGA-AA-	COAD	1126	0	1126	1	NA	NA	1126	0	T3	N0	M0	II	Male	68	>65
TCGA-AA-	COAD	822	0	822	0	NA	NA	822	0	T3	N2	M0	III	Female	83	>65
TCGA-AZ-	COAD	156	1	156	1	NA	NA	156	1	T3	N2	M1	IV	Female	82	>65
TCGA-AA-	COAD	1430	0	1430	0	1430	0	1430	0	T2	N1	M0	III	Female	60	<=65
TCGA-AA-	COAD	943	0	943	0	943	0	943	0	T3	N2	M0	III	Female	74	>65
TCGA-A6-	COAD	662	0	662	0	NA	NA	662	0	T3	N1	NA	III	Female	55	<=65
TCGA-AA-	COAD	1246	0	1246	0	1246	0	1246	0	T2	N0	M0	I	Female	65	<=65
TCGA-AY-	COAD	588	0	588	0	588	0	588	0	T2	N0	M0	I	Female	54	<=65
TCGA-CA-	COAD	379	0	379	0	379	0	379	0	T3	N0	M0	II	Female	54	<=65
TCGA-AA-	COAD	730	0	730	0	730	0	730	0	T2	N0	M0	I	Female	61	<=65
TCGA-AD-	COAD	805	0	648	1	648	1	805	0	T4	N2	M0	III	Male	62	<=65
TCGA-AZ-	COAD	97	1	97	1	NA	NA	97	1	T4	N2	M1	IV	Male	69	>65
TCGA-D5-	COAD	287	0	287	0	NA	NA	287	0	T3	N0	M0	II	Male	34	<=65
TCGA-5M-	COAD	290	1	219	1	NA	NA	290	1	T4	N2	M1	IV	Female	40	<=65
TCGA-AA-	COAD	184	0	184	0	NA	NA	184	0	T4	N2	M1	IV	Female	80	>65
TCGA-AA-	COAD	31	0	31	0	NA	NA	31	0	T3	N0	M0	II	Female	81	>65
TCGA-AA-	COAD	549	0	549	0	NA	NA	549	0	T1	N0	M0	I	Female	78	>65
TCGA-AA-	COAD	1186	0	821	1	821	1	1186	0	T3	N0	M0	II	Male	60	<=65
TCGA-AA-	COAD	306	1	306	1	NA	NA	306	1	T3	N2	M0	III	Female	84	>65
TCGA-AA-	COAD	1522	0	1522	0	1522	0	1522	0	T3	N0	M0	II	Female	53	<=65
TCGA-AA-	COAD	1765	0	1765	0	NA	NA	1765	0	T2	N0	M0	I	Male	77	>65
TCGA-AA-	COAD	821	0	821	0	821	0	821	0	T3	N0	M0	II	Female	80	>65
TCGA-AA-	COAD	639	0	639	0	639	0	639	0	T2	N0	M0	I	Male	69	>65
TCGA-AA-	COAD	2587	0	2587	0	NA	NA	2587	0	T3	N0	M0	II	Male	77	>65
TCGA-A6-	COAD	751	0	751	0	NA	NA	751	0	T3	N0	M1	IV	Male	59	<=65
TCGA-AA-	COAD	913	0	913	0	913	0	913	0	T3	N0	M0	II	Male	65	<=65
TCGA-F4-	COAD	972	1	313	1	313	1	972	1	T3	N1	M0	III	Female	51	<=65
TCGA-D5-	COAD	543	0	493	1	NA	NA	543	0	T3	N0	M0	II	Male	73	>65
TCGA-AA-	COAD	822	0	822	0	822	0	822	0	T3	N1	M0	III	Male	69	>65
TCGA-AA-	COAD	31	0	31	0	NA	NA	31	0	T3	N0	M0	II	Female	48	<=65
TCGA-F4-	COAD	403	1	403	1	NA	NA	403	1	T3	N1	M1	IV	Female	52	<=65
TCGA-AA-	COAD	2375	0	2375	0	2375	0	2375	0	T3	N0	M0	II	Female	51	<=65
TCGA-A6-	COAD	987	0	987	0	987	0	987	0	T3	N1	M0	III	Female	57	<=65
TCGA-AA-	COAD	1946	0	1946	0	1946	0	1946	0	T3	N0	M0	II	Male	70	>65
TCGA-AA-	COAD	1034	0	1034	0	1034	0	1034	0	T3	N2	M0	III	Female	71	>65
TCGA-AA-	COAD	1005	0	1005	0	1005	0	1005	0	T3	N0	M0	II	Female	65	<=65

Table S2. The clinical information of GSE17538 dataset

Samples	OS.time	OS	DFS	DFS.time	DSS	DSS.time	Age	Gender	Stage	Grade
GSM437096	3576.3	0	0	3576.3	0	3576.3	56	Female	1	Well
GSM437100	3324.6	0	0	3276.3	0	3324.6	63	Male	1	Moderately
GSM437102	2523.9	0	0	2523.9	0	2523.9	66	Male	1	Moderately
GSM437111	1503	0	0	1503	0	1503	71	Male	1	Moderately
GSM437113	908.1	0	0	592.5	0	908.1	58	Male	1	Moderately
GSM437118	1659.9	0	0	1659.9	0	1659.9	77	Female	2	Poorly
GSM437119	4045.8	1	1	2809.8	1	4045.8	41	Male	2	Moderately
GSM437124	863.7	1	1	580.8	1	863.7	80	Male	2	Moderately
GSM437125	804.6	1	0	804.6	0	804.6	66	Male	2	Moderately
GSM437130	1011.9	1	1	423.9	1	1011.9	54	Female	2	Moderately
GSM437133	1119.3	1	1	1119.3	1	1119.3	75	Female	2	Well
GSM437134	2592.9	0	0	2592.9	0	2592.9	74	Female	2	Moderately
GSM437135	1677.6	0	0	1677.6	0	1677.6	74	Female	2	Moderately
GSM437136	2119.5	0	0	2119.5	0	2119.5	83	Female	2	Moderately
GSM437139	2519.7	0	1	671.4	0	2519.7	65	Female	2	Moderately
GSM437144	1119.3	1	0	1119.3	0	1119.3	71	Male	2	Moderately
GSM437145	660.6	1	1	660.6	1	660.6	78	Female	2	Moderately
GSM437152	1753.5	0	0	1401.3	0	1753.5	48	Female	2	Moderately
GSM437153	1379.7	0	0	1379.7	0	1379.7	68	Male	2	Moderately
GSM437157	1177.5	0	0	1177.5	0	1177.5	59	Female	2	Poorly
GSM437159	757.2	0	0	757.2	0	757.2	71	Male	2	Moderately
GSM437163	1103.4	0	0	876.6	0	1103.4	64	Female	2	Moderately
GSM437164	1245.6	0	0	858.9	0	1245.6	84	Female	2	Moderately
GSM437174	727.8	1	1	357	1	727.8	46	Male	3	Moderately
GSM437175	3155.1	0	0	3155.1	0	3155.1	39	Male	3	Moderately
GSM437180	825.3	1	1	638.1	1	825.3	53	Male	3	Well
GSM437181	1377.6	1	1	482.1	1	1377.6	53	Female	3	Moderately
GSM437185	2985.3	0	0	2985.3	0	2985.3	48	Female	3	Well
GSM437187	2298	1	1	827.4	1	2298	58	Male	3	Poorly
GSM437188	300.6	1	1	286.8	1	300.6	75	Male	3	Moderately
GSM437189	1243.5	1	1	700.2	1	1243.5	63	Male	3	Moderately
GSM437190	2887.8	0	0	2688.6	0	2887.8	46	Male	3	Moderately
GSM437191	2568.3	0	0	2568.3	0	2568.3	71	Female	3	Moderately
GSM437197	170.4	1	1	99.6	1	170.4	83	Female	3	Moderately
GSM437199	2222.1	0	1	745.5	0	2222.1	66	Male	3	Moderately
GSM437200	1522.8	1	1	1231.8	1	1522.8	69	Male	3	Moderately
GSM437201	2034.6	1	1	1107.6	1	2034.6	64	Female	3	Moderately
GSM437203	1517.7	0	0	1517.7	0	1517.7	67	Female	3	Poorly
GSM437204	1037.4	1	NA	0	1	1037.4	57	Male	3	Moderately
GSM437205	1161.6	1	0	1161.6	0	1161.6	73	Female	3	Moderately
GSM437209	1780.2	0	0	1780.2	0	1780.2	69	Male	3	Poorly
GSM437212	787.8	1	0	787.8	0	787.8	78	Female	3	Poorly
GSM437214	961.5	1	1	631.2	1	961.5	46	Male	3	Moderately
GSM437215	1678.5	0	0	1340.1	0	1678.5	67	Male	3	Moderately
GSM437219	1473.3	0	0	1473.3	0	1473.3	71	Female	3	Moderately
GSM437224	27.6	0	0	27.6	0	27.6	54	Male	3	Moderately
GSM437225	940.8	0	0	940.8	0	940.8	78	Female	3	Moderately
GSM437226	1209	0	0	568.8	0	1209	61	Female	3	Moderately
GSM437227	3499.2	0	1	2575.2	0	3499.2	74	Male	3	Moderately
GSM437229	599.4	0	0	599.4	0	599.4	81	Female	3	Poorly
GSM437230	738.6	0	0	156.6	0	738.6	62	Female	3	Moderately
GSM437234	159.6	1	NA	0	1	159.6	46	Male	4	Moderately
GSM437235	70.8	1	NA	0	1	70.8	69	Male	4	Poorly
GSM437236	171.6	1	NA	0	1	171.6	73	Male	4	Moderately
GSM437239	148.8	1	NA	0	1	148.8	88	Male	4	Moderately
GSM437243	563.1	1	1	318.3	1	563.1	69	Male	4	Moderately
GSM437251	240.6	1	NA	0	1	240.6	63	Male	4	Moderately
GSM437254	1108.5	1	NA	0	0	1108.5	76	Male	4	Poorly
GSM437257	692.1	1	NA	0	1	692.1	42	Female	4	Moderately
GSM437258	595.5	1	NA	0	1	595.5	78	Female	4	Moderately
GSM437262	457.5	1	NA	0	1	457.5	73	Male	4	Moderately
GSM437268	683.4	1	NA	0	1	683.4	68	Male	4	Moderately
GSM437270	1621.479	0	0	1621.479	NA	NA	70	Female	2	NA
GSM437271	1917.37	0	0	1917.37	NA	NA	48	Female	3	Moderately
GSM437272	1388.712	1	0	1388.712	NA	NA	72	Male	2	Moderately
GSM437273	1800.986	0	0	1800.986	NA	NA	61	Female	3	Well
GSM437274	1447.89	1	1	1247.671	NA	NA	56	Male	3	NA
GSM437275	1800.986	0	0	1800.986	NA	NA	57	Female	3	Moderately
GSM437276	1816.767	0	1	1806.904	NA	NA	74	Female	3	Moderately
GSM437277	1712.219	0	0	1712.219	NA	NA	52	Male	3	Moderately
GSM437278	1675.726	0	0	1675.726	NA	NA	70	Female	2	Moderately
GSM437280	1642.192	0	0	1642.192	NA	NA	84	Male	3	Moderately
GSM437281	1738.849	0	0	1738.849	NA	NA	59	Male	2	NA
GSM437283	1793.096	0	0	1793.096	NA	NA	66	Female	1	Moderately
GSM437284	1872	0	0	1872	NA	NA	55	Male	1	NA
GSM437285	1635.288	0	0	1635.288	NA	NA	81	Female	1	Moderately
GSM437286	108.4932	1	1	0	NA	NA	23	Female	4	NA
GSM437287	1483.397	0	0	54.24658	NA	NA	69	Male	2	NA
GSM437288	1925.26	0	0	88.76712	NA	NA	63	Male	2	NA
GSM437289	1506.082	0	0	14.79452	NA	NA	47	Male	3	NA
GSM437290	925.1507	1	1	881.7534	NA	NA	62	Male	4	NA
GSM437291	1747.726	0	0	1628.384	NA	NA	72	Male	2	NA
GSM437292	1229.918	1	1	1048.438	NA	NA	88	Female	4	NA
GSM437293	244.6027	1	1	0	NA	NA	29	Female	4	NA
GSM437294	1563.288	0	0	1382.795	NA	NA	74	Female	3	NA
GSM437295	156.8219	1	1	0	NA	NA	49	Male	4	NA
GSM437296	3344.548	0	0	1363.068	NA	NA	51	Female	3	NA
GSM437297	234.7397	1	0	234.7397	NA	NA	78	Female	3	Poorly
GSM437298	2058.411	0	0	1918.356	NA	NA	67	Male	2	NA
GSM437299	2041.644	0	0	2041.644	NA	NA	72	Male	2	Moderately
GSM437300	1504.11	0	0	1504.11	NA	NA	61	Male	3	Moderately
GSM437301	2125.479	0	1	541.4795	NA	NA	42	Male	4	Moderately
GSM437302	2229.041	0	1	0	NA	NA	57	Female	4	Moderately
GSM437303	1393.644	0	0	1393.644	NA	NA	51	Male	3	Moderately
GSM437304	2303.014	0	0	2303.014	NA	NA	54	Female	4	Moderately
GSM437305	702.2466	1	1	0	NA	NA	65	Male	4	Moderately
GSM437306	648.9863	1	0	648.9863	NA	NA	75	Male	2	Moderately
GSM437307	993.2055	1	0	993.2055	NA	NA	94	Female	2	Moderately
GSM437308	2075.178	0	1	0	NA	NA	78	Female	4	Moderately
GSM437309	1409.425	0	0	1409.425	NA	NA	63	Female	3	Poorly
GSM437310	1597.808	0	0	159.7808	NA	NA	57	Female	2	NA
GSM437311	1625.425	0	0	1493.26	NA	NA	56	Female	3	NA
GSM437312	1220.055	0	0	1220.055	NA	NA	47	Male	3	Moderately
GSM437313	1222.027	0	0	1222.027	NA	NA	51	Male	2	Moderately
GSM437314	415.2329	1	1	116.3836	NA	NA	80	Female	3	Moderately
GSM437315	2113.644	0	0	2113.644	NA	NA	54	Female	3	Moderately
GSM437317	46.35616	1	1	0	NA	NA	77	Female	4	Poorly
GSM437318	529.6438	1	1	0	NA	NA	56	Male	4	Moderately
GSM437319	379.726	1	1	0	NA	NA	38	Female	4	Moderately
GSM437320	207.1233	1	1	0	NA	NA	64	Female	4	Moderately
GSM437321	975.4521	1	1	0	NA	NA	62	Female	4	Moderately
GSM437322	484.274	1	1	0	NA	NA	60	Female	4	Moderately
GSM437323	12.82192	1	0	12.82192	NA	NA	93	Male	2	Moderately
GSM437324	1242.74	0	0	1242.74	NA	NA	69	Male	2	Moderately
GSM437093	4276.5	0	0	4276.5	0	4276.5	73	Male	1	Moderately
GSM437094	3681.6	0	0	3681.6	0	3681.6	63	Male	1	Well
GSM437095	868.8	1	0	868.8	0	868.8	51	Male	1	Moderately
GSM437097	1785.9	0	0	1785.9	0	1785.9	50	Male	1	Moderately
GSM437098	2064.3	1	1	810.6	1	2064.3	63	Female	1	Moderately
GSM437099	2468.7	0	0	2468.7	0	2468.7	66	Male	1	Well
GSM437101	1976.4	1	0	1976.4	0	1976.4	88	Male	1	Moderately
GSM437103	494.1	1	0	494.1	0	494.1	80	Female	1	Moderately
GSM437104	2692.5	0	0	2508	0	2692.5	62	Male	1	Moderately
GSM437105	2282.1	0	0	2282.1	0	2282.1	82	Male	1	Moderately
GSM437106	2034.6	0	0	2034.6	0	2034.6	78	Female	1	Moderately
GSM437107	1567.2	0	0	1567.2	0	1567.2	76	Male	1	Well
GSM437108	1923	0	0	1923	0	1923	87	Female	1	Moderately
GSM437109	1653.9	0	0	1653.9	0	1653.9	74	Female	1	Moderately
GSM437110	1733.7	0	0	1733.7	0	1733.7	55	Male	1	Moderately
GSM437112	1313.7	0	0	1313.7	0	1313.7	86	Female	1	Moderately
GSM437114	109.2	0	0	109.2	0	109.2	78	Male	1	Moderately
GSM437115	1097.7	0	0	718.8	0	1097.7	57	Male	1	Well
GSM437116	600.6	0	0	600.6	0	600.6	81	Male	1	Moderately
GSM437117	3557.4	0	0	3557.4	0	3557.4	74	Female	2	Moderately
GSM437120	3818.7	0	0	3323.7	0	3818.7	43	Female	2	Moderately
GSM437121	3618.6	0	1	829.2	0	3618.6	51	Female	2	Moderately
GSM437122	3208.2	0	0	3208.2	0	3208.2	70	Male	2	Moderately
GSM437123	351	1	NA	0	1	351	78	Female	2	Moderately
GSM437126	1575	1	0	1575	1	1575	76	Female	2	Moderately
GSM437127	720.9	1	1	271.5	1	720.9	58	Female	2	Poorly
GSM437128	2669.7	0	0	2669.7	0	2669.7	74	Male	2	Moderately
GSM437129	3073.2	0	0	1945.8	0	3073.2	49	Male	2	Moderately
GSM437131	2558.4	0	0	2558.4	0	2558.4	71	Female	2	Moderately
GSM437132	795.9	0	0	795.9	0	795.9	79	Female	2	Moderately
GSM437137	2281.2	0	0	2281.2	0	2281.2	73	Male	2	Moderately
GSM437138	2373.9	0	0	2373.9	0	2373.9	57	Female	2	Well
GSM437140	2407.5	0	0	2407.5	0	2407.5	86	Female	2	Moderately
GSM437141	1218	0	0	865.8	0	1218	89	Female	2	Well
GSM437142	2861.1	0	1	756.3	0	2861.1	56	Female	2	Poorly
GSM437143	931.8	1	1	931.8	1	931.8	86	Female	2	Poorly
GSM437146	1897.5	0	0	1897.5	0	1897.5	76	Male	2	Moderately
GSM437147	1022.7	0	0	725.7	0	1022.7	66	Female	2	Poorly
GSM437148	1564.2	0	0	1564.2	0	1564.2	65	Female	2	Moderately
GSM437149	1981.2	0	0	1788	0	1981.2	74	Female	2	Moderately
GSM437150	1798.8	0	0	1798.8	0	1798.8	67	Male	2	Well
GSM437151	1435.8	0	0	1435.8	0	1435.8	56	Male	2	Moderately
GSM437154	1812.6	0	0	1495.2	0	1812.6	71	Male	2	Moderately
GSM437155	1130.1	0	0	538.5	0	1130.1	38	Female	2	Moderately
GSM437156	471.3	0	0	471.3	0	471.3	73	Male	2	Moderately
GSM437158	1496.1	0	0	1102.5	0	1496.1	76	Female	2	Moderately
GSM437160	1349.1	0	0	1349.1	0	1349.1	58	Male	2	Moderately
GSM437161	1077	0	0	1077	0	1077	60	Male	2	Moderately
GSM437162	799.8	0	0	799.8	0	799.8	71	Male	2	Moderately
GSM437165	666.6	0	NA	0	0	666.6	87	Female	2	Moderately
GSM437166	996	0	0	996	0	996	51	Male	2	Moderately
GSM437167	1190.4	0	0	1010.7	0	1190.4	69	Male	2	Moderately
GSM437168	768.3	0	0	768.3	0	768.3	78	Male	2	Moderately
GSM437169	2519.7	0	1	671.4	0	2519.7	65	Female	2	Moderately
GSM437170	451.5	0	0	451.5	0	451.5	54	Male	2	Moderately
GSM437171	251.4	0	0	67.8	0	251.4	40	Female	2	Well
GSM437172	545.4	0	0	545.4	0	545.4	68	Male	2	Moderately
GSM437173	814.5	1	0	814.5	0	814.5	52	Female	2	Moderately
GSM437176	352.8	1	1	246.3	1	352.8	38	Male	3	Moderately
GSM437177	2113.5	1	1	881.7	1	2113.5	26	Male	3	Moderately
GSM437178	2852.1	0	0	2852.1	0	2852.1	71	Female	3	Moderately
GSM437179	3369.9	0	0	3369.9	0	3369.9	64	Female	3	Moderately
GSM437182	1621.2	1	1	832.2	1	1621.2	92	Female	3	Moderately
GSM437183	3119.4	0	1	441.6	0	3119.4	60	Male	3	Well
GSM437184	183.3	1	1	160.5	1	183.3	74	Male	3	Poorly
GSM437186	613.2	1	0	613.2	0	613.2	63	Male	3	Moderately
GSM437192	2366.1	0	0	1929.9	0	2366.1	63	Male	3	Moderately
GSM437193	667.5	1	0	667.5	0	667.5	87	Female	3	Well
GSM437194	2235.9	0	0	2235.9	0	2235.9	63	Male	3	Moderately
GSM437195	1772.1	1	0	1772.1	0	1772.1	87	Male	3	Moderately
GSM437196	1434	1	0	1434	0	1434	30	Male	3	Poorly
GSM437198	2381.7	0	0	2162.7	0	2381.7	55	Male	3	Well
GSM437202	2125.2	0	0	1947.9	0	2125.2	62	Male	3	Poorly
GSM437206	1647	1	0	1647	0	1647	83	Female	3	Moderately
GSM437207	2037.6	0	0	1966.5	0	2037.6	65	Female	3	Moderately
GSM437208	1753.5	0	0	1753.5	0	1753.5	63	Female	3	Moderately
GSM437210	1636.2	0	0	807.6	0	1636.2	71	Female	3	Moderately
GSM437211	570.9	1	1	257.4	1	570.9	51	Female	3	Poorly
GSM437213	1585.8	0	0	1585.8	0	1585.8	72	Male	3	Poorly
GSM437216	1700.1	0	0	1107.6	0	1700.1	67	Male	3	Poorly
GSM437217	1512.9	0	0	1512.9	0	1512.9	26	Male	3	Moderately
GSM437218	127.2	1	0	127.2	0	127.2	78	Female	3	Moderately
GSM437220	1487.1	0	0	1142.1	0	1487.1	60	Female	3	Moderately
GSM437221	1514.7	0	0	1342.2	0	1514.7	46	Male	3	Moderately
GSM437222	1891.5	0	1	1025.7	0	1891.5	41	Male	3	Moderately
GSM437223	1477.2	1	1	723.9	1	1477.2	75	Female	3	Moderately
GSM437228	957.6	0	0	957.6	0	957.6	79	Female	3	Moderately
GSM437231	556.2	1	NA	0	1	556.2	64	Male	4	Moderately
GSM437232	3922.5	0	0	3519	0	3922.5	72	Female	4	Moderately
GSM437233	528.6	1	NA	0	1	528.6	59	Female	4	Well
GSM437237	458.4	1	NA	0	1	458.4	66	Male	4	Moderately
GSM437238	2852.1	0	0	2478.3	0	2852.1	54	Male	4	Moderately
GSM437240	866.7	1	NA	0	1	866.7	58	Male	4	Moderately
GSM437241	97.5	1	NA	0	1	97.5	82	Female	4	Poorly
GSM437242	27.6	1	NA	0	1	27.6	58	Female	4	Poorly
GSM437244	1731.9	1	NA	0	1	1731.9	51	Male	4	Moderately
GSM437245	431.7	1	NA	0	1	431.7	70	Female	4	Moderately
GSM437246	153.6	1	NA	0	1	153.6	82	Female	4	Moderately
GSM437247	149.7	1	NA	0	1	149.7	60	Male	4	Poorly
GSM437248	995.1	1	1	746.4	1	995.1	52	Male	4	Poorly
GSM437249	1515.9	1	0	1515.9	0	1515.9	59	Male	4	Moderately
GSM437250	171.6	1	NA	0	1	171.6	56	Male	4	Poorly
GSM437252	1020.6	1	NA	0	1	1020.6	65	Female	4	Moderately
GSM437253	43.2	1	NA	0	1	43.2	54	Male	4	Poorly
GSM437255	1822.5	0	0	1655.7	0	1822.5	58	Female	4	Moderately
GSM437256	1123.2	1	NA	0	0	1123.2	69	Female	4	Moderately
GSM437259	955.5	0	NA	0	0	955.5	51	Male	4	Moderately
GSM437260	258.3	1	NA	0	1	258.3	63	Male	4	Moderately
GSM437261	1268.1	0	1	392.4	0	1268.1	66	Male	4	Moderately
GSM437263	436.8	1	NA	0	1	436.8	62	Male	4	Moderately
GSM437264	298.8	1	0	298.8	0	298.8	82	Female	4	Moderately
GSM437265	268.2	1	0	268.2	1	268.2	76	Male	4	Moderately
GSM437266	980.1	0	1	519.6	0	980.1	78	Female	4	Poorly
GSM437267	62.1	1	NA	0	1	62.1	66	Female	4	Poorly
GSM437269	670.5	0	NA	0	0	670.5	47	Male	4	Moderately
GSM437279	1945.973	0	0	1945.973	NA	NA	55	Male	1	Moderately
GSM437282	1717.151	0	0	1717.151	NA	NA	49	Male	3	NA
GSM437316	1080	1	1	0	NA	NA	70	Female	4	Moderately

Table S3. The clinical information of GSE39582 dataset

Samples	OS.time	OS	RFS.time	RFS	Age	Gender	Stage	T.Stage	N.Stage	M.Stage
GSM97195	270	1	0	1	36.4	Female	4	T3	N2	M1
GSM97196	960	1	30	1	39.2	Female	4	T3	N1	M1
GSM97196	2190	0	2190	0	60.9	Male	3	T4	N2	M0
GSM97196	600	1	510	1	78.2	Male	2	T3	N0	M0
GSM97196	3360	0	2790	1	44.9	Female	2	T4	N0	M0
GSM97197	1380	0	1380	0	24.9	Male	2	T3	N0	M0
GSM97197	1680	0	1680	0	40.7	Female	4	T4	N1	M1
GSM97197	1380	0	1380	0	41.9	Female	2	T3	N0	M0
GSM97197	1470	0	1470	0	40.8	Female	3	T4	N1	M0
GSM97198	1440	0	1440	0	81.8	Male	2	T3	N0	M0
GSM97198	1260	1	1260	0	79.3	Male	3	T4	N2	M0
GSM97198	2040	0	2040	0	66.3	Male	2	T3	N0	M0
GSM97198	2040	0	2040	0	66.7	Male	2	T3	N0	M0
GSM97198	540	1	450	1	77.2	Female	4	T4	N1	M1
GSM97198	720	1	480	1	71.3	Male	1	T2	N0	M0
GSM97199	750	0	750	0	61.8	Female	2	T4	N0	M0
GSM97199	2280	0	2280	0	63	Female	3	T3	N1	M0
GSM97199	2670	0	2670	0	72	Male	3	T3	N2	M0
GSM97200	270	0	270	1	76.4	Female	3	T3	N2	M0
GSM97200	810	1	0	1	64.6	Male	4	T3	N2	M1
GSM97200	2670	0	2670	0	62.9	Female	4	T3	N1	M1
GSM97200	690	1	0	1	67.5	Female	4	T3	N0	M1
GSM97200	60	1	60	0	71	Male	4	T4	N2	M1
GSM97200	2160	1	60	1	67.3	Male	4	T4	N2	M1
GSM97200	300	1	0	1	68.1	Male	4	T3	N2	M1
GSM97201	600	1	540	1	75.3	Female	4	T4	N1	M1
GSM97201	2730	0	2730	0	92.4	Female	2	T3	N0	M0
GSM97201	480	1	420	1	74.5	Female	2	T3	N0	M0
GSM97201	1290	0	1290	0	73	Female	3	T3	N2	M0
GSM97201	750	1	630	1	54	Male	3	T3	N3	M0
GSM97202	1110	1	150	1	58	Female	3	T3	N2	M0
GSM97202	2460	0	2460	0	76	Female	3	T3	N2	M0
GSM97202	1020	1	960	1	75	Male	3	T3	N2	M0
GSM97202	2820	0	2820	0	70	Female	3	T3	N2	M0
GSM97202	2340	0	2340	0	46	Female	3	T3	N2	M0
GSM97202	1080	0	1080	0	58	Male	3	T4	N2	M0
GSM97203	1560	0	1560	0	68	Female	3	T3	N3	M0
GSM97203	1800	0	1800	0	64	Female	2	T3	N0	M0
GSM97203	1500	0	1500	0	77	Male	2	T3	N0	M0
GSM97203	1500	0	1500	0	47	Female	2	T4	N0	M0
GSM97203	30	1	30	0	61	Male	2	T3	N0	M0
GSM97203	1560	1	0	1	65	Male	4	T4	N0	M1
GSM97204	1740	0	630	1	54	Male	2	T3	N0	M0
GSM97204	990	0	990	0	93	Female	2	T3	N0	M0
GSM97205	570	0	570	0	75	Female	2	T3	N0	M0
GSM97205	840	1	0	1	34	Female	4	T3	N0	M1
GSM97205	870	0	870	0	55	Male	2	T2	N0	M0
GSM97205	780	0	780	0	82	Male	2	T3	N0	M0
GSM97205	2790	0	2790	0	31	Female	2	T4	N0	M0
GSM97205	780	1	780	0	70	Male	2	T3	N0	M0
GSM97205	1770	0	1770	0	76	Female	2	T3	N0	M0
GSM97206	1290	1	1290	0	78	Female	2	T4	N0	M0
GSM97206	660	1	0	1	60	Male	4	T4	N0	M1
GSM97206	5490	1	5490	0	83	Female	2	T3	N0	M0
GSM97206	660	1	90	1	44	Male	2	T4	N0	M0
GSM97207	5160	0	5160	0	70	Female	2	T4	N0	M0
GSM97207	930	1	780	1	58	Female	2	T3	N0	M0
GSM97207	5040	0	5040	0	44	Male	2	T3	N0	M0
GSM97208	3960	1	3570	1	75	Male	2	T3	N0	M0
GSM97208	3810	0	3810	0	65	Female	2	T3	N0	M0
GSM97208	2370	0	2370	0	73	Female	2	T4	N0	M0
GSM97208	2610	0	2610	0	68	Female	2	T3	N0	M0
GSM97209	1410	0	1410	0	76	Female	2	T3	N0	M0
GSM97209	1830	0	1830	0	67	Female	2	T3	N0	M0
GSM97209	2010	0	2010	0	50	Male	2	T3	N0	M0
GSM97209	360	1	360	1	64	Male	3	T4	N1	M0
GSM97209	450	1	450	0	66	Male	3	T4	N2	M0
GSM97210	750	0	750	0	71	Female	3	T4	N2	M0
GSM97210	1740	0	840	1	66	Male	3	T3	N2	M0
GSM97210	1230	0	1230	0	72	Female	3	T3	N3	M0
GSM97210	960	0	960	0	53	Male	3	T2	N3	M0
GSM97210	2160	0	270	1	96	Female	3	T3	N1	M0
GSM97210	1800	1	1800	0	76	Male	3	T3	N1	M0
GSM97210	1080	0	1080	0	96	Male	3	T3	N1	M0
GSM97211	660	0	390	1	63	Male	3	T3	N2	M0
GSM97211	960	1	960	0	64	Female	3	T3	N2	M0
GSM97211	810	0	540	1	63	Female	3	T3	N1	M0
GSM97211	1380	0	1380	0	73	Male	3	T3	N1	M0
GSM97211	2250	1	330	1	59	Female	3	T3	N3	M0
GSM97211	870	1	60	1	79	Female	3	T3	N2	M0
GSM97211	900	0	570	1	64	Male	3	T3	N2	M0
GSM97212	2130	0	2130	0	53	Male	2	T3	N0	M0
GSM97212	2250	0	2250	0	49	Female	2	T3	N0	M0
GSM97212	2040	0	420	1	64	Female	2	T3	N0	M0
GSM97212	2550	0	2550	0	66	Female	2	T3	N0	M0
GSM97212	2790	0	2790	0	64	Male	2	T3	N0	M0
GSM97213	1650	0	1650	0	76	Male	2	T4	N0	M0
GSM97213	2580	0	2580	0	78	Female	2	T3	N0	M0
GSM97213	1830	1	1830	0	87	Female	2	T3	N0	M0
GSM97213	1590	1	810	1	64	Male	3	T3	N+	M0
GSM97213	3870	0	270	1	68	Male	2	T3	N0	M0
GSM97214	5160	0	1680	1	64	Male	2	T4	N0	M0
GSM97214	3630	0	3630	0	63	Female	2	T3	N0	M0
GSM97214	1980	0	1980	0	60	Male	3	T3	N2	M0
GSM97214	3810	0	3810	0	59	Male	3	T3	N1	M0
GSM97215	3600	0	3600	0	45	Female	3	T3	N2	M0
GSM97216	2310	0	1170	1	73	Male	2	T3	N0	M0
GSM97217	2190	0	2190	0	67	Female	3	T2	N2	M0
GSM97217	3900	0	3900	0	70	Female	3	T4	N1	M0
GSM97217	150	1	120	1	88	Female	2	T4	N0	M0
GSM97217	2910	0	2910	0	75	Male	3	T3	N1	M0
GSM97217	1290	1	840	1	83	Female	3	T3	N1	M0
GSM97217	1080	1	1050	1	85	Female	3	T4	N2	M0
GSM97217	660	1	600	1	71	Male	2	N/A	N/A	N/A
GSM97217	2010	0	630	1	69	Female	3	N/A	N/A	N/A
GSM97217	540	1	510	1	77	Male	2	N/A	N/A	N/A
GSM97218	720	1	570	1	66	Female	2	N/A	N/A	N/A
GSM97218	1020	1	390	1	69	Male	3	N/A	N/A	N/A
GSM97218	1740	1	690	1	53	Male	2	T4	N0	M0
GSM97218	960	1	330	1	62	Female	3	N/A	N/A	N/A
GSM97218	1170	1	120	1	70	Female	2	N/A	N/A	N/A
GSM97218	1170	0	660	1	58	Female	3	N/A	N/A	N/A
GSM97218	1410	1	420	1	50	Female	3	N/A	N/A	N/A
GSM97218	5760	0	5760	0	63	Male	3	T4	N+	M0
GSM97219	3240	0	3240	0	68	Male	2	T3	N0	M0
GSM97219	4740	0	4740	0	72	Female	2	T3	N0	M0
GSM97219	2190	0	2190	0	51	Female	3	T3	N1	M0
GSM97219	2490	0	2490	0	43	Male	3	T3	N2	M0
GSM97219	2190	0	2190	0	69	Female	2	T3	N0	M0
GSM97219	1500	0	1500	0	78	Female	2	T3	N0	M0
GSM97219	1980	0	1980	0	73	Male	3	T3	N2	M0
GSM97220	1620	0	1620	0	61	Male	2	T3	N0	M0
GSM97220	2010	0	2010	0	78	Male	3	T3	N2	M0
GSM97220	1050	1	210	1	76	Male	2	N/A	N/A	N/A
GSM97220	750	1	0	1	63	Male	4	T4	N2	M1
GSM97220	390	1	0	1	54	Male	4	T3	N2	M1
GSM97221	1380	1	0	1	50	Male	4	T3	N+	M1
GSM97221	30	1	0	1	71	Female	4	T3	N0	M1
GSM97221	1410	0	1410	0	73	Female	3	T4	N2	M0
GSM97221	2820	0	0	1	45	Female	4	T3	N2	M1
GSM97221	2100	0	0	1	73	Male	4	T3	N1	M1
GSM97222	750	1	600	1	74	Male	3	T4	N2	M0
GSM97223	810	0	810	0	55	Male	3	T3	N1	M0
GSM97223	420	0	420	0	54	Male	4	T3	N1	M1
GSM97223	630	0	630	0	69	Male	4	T4	N2	M1
GSM97223	300	1	300	0	79	Male	4	T3	N1	M1
GSM97223	210	0	210	0	57	Female	3	T2	N1	M0
GSM97224	420	0	420	0	67	Female	4	T3	N1	M1
GSM97224	420	0	420	0	51	Female	1	T1	N0	M0
GSM97224	180	0	180	0	79	Male	2	T4	N0	M0
GSM97224	420	0	420	0	58	Male	2	T3	N0	M0
GSM97225	210	1	210	0	57	Male	4	T4	N1	M1
GSM97225	420	0	270	1	77	Female	4	T3	N0	M1
GSM97225	150	0	150	0	81	Female	2	T3	N0	M0
GSM97225	570	0	390	1	63	Female	4	T4	N1	M1
GSM97225	870	0	870	0	72	Female	2	T4	N0	M0
GSM97226	390	0	390	0	70	Male	4	T3	N2	M1
GSM97226	150	0	150	0	88	Female	2	T4	N0	MX
GSM97226	1800	0	1800	0	78	Female	3	T3	N1	M0
GSM97226	600	1	600	0	56	Male	4	T4	N2	M1
GSM97226	2370	0	2370	0	70	Male	1	T2	N0	M0
GSM97227	420	1	390	1	92	Male	3	T4	N1	M0
GSM97227	450	1	450	0	47	Female	4	T3	N1	M1
GSM97227	1710	0	1710	0	91	Female	3	T4	N2	M0
GSM97227	120	1	90	1	76	Male	2	T4	N0	M0
GSM97227	1290	0	1290	0	59	Male	3	T3	N2	M0
GSM97228	1380	1	1380	0	79	Female	2	T4	N0	M0
GSM97228	1530	0	1530	0	79	Female	1	T2	N0	M0
GSM97228	1530	0	1530	0	65	Male	2	T3	N0	M0
GSM97228	1050	1	1050	0	53	Male	4	T4	N1	M1
GSM97229	1500	0	1500	0	NA	Male	1	T2	N0	M0
GSM97229	750	0	750	0	74	Male	2	T3	N0	M0
GSM97229	1830	0	1830	0	68	Male	3	T4	N1	M0
GSM97229	1380	1	1380	0	77	Male	1	T1	N0	M0
GSM97229	1230	1	1230	0	54	Female	4	T4	N2	M1
GSM97230	900	0	900	0	60	Female	2	T3	N0	M0
GSM97230	930	0	930	0	82	Female	3	T4	N2	M0
GSM97230	1680	0	1680	0	70	Male	2	T3	N0	M0
GSM97230	2760	0	2760	0	79	Female	3	T3	N1	M0
GSM97231	3180	0	3180	0	55	Male	2	T3	N0	M0
GSM97231	1380	0	1380	0	86	Female	3	T3	N1	M0
GSM97231	2340	1	2340	0	71	Female	2	T3	N0	M0
GSM97232	1740	1	300	1	64	Male	3	T2	N1	M0
GSM97232	270	0	270	0	52	Male	3	T3	N2	M0
GSM97232	2580	1	1980	1	46	Male	2	T3	N0	M0
GSM97232	2400	0	2400	0	67	Female	3	T3	N1	M0
GSM97232	2460	0	2460	0	72	Male	2	T3	N0	M0
GSM97232	2550	0	2550	0	71	Female	2	T3	N0	M0
GSM97232	2340	0	2340	0	61	Male	2	T3	N0	M0
GSM97233	2310	0	2310	0	79	Female	3	T3	N2	M0
GSM97233	1500	0	1500	0	63	Male	3	T3	N1	M0
GSM97233	1230	1	1230	0	80	Female	3	T3	N1	M0
GSM97234	2520	0	1440	1	75	Male	2	T3	N0	M0
GSM97234	840	0	840	0	78	Male	1	T2	N0	M0
GSM97234	1560	1	1560	0	86	Male	2	T3	N0	M0
GSM97234	2250	0	2250	0	70	Female	2	T3	N0	M0
GSM97234	1260	1	1230	1	83	Male	3	T3	N2	M0
GSM97234	150	1	150	0	79	Female	3	T4	N1	M0
GSM97235	1260	1	1260	0	88	Female	2	T3	N0	M0
GSM97235	900	1	360	1	56	Female	2	T3	N0	M0
GSM97236	4230	0	4230	0	54	Female	3	T3	N1	M0
GSM97236	570	1	540	1	72	Female	3	T4	N2	M0
GSM97236	1410	0	1410	0	59	Female	3	T3	N2	M0
GSM97236	720	1	420	1	50	Male	3	T3	N2	M0
GSM97236	2850	0	2850	0	72	Male	3	T3	N1	M0
GSM97236	3060	1	540	1	58	Male	3	T3	N2	M0
GSM97237	2430	0	2430	0	47	Male	3	T3	N1	M0
GSM97237	2700	0	2700	0	65	Male	3	T1	N1	M0
GSM97237	3510	0	3510	0	46	Female	3	T3	N1	M0
GSM97237	2610	0	2610	0	73	Female	3	T3	N2	M0
GSM97237	2460	0	2460	0	59	Female	3	T3	N1	M0
GSM97237	2580	0	2580	0	78	Male	3	T3	N2	M0
GSM97237	2850	0	2850	0	63	Female	3	T2	N1	M0
GSM97238	450	1	180	1	64	Female	3	T3	N2	M0
GSM97238	1440	1	810	1	63	Male	2	T3	N0	M0
GSM97238	1890	0	1890	0	70	Male	2	T3	N0	M0
GSM97239	240	1	180	1	87	Male	3	T3	N1	M0
GSM97239	1320	1	630	1	75	Female	2	T4	N0	M0
GSM97239	1410	1	150	1	76	Male	2	T3	N0	M0
GSM97239	330	1	180	1	81	Male	3	T3	N1	M0
GSM97240	930	1	0	1	71	Male	2	T3	N0	M1
GSM97240	960	1	960	0	66	Male	2	T3	N0	M0
GSM97240	3120	0	3120	0	72	Female	2	T4	N0	M0
GSM97241	2730	0	2730	0	68	Female	2	T3	N0	M0
GSM97241	240	1	150	1	78	Male	3	T3	N2	M0
GSM97241	600	1	480	1	66	Male	3	T3	N1	M0
GSM97241	240	1	150	1	49	Male	2	T4	N0	M0
GSM97241	4380	0	4380	0	59	Male	2	T3	N0	M0
GSM97241	2100	0	2100	0	64	Male	2	T3	N0	M0
GSM97241	960	1	900	1	85	Female	3	T3	N2	M0
GSM97242	180	1	180	1	83	Male	3	T3	N2	M0
GSM97242	840	0	660	1	66	Male	3	N/A	N/A	N/A
GSM97242	1110	1	390	1	74	Male	2	N/A	N/A	N/A
GSM97242	1200	0	390	1	52	Female	2	N/A	N/A	N/A
GSM97242	3120	0	3120	0	70	Male	2	T3	N0	M0
GSM97242	1770	0	1770	0	72	Female	3	T3	N1	M0
GSM97242	2430	0	2430	0	49	Male	2	T3	N0	M0
GSM97242	1590	0	1590	0	63	Male	2	T3	N0	M0
GSM97242	1650	0	1650	0	62	Male	3	T3	N2	M0
GSM97242	1860	0	1860	0	64	Male	3	T4	N2	M0
GSM97243	1650	0	570	1	71	Male	3	N/A	N/A	N/A
GSM97243	480	1	90	1	85	Male	3	N/A	N/A	N/A
GSM97243	1200	0	420	1	71	Male	3	N/A	N/A	N/A
GSM97243	210	1	0	1	48	Female	4	T3	N2	M1
GSM97243	900	1	0	1	44	Male	4	T4	N1	M1
GSM97244	1560	0	1560	0	50	Male	2	T3	N0	M0
GSM97244	2040	0	0	1	49	Female	4	T4	N2	M1
GSM97244	180	1	0	1	54	Female	4	T3	N2	M1
GSM97244	420	1	150	1	73	Male	3	T4	N2	MX
GSM97244	1050	0	1050	0	48	Male	2	T4	N0	M0
GSM97245	2220	0	2220	0	89	Female	3	T3	N1	M0
GSM97245	2040	0	1020	1	74	Female	3	T3	N1	M0
GSM97245	510	1	510	0	65	Male	2	T3	N0	M0
GSM97246	2880	0	2880	0	70	Female	2	T3	N0	M0
GSM97246	60	1	NA	NA	67	Male	3	T4	N2	M0
GSM97246	2580	0	2580	0	76	Female	1	T2	N0	M0
GSM97246	2160	0	2160	0	54	Male	3	T3	N2	M0
GSM97247	2550	0	2550	0	50	Male	2	T3	N0	M0
GSM97247	1920	0	1920	0	61	Male	3	T4	N1	M0
GSM97247	2430	0	2430	0	75	Male	2	T3	N0	M0
GSM97247	2070	0	2070	0	80	Male	2	T3	N0	M0
GSM97247	90	0	90	0	79	Male	3	T4	N2	M0
GSM97248	1950	1	1950	0	67	Male	3	T3	N2	M0
GSM97248	1260	0	1260	0	90	Female	2	T3	N0	M0
GSM97248	840	1	690	1	80	Female	2	T3	N0	M0
GSM97248	2250	0	2250	0	69	Male	2	T3	N0	M0
GSM97248	990	0	990	0	87	Male	2	T3	N0	M0
GSM97249	1770	0	1770	0	89	Female	2	T3	N0	M0
GSM97249	1710	0	1710	0	43	Female	1	T2	N0	M0
GSM97249	810	1	540	1	69	Female	3	T3	N1	M0
GSM97250	3180	1	3180	0	76	Female	3	T3	N1	M0
GSM97250	3930	0	3930	0	69	Male	2	T3	N0	M0
GSM97250	4380	0	4380	0	42	Female	2	T3	N0	M0
GSM97250	900	1	720	1	80	Male	3	T3	N2	M0
GSM97250	4320	0	4320	0	69	Male	2	T3	N0	M0
GSM97250	4230	0	4230	0	68	Male	2	T3	N0	M0
GSM97251	690	1	120	1	62	Male	3	T4	N2	M0
GSM97251	1050	1	450	1	82	Female	3	T4	N1	M0
GSM97251	420	1	330	1	58	Male	2	T3	N0	M0
GSM97252	2280	1	2280	0	90	Female	2	T3	N0	M0
GSM97252	2970	0	2970	0	53	Female	2	T3	N0	M0
GSM97195	300	1	0	1	34.5	Male	4	T4	N2	M1
GSM97195	1560	0	120	1	36.9	Male	2	T4	N0	M0
GSM97196	2220	0	2220	0	38.2	Female	1	T2	N0	M0
GSM97196	1050	0	1050	0	35.4	Female	3	T2	N2	M0
GSM97196	990	0	990	0	37.5	Female	2	T3	N0	M0
GSM97196	1890	0	1890	0	40.5	Female	3	T3	N1	M0
GSM97196	930	1	930	NA	42.6	Male	2	T3	N0	M0
GSM97196	1890	0	1890	0	30.2	Male	1	T1	N0	M0
GSM97197	2580	0	2580	0	31.7	Female	1	T2	N0	M0
GSM97197	1950	0	1950	0	34.7	Female	4	T4	N2	M1
GSM97197	2250	0	2250	0	40.2	Male	1	T2	N0	M0
GSM97197	1080	0	1080	0	45.2	Female	2	T4	N0	M0
GSM97197	3270	0	3270	0	71.4	Female	1	T2	N0	M0
GSM97197	1590	0	1590	0	66.4	Female	2	T3	N0	M0
GSM97198	1770	0	1770	0	76.9	Male	3	T4	N1	M0
GSM97198	2610	0	2610	0	75.3	Male	3	T3	N1	M0
GSM97198	2430	0	150	1	68	Female	2	T4	N0	M0
GSM97198	210	0	210	0	66.6	Male	0	T0	N0	M0
GSM97199	360	1	360	0	83.1	Male	1	T2	N0	M0
GSM97199	1650	1	1650	0	71.3	Male	2	T3	N0	M0
GSM97199	600	1	600	0	85.6	Male	2	T4	N0	M0
GSM97199	1680	0	1680	0	67.5	Male	2	T3	N0	M0
GSM97199	1290	0	1290	0	80.9	Male	2	T3	N0	M0
GSM97199	690	1	540	1	74.8	Male	3	T3	N1	M0
GSM97199	30	0	30	0	61.5	Male	3	T3	N1	M0
GSM97200	2190	0	2190	0	71.5	Male	3	T2	N1	M0
GSM97200	780	0	750	1	77.8	Male	3	T4	N1	M0
GSM97200	1320	0	0	1	66.1	Male	4	T4	N1	M1
GSM97201	1380	1	300	1	74.5	Male	4	T3	N1	M1
GSM97201	3030	0	3030	0	86.8	Female	4	T3	N1	M1
GSM97201	1290	1	1290	0	89.4	Female	4	T2	N1	M1
GSM97201	870	1	0	1	65.8	Male	4	T3	N0	M1
GSM97201	3510	0	3510	0	68	Female	3	T2	N2	M0
GSM97202	2340	0	270	1	49	Female	3	T3	N2	M0
GSM97202	480	0	180	1	59	Female	3	T3	N1	M0
GSM97202	1290	0	1110	1	62	Male	3	T2	N2	M0
GSM97202	1590	0	1590	0	49	Male	3	T3	N2	M0
GSM97203	1650	0	1650	0	81	Male	2	T3	N0	M0
GSM97203	1500	0	1500	0	45	Female	2	T4	N0	M0
GSM97203	1170	0	1170	0	94	Female	2	T3	N0	M0
GSM97203	990	0	990	0	78	Male	2	T4	N0	M0
GSM97204	1890	0	1890	0	81	Male	2	T3	N0	M0
GSM97204	1650	0	1650	0	78	Male	2	T3	N0	M0
GSM97204	1530	0	1530	0	83	Male	2	T3	N0	M0
GSM97204	810	1	0	1	56	Male	4	T3	N0	M1
GSM97204	1260	0	1260	0	58	Male	2	T4	N0	M0
GSM97204	330	1	0	1	71	Male	4	T3	N0	M1
GSM97204	900	0	0	1	50	Female	4	T4	N0	M1
GSM97204	780	0	780	0	79	Male	2	T3	N0	M0
GSM97205	450	0	450	0	68	Male	2	T2	N0	M0
GSM97205	2760	0	2760	0	78	Female	2	T4	N0	M0
GSM97205	1800	0	1800	0	75	Female	2	T3	N0	M0
GSM97206	2460	0	2460	0	66	Male	2	T3	N0	M0
GSM97206	690	1	0	1	43	Female	2	T4	N0	M0
GSM97206	2100	0	2100	0	54	Male	2	T3	N0	M0
GSM97206	1380	0	1380	0	48	Male	2	T3	N0	M0
GSM97206	1470	0	0	1	69	Male	4	T4	N0	M1
GSM97206	6030	0	6030	0	68	Male	2	T3	N0	M0
GSM97207	2040	1	2040	0	64	Female	2	T4	N0	M0
GSM97207	5490	0	5490	0	68	Female	2	T3	N0	M0
GSM97207	4890	0	4890	0	80	Female	2	T3	N0	M0
GSM97207	2250	1	30	1	70	Female	2	T4	N0	M0
GSM97207	1320	1	1320	0	42	Male	2	T4	N0	M0
GSM97207	4590	0	4590	0	48	Male	2	T3	N0	M0
GSM97207	4350	1	1620	1	63	Male	2	T3	N0	M0
GSM97208	4950	0	1590	1	61	Male	2	T4	N0	M0
GSM97208	780	0	780	0	68	Female	2	T3	N0	M0
GSM97208	3870	0	3870	0	73	Female	2	T3	N0	M0
GSM97208	2910	0	2910	0	56	Male	2	T3	N0	M0
GSM97208	1290	1	1290	0	81	Male	2	T3	N0	M0
GSM97208	960	1	240	1	84	Female	2	T3	N0	M0
GSM97209	2070	0	2070	0	72	Female	2	T3	N0	M0
GSM97209	2100	0	1350	1	67	Male	2	T3	N0	M0
GSM97209	2220	0	2220	0	55	Female	2	T4	N0	M0
GSM97209	450	0	90	1	63	Female	3	T2	N1	M0
GSM97209	450	0	450	0	78	Male	3	T3	N1	M0
GSM97210	1050	0	630	1	56	Male	3	T3	N1	M0
GSM97210	1380	0	1380	0	22	Male	3	T3	N1	M0
GSM97210	3090	0	3090	0	64	Male	3	T4	N1	M0
GSM97211	210	1	120	1	76	Male	3	T4	N2	M0
GSM97211	960	0	330	1	78	Female	3	T3	N1	M0
GSM97211	1110	0	750	1	96	Male	3	T4	N1	M0
GSM97212	1080	0	1080	0	96	Female	3	T3	N1	M0
GSM97212	1050	0	1050	0	96	Male	3	T3	N1	M0
GSM97212	570	1	570	1	97	Female	3	T4	N1	M0
GSM97212	1890	0	1890	0	61	Male	2	T3	N0	M0
GSM97212	330	0	330	0	76	Male	2	T2	N0	M0
GSM97213	420	1	210	1	74	Male	3	T3	N2	M0
GSM97213	390	1	120	1	78	Female	2	T3	N0	M0
GSM97213	3900	0	1830	1	54	Male	3	T3	N1	M0
GSM97213	1590	1	1350	1	77	Male	2	T3	N0	M0
GSM97213	3060	0	1140	1	69	Female	3	T3	N1	M0
GSM97214	2520	0	450	1	58	Male	3	T3	N2	M0
GSM97214	1050	1	60	1	86	Female	3	T3	N2	M0
GSM97214	4620	0	4620	0	57	Male	2	T4	N0	M0
GSM97214	4050	0	4050	0	63	Female	3	T3	N2	M0
GSM97214	4200	0	4200	0	79	Male	2	T3	N0	M0
GSM97214	3960	0	3960	0	64	Male	2	T3	N0	M0
GSM97215	3270	0	3270	0	55	Female	3	T3	N1	M0
GSM97215	2310	0	2310	0	75	Female	3	T3	N1	M0
GSM97215	1680	0	1680	0	70	Female	3	T3	N1	M0
GSM97215	1980	1	1980	0	71	Male	2	T3	N0	M0
GSM97215	1320	1	90	1	49	Female	3	T4	N2	M0
GSM97215	720	1	180	1	42	Male	3	T3	N2	M0
GSM97215	3360	1	3360	0	67	Male	3	T3	N1	M0
GSM97215	3540	0	3540	0	57	Male	3	T3	N1	M0
GSM97215	2790	0	2790	0	67	Male	3	T3	N1	M0
GSM97216	330	1	270	1	91	Female	2	T3	N0	M0
GSM97216	2310	0	2310	0	73	Female	3	T3	N1	M0
GSM97216	1230	0	1230	0	71	Male	2	T4	N0	M0
GSM97216	2070	1	90	1	70	Male	3	T3	N+	M0
GSM97216	2610	1	1080	1	67	Male	2	T4	N0	M0
GSM97216	360	1	90	1	42	Female	3	T3	N+	M0
GSM97216	1350	1	1080	1	47	Male	2	T3	N0	M0
GSM97216	240	1	120	1	76	Male	3	T4	N+	M0
GSM97216	4920	0	4920	0	50	Male	2	T4	N0	M0
GSM97217	780	1	450	1	59	Female	2	T4	N0	M0
GSM97218	1470	0	270	1	62	Male	2	T3	N0	M0
GSM97218	660	0	330	1	58	Female	3	T3	N2	M0
GSM97219	3660	0	3660	0	49	Male	3	T3	N1	M0
GSM97219	3600	0	3600	0	54	Female	2	T3	N0	M0
GSM97219	1710	0	1710	0	52	Female	3	T3	N1	M0
GSM97220	1590	0	1590	0	57	Female	3	T3	N1	M0
GSM97220	1620	0	1620	0	66	Female	3	T3	N2	M0
GSM97220	1320	0	120	1	78	Male	2	N/A	N/A	N/A
GSM97220	1260	0	0	1	74	Male	2	N/A	N/A	N/A
GSM97220	2460	0	1020	1	65	Male	3	T3	N1	M0
GSM97221	1710	0	1710	0	65	Male	3	T3	N1	M0
GSM97221	540	1	0	1	54	Female	4	T3	N1	M1
GSM97221	60	1	0	1	88	Female	4	T3	N2	M1
GSM97221	210	1	0	1	75	Female	4	T4	N3	M1
GSM97221	1590	0	0	1	60	Male	4	T3	N2	M1
GSM97222	30	0	30	0	81	Male	3	T3	N1	M0
GSM97222	1950	0	1950	0	75	Female	2	T3	N0	M0
GSM97222	1710	0	1710	0	69	Female	2	T3	N0	M0
GSM97222	990	1	990	0	78	Male	2	T3	N0	M0
GSM97222	1110	0	1110	0	51	Male	2	T3	N0	M0
GSM97222	600	0	600	0	57	Male	2	T4	N0	M0
GSM97222	600	1	600	0	61	Male	4	T3	N2	M1
GSM97222	30	0	30	0	68	Male	2	T3	N0	M0
GSM97223	450	0	450	0	67	Male	2	T3	N0	M0
GSM97223	360	0	360	0	71	Male	4	T1	N0	M1
GSM97223	750	0	750	0	55	Female	1	T1	N0	M0
GSM97223	570	0	570	0	53	Female	3	T2	N1	M0
GSM97223	420	0	420	0	59	Male	2	T3	N0	M0
GSM97224	510	1	510	0	61	Male	2	T3	N0	M0
GSM97224	510	0	510	0	69	Male	1	T2	N0	M0
GSM97224	60	0	60	0	66	Male	2	T3	N0	M0
GSM97224	420	0	420	0	74	Female	1	T1	N0	M0
GSM97225	360	0	360	0	52	Male	3	T3	N1	M0
GSM97225	480	0	480	0	37	Female	4	T3	N1	M1
GSM97225	480	0	480	0	56	Female	4	T3	N2	M1
GSM97225	630	1	270	1	88	Female	2	T4	N0	M0
GSM97226	210	1	210	0	79	Female	3	T4	N1	M0
GSM97226	2280	0	2280	0	32	Male	3	T2	N1	M0
GSM97226	2190	0	2190	0	46	Male	2	T3	N0	M0
GSM97226	2010	1	2010	0	82	Male	2	T3	N0	M0
GSM97226	1440	0	1440	0	67	Male	3	T2	N1	M0
GSM97227	1950	0	1950	0	75	Female	2	T4	N0	M0
GSM97227	480	1	480	0	84	Male	0	Tis	N0	M0
GSM97227	1530	0	1530	0	88	Female	2	T4	N0	M0
GSM97227	1350	0	1350	0	72	Female	1	T2	N0	M0
GSM97227	1560	0	1560	0	67	Female	3	T3	N1	M0
GSM97228	1680	0	1680	0	64	Male	1	T1	N0	M0
GSM97228	810	1	810	0	63	Female	4	T4	N2	M1
GSM97228	60	1	60	0	71	Male	2	T2	N0	M0
GSM97228	1110	0	1110	0	24	Male	2	T3	N0	M0
GSM97228	1290	0	1290	0	77	Male	2	T3	N0	M0
GSM97228	510	1	510	0	72	Male	4	T4	N1	M1
GSM97229	1170	0	1170	0	70	Female	1	T2	N0	M0
GSM97229	960	0	300	1	46	Male	3	T4	N2	M0
GSM97229	750	0	750	0	62	Female	2	T3	N0	M0
GSM97229	660	0	660	0	77	Female	0	Tis	N0	M0
GSM97229	270	0	270	0	83	Female	2	T3	N0	M0
GSM97230	1230	0	1230	0	75	Male	0	Tis	N0	M0
GSM97230	3150	1	3150	0	74	Male	3	T3	N2	M0
GSM97230	810	1	810	0	79	Male	3	T4	N1	M0
GSM97230	570	0	570	0	80	Female	1	T2	N0	M0
GSM97230	2460	0	2460	0	71	Female	2	T3	N0	M0
GSM97230	2850	0	2850	0	78	Male	2	T3	N0	M0
GSM97231	750	0	750	0	80	Female	3	T3	N1	M0
GSM97231	2910	1	2910	0	71	Male	2	T3	N0	M0
GSM97231	2730	1	2730	0	83	Female	1	T2	N0	M0
GSM97231	2520	0	2520	0	60	Female	1	T1	N0	M0
GSM97231	2490	0	2490	0	77	Female	2	T3	N0	M0
GSM97231	900	1	150	1	85	Male	2	T3	N0	M0
GSM97231	1080	1	1080	0	84	Female	2	T3	N0	M0
GSM97232	2130	0	1590	1	55	Male	3	T3	N1	M0
GSM97232	2490	0	2490	0	60	Male	2	T3	N0	M0
GSM97232	2460	0	2340	1	74	Male	3	T3	N2	M0
GSM97233	2580	0	2580	0	79	Female	2	T3	N0	M0
GSM97233	2250	1	2250	0	79	Male	2	T3	N0	M0
GSM97233	2610	0	2610	0	72	Female	2	T3	N0	M0
GSM97233	2400	0	2400	0	49	Male	2	T3	N0	M0
GSM97233	1380	1	1380	0	73	Male	1	T2	N0	M0
GSM97233	2220	0	2220	0	69	Male	2	T3	N0	M0
GSM97233	2280	0	2280	0	67	Male	3	T3	N1	M0
GSM97234	2070	0	2070	0	89	Female	3	T3	N1	M0
GSM97234	2160	1	2160	0	82	Female	3	T3	N1	M0
GSM97234	210	0	210	0	73	Female	2	T3	N0	M0
GSM97234	1860	0	1860	0	60	Male	2	T3	N0	M0
GSM97235	270	1	270	NA	84	Female	3	T3	N1	M0
GSM97235	720	0	720	0	71	Male	2	T4	N0	M0
GSM97235	4170	0	2220	1	66	Male	3	T3	N2	M0
GSM97235	3990	0	3990	0	46	Female	2	T4	N0	M0
GSM97235	2850	1	690	1	68	Female	3	T3	N2	M0
GSM97235	1020	1	510	1	72	Male	2	T3	N0	M0
GSM97235	1590	1	1590	NA	73	Female	2	T3	N0	M0
GSM97235	2430	0	2430	0	53	Female	2	T3	N0	M0
GSM97236	2940	0	2940	0	50	Male	3	T3	N1	M0
GSM97236	4200	0	4200	0	68	Female	3	T3	N1	M0
GSM97236	3090	0	3090	0	71	Female	3	T3	N1	M0
GSM97236	2760	0	2760	0	70	Female	3	T3	N1	M0
GSM97237	2130	1	960	1	73	Female	3	T3	N1	M0
GSM97237	1500	1	990	1	59	Male	3	T4	N2	M0
GSM97237	2370	0	1110	1	66	Male	3	T3	N1	M0
GSM97238	1140	1	1110	1	58	Male	2	T3	N0	M0
GSM97238	2760	0	2760	0	70	Female	2	T3	N0	M0
GSM97238	3180	1	3180	0	61	Male	2	T3	N0	M0
GSM97238	2700	0	2700	0	77	Female	2	T3	N0	M0
GSM97238	3690	0	720	1	62	Male	2	T3	N0	M0
GSM97238	2490	1	2490	0	75	Female	2	T3	N0	M0
GSM97238	2790	0	2790	0	77	Male	2	T4	N0	M0
GSM97239	1560	1	1290	1	68	Male	2	T3	N0	M0
GSM97239	4410	0	4410	0	64	Male	3	T4	N1	M0
GSM97239	3210	0	3210	0	72	Male	2	T3	N0	M0
GSM97239	3150	0	3150	0	83	Female	2	T3	N0	M0
GSM97239	1920	1	1920	0	65	Male	2	T3	N0	M0
GSM97239	2700	0	2700	0	54	Female	3	T4	N1	M0
GSM97240	330	1	240	1	67.8	Female	4	T4	N2	M1
GSM97240	1680	0	1680	0	70	Female	2	T3	N0	M0
GSM97240	1440	0	1440	0	68	Male	2	T3	N0	M0
GSM97240	2370	1	2370	0	70	Female	2	T4	N0	M0
GSM97240	3180	0	3180	0	66	Female	2	T3	N0	M0
GSM97240	3960	0	3960	0	42	Female	2	T4	N0	M0
GSM97241	3750	0	3750	0	48	Female	3	T1	N1	M0
GSM97241	1590	0	1590	0	52	Female	2	T3	N0	M0
GSM97241	1260	1	240	1	69	Male	3	T3	N1	M0
GSM97243	1590	0	1590	0	43	Female	2	T3	N0	M0
GSM97243	1620	0	1620	0	59	Male	3	T3	N1	M0
GSM97243	1260	1	570	1	70	Male	2	N/A	N/A	N/A
GSM97243	540	1	60	1	81	Male	2	N/A	N/A	N/A
GSM97243	60	1	0	1	65	Male	4	T3	N1	M1
GSM97244	1440	0	1440	0	49	Male	2	T3	N0	M0
GSM97244	930	0	930	0	71	Male	2	T3	N0	M0
GSM97244	1230	1	570	1	51	Female	3	T4	N1	M0
GSM97245	1410	1	570	1	80	Male	2	T3	N0	M0
GSM97245	2910	0	2910	0	58	Male	2	T4	N0	M0
GSM97245	2970	0	2970	0	75	Female	3	T3	N2	M0
GSM97245	3000	0	3000	0	51	Female	3	T3	N1	M0
GSM97245	2250	0	2250	0	62	Male	1	T2	N0	M0
GSM97245	360	0	360	0	80	Male	2	T3	N0	M0
GSM97246	2880	0	2880	0	56	Male	2	T3	N0	M0
GSM97246	2640	0	2640	0	72	Male	1	T2	N0	M0
GSM97246	2520	1	2520	0	73	Male	2	T3	N0	M0
GSM97246	2250	0	2250	0	73	Female	3	T3	N1	M0
GSM97246	720	1	720	0	73	Male	2	T3	N0	M0
GSM97246	2580	0	2580	0	69	Male	1	T2	N0	M0
GSM97247	2400	0	2400	0	58	Male	2	T4	N0	M0
GSM97247	3510	0	1350	1	67	Male	2	T3	N0	M0
GSM97247	3570	0	270	1	49	Female	3	T3	N1	M0
GSM97247	2610	0	2610	0	68	Female	2	T3	N0	M0
GSM97248	2070	0	2070	0	78	Male	2	T3	N0	M0
GSM97248	2280	0	2280	0	68	Male	2	T3	N0	M0
GSM97248	2010	0	2010	0	84	Male	2	T3	N0	M0
GSM97248	1470	0	1470	0	65	Male	2	T3	N0	M0
GSM97248	2220	0	2220	0	77	Female	2	T3	N0	M0
GSM97249	2220	0	2220	0	67	Male	2	T3	N0	M0
GSM97249	2100	0	2100	0	74	Male	2	T3	N0	M0
GSM97249	2040	0	2040	0	69	Male	1	T2	N0	M0
GSM97249	4260	0	4260	0	65	Female	1	T2	N0	M0
GSM97249	30	0	30	0	77	Female	3	T2	N1	M0
GSM97249	1770	1	570	1	50	Female	3	T3	N1	M0
GSM97249	2160	0	2160	0	82	Male	3	T4	N2	M0
GSM97250	1260	0	1260	0	83	Female	3	T3	N1	M0
GSM97250	4020	0	4020	0	69	Female	1	T1	N0	M0
GSM97250	420	0	420	0	73	Female	1	T2	N0	M0
GSM97251	2580	0	2580	0	89	Male	2	T3	N0	M0
GSM97251	1410	1	1410	0	75	Male	3	T3	N2	M0
GSM97251	1440	1	390	1	85	Male	3	T3	N1	M0
GSM97251	960	1	NA	NA	79	Female	3	T2	N1	M0
GSM97251	3540	0	3540	0	72	Female	2	T3	N0	M0
GSM97251	1980	0	1980	0	82	Male	2	T3	N0	M0
GSM97251	1110	1	960	1	88	Female	2	T3	N0	M0
GSM97252	1860	1	1860	0	76	Male	3	T3	N1	M0

Table S4. A list of cell marker genes

Cells	maker genes
cancer stem cell	CD24
endothelial cell	CDH5, PLVAP, VWF, CLDN5
Fibroblasts	COL1A1, COL1A2, DCN
T cells	CD2, CD3D, CD3E, CD3G
B cells	CD79A, CD79B
Myeloid cells	CD14, LYZ
Mast cells	GATA2, TPSAB1, TPSB2
NK	KLRF1, FGFBP2, KLRC1

Table S5. The DEGs of eight cell types

p_val	avg_logFC	pct.1	pct.2	p_val_adj	cluster	gene	pct.diff
0	2.295997	1	0.863	0	B cells	CD74	0.137
0	2.2336	0.981	0.689	0	B cells	CD79A	0.292
0	2.195389	0.991	0.729	0	B cells	HLA-DRA	0.262
0	2.116231	0.973	0.826	0	B cells	CD83	0.147
0	1.679801	0.653	0.427	0	B cells	IGKV4-1	0.226
0	1.667275	0.984	0.753	0	B cells	HLA-DRB	0.231
0	1.601128	0.951	0.66	0	B cells	HLA-DQA	0.291
0	1.55215	0.987	0.743	0	B cells	HLA-DPA	0.244
0	1.538034	0.952	0.649	0	B cells	HLA-DQB	0.303
0	1.534164	0.974	0.71	0	B cells	HLA-DPB	0.264
0	1.438705	0.821	0.55	0	B cells	IGHM	0.271
0	1.368449	0.921	0.64	0	B cells	IRF8	0.281
0	1.278978	0.979	0.891	0	B cells	KLF2	0.088
0	1.169599	0.911	0.693	0	B cells	LY9	0.218
0	1.166006	0.976	0.889	0	B cells	HERPUD1	0.087
0	1.159173	0.833	0.662	0	B cells	VPREB3	0.171
0	1.107408	0.894	0.682	0	B cells	CD79B	0.212
0	1.07549	0.935	0.651	0	B cells	HLA-DRB	0.284
0	1.044021	0.882	0.671	0	B cells	HLA-DMA	0.211
0	1.010721	0.838	0.684	0	B cells	MEF2C	0.154
0	0.983856	0.9	0.728	0	B cells	NCF1	0.172
0	0.903459	0.839	0.681	0	B cells	LINC0092	0.158
0	0.8961	0.965	0.831	0	B cells	CD55	0.134
0	0.846591	0.802	0.466	0	B cells	POU2AF1	0.336
0	0.840425	0.969	0.919	0	B cells	NR4A1	0.05
0	0.818431	0.762	0.547	0	B cells	SMIM14	0.215
0	0.811881	0.845	0.76	0	B cells	LYN	0.085
0	0.777389	0.905	0.774	0	B cells	SYNGR2	0.131
0	0.76606	0.914	0.819	0	B cells	MARCKSI	0.095
0	0.75379	0.83	0.646	0	B cells	ARHGAP2	0.184
0	0.732164	0.952	0.813	0	B cells	LIMD2	0.139
0	0.731386	0.96	0.886	0	B cells	NR4A2	0.074
0	0.73054	0.741	0.637	0	B cells	ADAM28	0.104
0	0.709496	0.804	0.578	0	B cells	CD40	0.226
0	0.697937	0.767	0.64	0	B cells	HVCN1	0.127
0	0.692632	0.766	0.59	0	B cells	POU2F2	0.176
0	0.687188	0.757	0.704	0	B cells	LY86	0.053
0	0.681705	0.838	0.707	0	B cells	SNX2	0.131
0	0.659091	0.799	0.646	0	B cells	AFF3	0.153
0	0.648468	0.84	0.68	0	B cells	RASGEF1	0.16
0	0.64785	0.794	0.668	0	B cells	HLA-DMB	0.126
0	0.622331	0.761	0.613	0	B cells	SWAP70	0.148
0	0.610756	0.76	0.682	0	B cells	BLNK	0.078
0	0.586323	0.818	0.648	0	B cells	IFT57	0.17
0	0.584539	0.775	0.629	0	B cells	ST6GAL1	0.146
0	0.570446	0.718	0.565	0	B cells	GNG7	0.153
0	0.527792	0.708	0.648	0	B cells	TCF4	0.06
0	0.51997	0.822	0.711	0	B cells	CD24	0.111
0	0.510563	0.786	0.636	0	B cells	ORAI2	0.15
0	0.500815	0.824	0.706	0	B cells	BACH2	0.118
0	0.480611	0.808	0.69	0	B cells	TSPAN3	0.118
0	0.471161	0.806	0.656	0	B cells	TPD52	0.15
0	0.46891	0.926	0.827	0	B cells	RNASET2	0.099
0	0.454638	0.757	0.623	0	B cells	STX7	0.134

0	0.454325	0.87	0.779	0	B cells	ALG13	0.091
0	0.448118	0.703	0.608	0	B cells	BASP1	0.095
0	0.444221	0.726	0.684	0	B cells	RNASE6	0.042
0	0.439545	0.746	0.646	0	B cells	SH3BP5	0.1
0	0.412709	0.734	0.633	0	B cells	TSPAN13	0.101
0	0.360609	0.769	0.647	0	B cells	LHPP	0.122
0	0.331958	0.723	0.575	0	B cells	DAPP1	0.148
0	0.327032	0.768	0.595	0	B cells	BACH1	0.173
0	0.313198	0.76	0.666	0	B cells	KDM4B	0.094
0	0.312391	0.723	0.513	0	B cells	HHEX	0.21
0	0.29702	0.807	0.691	0	B cells	PARVB	0.116
1.76E-303	0.321696	0.627	0.408	3.52E-300	B cells	CIITA	0.219
4.43E-302	0.446323	0.671	0.572	8.87E-299	B cells	BTK	0.099
2.57E-296	0.440567	0.722	0.649	5.14E-293	B cells	SYVN1	0.073
1.25E-287	0.529819	0.711	0.553	2.50E-284	B cells	STAG3	0.158
7.94E-284	0.347114	0.72	0.615	1.59E-280	B cells	SCPEP1	0.105
5.54E-282	0.404615	0.751	0.765	1.11E-278	B cells	CXXC5	-0.014
5.87E-276	0.416981	0.855	0.758	1.17E-272	B cells	TENT5C	0.097
6.19E-274	0.373553	0.756	0.655	1.24E-270	B cells	GGA2	0.101
6.16E-269	0.273642	0.999	0.995	1.23E-265	B cells	EEF1A1	0.004
1.23E-261	0.50093	0.728	0.716	2.47E-258	B cells	LAT2	0.012
2.95E-259	0.365875	0.782	0.67	5.90E-256	B cells	CTSS	0.112
1.51E-258	0.34221	0.745	0.656	3.03E-255	B cells	IFNGR2	0.089
1.92E-258	0.275794	0.668	0.487	3.85E-255	B cells	CD200	0.181
1.03E-253	0.323253	0.861	0.787	2.05E-250	B cells	KDM6B	0.074
3.27E-253	0.336071	0.839	0.789	6.54E-250	B cells	ID3	0.05
4.97E-245	2.23224	0.659	0.539	9.95E-242	B cells	IGKC	0.12
2.38E-236	0.410309	0.966	0.894	4.77E-233	B cells	CD52	0.072
3.59E-235	0.423463	0.781	0.711	7.19E-232	B cells	CTSH	0.07
7.32E-233	0.398414	0.579	0.343	1.46E-229	B cells	IGLC3	0.236
9.59E-231	0.417096	0.852	0.82	1.92E-227	B cells	CHCHD10	0.032
2.87E-230	0.509894	0.659	0.648	5.74E-227	B cells	1-Mar	0.011
3.13E-214	0.283267	0.725	0.595	6.27E-211	B cells	AC007384.	0.13
3.62E-211	0.269254	0.693	0.566	7.24E-208	B cells	PLEKHG1	0.127
6.50E-209	0.353459	0.914	0.878	1.30E-205	B cells	ODC1	0.036
1.30E-202	0.397595	0.739	0.665	2.61E-199	B cells	BCL2A1	0.074
1.99E-200	0.300324	0.639	0.532	3.98E-197	B cells	FCHSD2	0.107
4.68E-197	0.343995	0.87	0.809	9.36E-194	B cells	ANKRD37	0.061
2.49E-192	0.482003	0.878	0.812	4.98E-189	B cells	REL	0.066
8.38E-191	0.453374	0.718	0.719	1.68E-187	B cells	BCAS4	-0.001
1.47E-189	0.320235	0.83	0.75	2.94E-186	B cells	RBKS	0.08
1.62E-187	0.318162	0.762	0.724	3.25E-184	B cells	NFKBID	0.038
1.82E-186	0.318489	0.897	0.852	3.65E-183	B cells	RHOB	0.045
1.49E-173	0.301294	0.73	0.698	2.98E-170	B cells	NCOA3	0.032
2.90E-172	0.361479	0.944	0.916	5.80E-169	B cells	RPL22L1	0.028
1.01E-168	0.389829	0.653	0.648	2.02E-165	B cells	SYK	0.005
4.72E-164	0.467262	0.665	0.725	9.43E-161	B cells	RALGPS2	-0.06
8.66E-160	0.319965	0.686	0.639	1.73E-156	B cells	PRKCB	0.047
1.54E-158	0.293155	0.716	0.672	3.08E-155	B cells	LRMP	0.044
2.35E-157	1.572547	0.577	0.471	4.70E-154	B cells	IGHA1	0.106
3.56E-156	0.379716	0.889	0.806	7.12E-153	B cells	LTB	0.083
5.83E-156	0.270422	0.846	0.704	1.17E-152	B cells	BIRC3	0.142
2.36E-154	0.282873	0.655	0.611	4.71E-151	B cells	LINC0148(	0.044
1.99E-152	0.328268	0.705	0.673	3.97E-149	B cells	KLHL6	0.032
7.09E-152	0.334166	0.996	0.957	1.42E-148	B cells	DUSP1	0.039
2.77E-147	0.3916	0.991	0.94	5.55E-144	B cells	CD69	0.051

4.10E-138	0.320304	0.913	0.785	8.20E-135	B cells	UCP2	0.128
1.07E-136	0.284816	0.571	0.492	2.13E-133	B cells	CPNE5	0.079
4.03E-125	0.267595	0.973	0.95	8.06E-122	B cells	FOSB	0.023
7.72E-125	0.328681	0.853	0.784	1.54E-121	B cells	RGS2	0.069
1.04E-119	0.32242	0.727	0.683	2.08E-116	B cells	FAM3C	0.044
1.70E-118	0.284497	0.934	0.876	3.40E-115	B cells	SLC2A3	0.058
3.97E-118	0.883528	0.672	0.649	7.94E-115	B cells	MZB1	0.023
7.06E-114	0.556708	0.571	0.535	1.41E-110	B cells	DERL3	0.036
2.93E-112	0.273339	0.798	0.754	5.87E-109	B cells	MAP3K8	0.044
2.99E-109	3.574624	0.556	0.482	5.99E-106	B cells	JCHAIN	0.074
1.67E-107	0.328931	0.77	0.765	3.33E-104	B cells	PTPN6	0.005
1.94E-98	0.301992	0.956	0.923	3.88E-95	B cells	ZFP36L1	0.033
4.43E-94	0.460261	0.741	0.724	8.87E-91	B cells	UBE2J1	0.017
3.86E-90	0.265925	0.863	0.813	7.72E-87	B cells	PDE4B	0.05
2.35E-84	0.302173	0.825	0.755	4.70E-81	B cells	CHMP1B	0.07
1.18E-73	0.283752	0.768	0.757	2.35E-70	B cells	SELL	0.011
2.29E-66	0.302438	0.537	0.499	4.59E-63	B cells	FGD2	0.038
1.28E-65	0.407197	0.633	0.585	2.55E-62	B cells	PRPSAP2	0.048
1.04E-46	0.277591	0.631	0.679	2.09E-43	B cells	SEL1L3	-0.048
6.17E-44	0.291797	0.53	0.558	1.23E-40	B cells	STAP1	-0.028
9.13E-22	0.328141	0.823	0.896	1.83E-18	B cells	SSR4	-0.073
0	2.879204	0.982	0.792	0	cancer sten	KRT18	0.19
0	2.634373	0.966	0.777	0	cancer sten	PHGR1	0.189
0	2.372175	0.904	0.751	0	cancer sten	TFF3	0.153
0	1.99778	0.99	0.916	0	cancer sten	S100A6	0.074
0	1.946285	0.898	0.797	0	cancer sten	SOX4	0.101
0	1.592975	0.948	0.832	0	cancer sten	HSPB1	0.116
0	1.521645	0.87	0.777	0	cancer sten	PHLDA2	0.093
0	1.379261	0.831	0.779	0	cancer sten	PERP	0.052
0	1.332617	0.897	0.894	0	cancer sten	NEAT1	0.003
0	1.302628	0.971	0.938	0	cancer sten	MIF	0.033
0	1.260687	0.821	0.673	0	cancer sten	AC103702.	0.148
0	1.242663	0.973	0.999	0	cancer sten	MALAT1	-0.026
0	1.235122	0.878	0.767	0	cancer sten	ANO9	0.111
0	1.156583	0.817	0.769	0	cancer sten	RRBP1	0.048
0	1.143234	0.882	0.778	0	cancer sten	QTRT1	0.104
0	1.138477	0.891	0.829	0	cancer sten	LGALS3	0.062
0	1.051631	0.904	0.851	0	cancer sten	GSTP1	0.053
0	1.044237	0.786	0.821	0	cancer sten	ID1	-0.035
0	1.030806	0.897	0.848	0	cancer sten	TXN	0.049
0	0.966509	0.877	0.822	0	cancer sten	H2AFJ	0.055
0	0.956019	0.795	0.793	0	cancer sten	HOOK2	0.002
0	0.912594	0.841	0.789	0	cancer sten	TNFSF9	0.052
0	0.90943	0.824	0.688	0	cancer sten	EPS8L2	0.136
0	0.896661	0.822	0.738	0	cancer sten	ASL	0.084
0	0.895722	0.927	0.925	0	cancer sten	S100A10	0.002
0	0.893614	0.796	0.708	0	cancer sten	HIST1H2A	0.088
0	0.8845	0.837	0.72	0	cancer sten	CC2D1A	0.117
0	0.847361	0.838	0.739	0	cancer sten	COMTD1	0.099
0	0.841861	0.864	0.742	0	cancer sten	CPNE7	0.122
0	0.841816	0.788	0.755	0	cancer sten	TNFRSF10	0.033
0	0.835825	0.793	0.717	0	cancer sten	CD9	0.076
0	0.826738	0.851	0.726	0	cancer sten	SLC39A4	0.125
0	0.797651	0.995	0.997	0	cancer sten	RPS18	-0.002
0	0.794514	0.732	0.715	0	cancer sten	STARD10	0.017
0	0.78071	0.865	0.78	0	cancer sten	CYC1	0.085

0	0.765345	0.895	0.828	0	cancer sten	CEBPD	0.067
0	0.76424	0.8	0.638	0	cancer sten	TMEM63A	0.162
0	0.735054	0.765	0.724	0	cancer sten	TRIB1	0.041
0	0.728303	0.712	0.674	0	cancer sten	MUC12	0.038
0	0.724465	0.767	0.763	0	cancer sten	TSC22D1	0.004
0	0.650921	0.712	0.697	0	cancer sten	ABHD11	0.015
0	0.647594	0.749	0.743	0	cancer sten	STAP2	0.006
0	0.64199	0.851	0.713	0	cancer sten	CD320	0.138
0	0.641173	0.722	0.713	0	cancer sten	BACE2	0.009
0	0.634065	0.809	0.659	0	cancer sten	SLC22A18	0.15
0	0.628752	0.786	0.708	0	cancer sten	HIST1H2A	0.078
0	0.6197	0.76	0.7	0	cancer sten	AC245014.	0.06
0	0.600208	0.791	0.667	0	cancer sten	PCBD1	0.124
0	0.575193	0.797	0.688	0	cancer sten	AC087239.	0.109
0	0.572397	0.734	0.669	0	cancer sten	DGAT1	0.065
0	0.567344	0.827	0.785	0	cancer sten	NTHL1	0.042
0	0.561393	0.781	0.745	0	cancer sten	HIST1H2A	0.036
0	0.552848	0.737	0.704	0	cancer sten	FTX	0.033
0	0.549912	0.799	0.743	0	cancer sten	CEBPG	0.056
0	0.547787	0.73	0.662	0	cancer sten	AC015912.	0.068
0	0.547177	0.76	0.683	0	cancer sten	MROH1	0.077
0	0.536682	0.847	0.756	0	cancer sten	BOP1	0.091
0	0.511973	0.789	0.714	0	cancer sten	PCK2	0.075
0	0.473709	0.754	0.714	0	cancer sten	MGAT4B	0.04
0	0.460899	0.8	0.749	0	cancer sten	EIF3B	0.051
0	0.43696	0.797	0.761	0	cancer sten	IER5L	0.036
0	0.415516	0.806	0.712	0	cancer sten	F12	0.094
0	0.405058	0.716	0.636	0	cancer sten	HIST1H2B	0.08
0	0.391562	0.816	0.74	0	cancer sten	SLC52A2	0.076
0	0.313769	0.773	0.706	0	cancer sten	EXOSC4	0.067
0	0.307094	0.744	0.625	0	cancer sten	HIST1H2B	0.119
0	0.29996	0.738	0.621	0	cancer sten	NUDT14	0.117
0	0.299264	0.773	0.736	0	cancer sten	COL9A3	0.037
0	0.280242	0.798	0.675	0	cancer sten	SLC38A5	0.123
0	0.273157	0.786	0.758	0	cancer sten	HAGHL	0.028
2.10E-304	0.679751	0.838	0.794	4.19E-301	cancer sten	TIMM13	0.044
5.69E-298	0.60536	0.728	0.682	1.14E-294	cancer sten	ITPR3	0.046
2.20E-290	0.361647	0.743	0.724	4.41E-287	cancer sten	PMEPA1	0.019
7.29E-290	0.307806	0.755	0.68	1.46E-286	cancer sten	AIMP2	0.075
1.00E-286	0.454529	0.743	0.745	2.01E-283	cancer sten	PLEKHA5	-0.002
9.48E-283	0.619802	0.709	0.714	1.90E-279	cancer sten	UGDH	-0.005
1.24E-282	0.568037	0.729	0.718	2.49E-279	cancer sten	LMO7	0.011
5.37E-281	0.391201	0.744	0.736	1.07E-277	cancer sten	DDX11	0.008
7.09E-275	1.013089	0.778	0.799	1.42E-271	cancer sten	IER3	-0.021
5.33E-273	0.786225	0.718	0.761	1.07E-269	cancer sten	MYO7B	-0.043
4.20E-270	0.374224	0.799	0.704	8.39E-267	cancer sten	UQCC2	0.095
1.43E-268	0.652818	0.72	0.727	2.86E-265	cancer sten	CD24	-0.007
4.31E-266	0.424939	0.715	0.723	8.63E-263	cancer sten	HIST1H2A	-0.008
5.35E-264	0.651	0.974	0.978	1.07E-260	cancer sten	GAPDH	-0.004
9.13E-264	0.406839	0.99	0.994	1.83E-260	cancer sten	TMSB10	-0.004
1.61E-263	0.75695	0.896	0.884	3.23E-260	cancer sten	IFITM3	0.012
2.39E-263	0.332236	0.802	0.779	4.79E-260	cancer sten	NOP16	0.023
7.76E-260	0.570445	0.735	0.704	1.55E-256	cancer sten	KCNQ1OT	0.031
1.61E-246	0.315834	0.752	0.726	3.22E-243	cancer sten	STXBP2	0.026
2.90E-245	0.360417	0.838	0.762	5.80E-242	cancer sten	CCND2	0.076
2.59E-240	0.434845	0.71	0.693	5.18E-237	cancer sten	KNOP1	0.017

1.38E-238	0.452207	0.79	0.751	2.75E-235	cancer sten	ZNF593	0.039
5.60E-237	0.787927	0.769	0.718	1.12E-233	cancer sten	PLEC	0.051
1.02E-234	0.343368	0.742	0.661	2.04E-231	cancer sten	POLD2	0.081
1.58E-232	0.305416	0.675	0.705	3.15E-229	cancer sten	NEBL	-0.03
3.09E-231	0.717453	0.919	0.903	6.19E-228	cancer sten	S100A11	0.016
8.70E-220	0.411515	0.805	0.756	1.74E-216	cancer sten	GK	0.049
2.05E-216	0.302603	0.725	0.695	4.11E-213	cancer sten	TARBP1	0.03
8.52E-216	0.66042	0.829	0.751	1.70E-212	cancer sten	SLC7A5	0.078
5.04E-215	0.632163	0.736	0.745	1.01E-211	cancer sten	SPINT2	-0.009
7.48E-214	0.635402	0.732	0.769	1.50E-210	cancer sten	EPS8	-0.037
1.11E-212	0.48643	0.671	0.674	2.21E-209	cancer sten	SPPL2B	-0.003
2.53E-212	0.286342	0.776	0.759	5.06E-209	cancer sten	DNTTIP1	0.017
2.23E-211	0.474841	0.77	0.751	4.46E-208	cancer sten	YWHAG	0.019
6.97E-210	0.429467	0.707	0.662	1.39E-206	cancer sten	MCRIP2	0.045
7.93E-209	0.55215	0.708	0.735	1.59E-205	cancer sten	NUAK2	-0.027
3.88E-207	0.326867	0.675	0.65	7.75E-204	cancer sten	PAXIP1-A!	0.025
4.78E-207	0.345566	0.765	0.723	9.56E-204	cancer sten	USP38	0.042
2.04E-206	0.523259	0.755	0.782	4.07E-203	cancer sten	TRAP1	-0.027
9.89E-206	0.473287	0.717	0.721	1.98E-202	cancer sten	SLC19A2	-0.004
2.09E-201	0.556035	0.717	0.786	4.18E-198	cancer sten	HES1	-0.069
1.31E-196	0.524699	0.759	0.708	2.63E-193	cancer sten	ARL6IP1	0.051
4.90E-195	0.329927	0.77	0.771	9.80E-192	cancer sten	BRI3	-0.001
1.89E-194	0.267418	0.72	0.718	3.77E-191	cancer sten	HIST1H2B	0.002
3.30E-194	0.354889	0.787	0.751	6.61E-191	cancer sten	GGCT	0.036
6.75E-192	0.58663	0.691	0.726	1.35E-188	cancer sten	HIST1H4B	-0.035
2.28E-191	0.483727	0.667	0.669	4.56E-188	cancer sten	CDCA7	-0.002
6.19E-191	0.287706	0.799	0.773	1.24E-187	cancer sten	ZBTB10	0.026
3.52E-187	0.627303	0.831	0.814	7.03E-184	cancer sten	PRDX5	0.017
1.37E-186	0.417538	0.703	0.678	2.75E-183	cancer sten	DIAPH2	0.025
4.14E-184	0.82765	0.653	0.686	8.28E-181	cancer sten	ATP1B1	-0.033
1.65E-183	0.366876	0.731	0.758	3.29E-180	cancer sten	MCM7	-0.027
4.56E-181	0.513006	0.67	0.709	9.12E-178	cancer sten	HIST1H3J	-0.039
5.29E-180	0.327027	0.725	0.695	1.06E-176	cancer sten	CYB5A	0.03
4.08E-179	0.442599	0.64	0.64	8.15E-176	cancer sten	PCCA	0
1.61E-173	0.437646	0.805	0.714	3.21E-170	cancer sten	PHPT1	0.091
1.81E-167	0.418661	0.855	0.844	3.62E-164	cancer sten	CEBPB	0.011
9.57E-166	0.564968	0.728	0.758	1.91E-162	cancer sten	MPST	-0.03
1.18E-164	0.472189	0.72	0.766	2.36E-161	cancer sten	ICA1	-0.046
3.06E-162	0.62474	0.714	0.773	6.12E-159	cancer sten	ETS2	-0.059
7.27E-160	0.347997	0.675	0.663	1.45E-156	cancer sten	SBF2	0.012
1.87E-157	0.449986	0.659	0.689	3.75E-154	cancer sten	HILPDA	-0.03
1.76E-155	0.371047	0.728	0.701	3.52E-152	cancer sten	IARS	0.027
2.20E-153	0.316374	0.716	0.758	4.40E-150	cancer sten	PON2	-0.042
3.06E-150	0.29559	0.651	0.666	6.13E-147	cancer sten	HIST1H2A	-0.015
5.93E-149	0.478219	0.671	0.67	1.19E-145	cancer sten	HIST1H3H	0.001
6.20E-146	0.49152	0.68	0.695	1.24E-142	cancer sten	HIST1H3A	-0.015
1.56E-145	0.679398	0.653	0.701	3.12E-142	cancer sten	TPM1	-0.048
1.97E-142	0.44941	0.838	0.759	3.93E-139	cancer sten	DNPH1	0.079
1.04E-139	0.452946	0.672	0.643	2.08E-136	cancer sten	SQLE	0.029
1.27E-139	0.300235	0.696	0.705	2.53E-136	cancer sten	ILVBL	-0.009
1.11E-137	0.643829	0.815	0.863	2.22E-134	cancer sten	LMNA	-0.048
3.23E-137	0.268408	0.693	0.657	6.45E-134	cancer sten	UCHL3	0.036
8.62E-136	0.502093	0.862	0.862	1.72E-132	cancer sten	PPA1	0
7.09E-135	0.277829	0.667	0.667	1.42E-131	cancer sten	KIF9	0
1.06E-133	0.365159	0.809	0.792	2.12E-130	cancer sten	EIF6	0.017
1.61E-132	0.302882	0.649	0.647	3.22E-129	cancer sten	AC091271.	0.002

6.55E-132	0.499928	0.843	0.784	1.31E-128	cancer sten	AL138963.	0.059
1.88E-128	0.374739	0.706	0.701	3.76E-125	cancer sten	MAP1LC3.	0.005
5.49E-127	0.431065	0.756	0.776	1.10E-123	cancer sten	AHCY	-0.02
2.90E-126	0.503455	0.647	0.69	5.80E-123	cancer sten	ASPH	-0.043
4.97E-125	0.560617	0.663	0.707	9.94E-122	cancer sten	AC023157.	-0.044
8.37E-125	0.338535	0.712	0.709	1.67E-121	cancer sten	HIST1H2B	0.003
2.58E-123	0.711158	0.689	0.728	5.15E-120	cancer sten	HIST1H2A	-0.039
1.15E-122	0.511388	0.68	0.696	2.29E-119	cancer sten	AGPAT2	-0.016
3.05E-121	0.328516	0.701	0.692	6.11E-118	cancer sten	SH3YL1	0.009
3.15E-118	0.738873	0.885	0.878	6.31E-115	cancer sten	ATF3	0.007
3.93E-118	0.619242	0.648	0.688	7.85E-115	cancer sten	GMDS	-0.04
4.30E-115	0.362417	0.719	0.768	8.61E-112	cancer sten	C19orf48	-0.049
7.86E-113	0.470459	0.712	0.756	1.57E-109	cancer sten	BLVRB	-0.044
7.87E-113	0.282896	0.688	0.652	1.57E-109	cancer sten	AP1S1	0.036
1.28E-112	0.495743	0.824	0.824	2.56E-109	cancer sten	CHCHD10	0
8.61E-110	0.790569	0.722	0.781	1.72E-106	cancer sten	PLK2	-0.059
3.37E-109	0.292945	0.772	0.734	6.74E-106	cancer sten	FKBP4	0.038
1.36E-108	0.420633	0.733	0.727	2.73E-105	cancer sten	CISD3	0.006
1.75E-108	0.53705	0.642	0.686	3.50E-105	cancer sten	ANXA4	-0.044
5.73E-106	0.321755	0.629	0.6	1.15E-102	cancer sten	RALGAPA	0.029
1.34E-105	0.274591	0.729	0.747	2.68E-102	cancer sten	DGKD	-0.018
7.00E-104	0.427348	0.685	0.737	1.40E-100	cancer sten	HIP1R	-0.052
1.49E-103	0.306568	0.603	0.549	2.99E-100	cancer sten	MICAL3	0.054
6.33E-102	0.845213	0.735	0.855	1.27E-98	cancer sten	KLF4	-0.12
1.88E-100	0.651006	0.75	0.797	3.76E-97	cancer sten	ACTN4	-0.047
6.96E-98	0.33188	0.702	0.684	1.39E-94	cancer sten	PLCG2	0.018
8.54E-98	0.333356	0.614	0.599	1.71E-94	cancer sten	FASN	0.015
3.83E-96	0.340361	0.686	0.704	7.65E-93	cancer sten	HELZ2	-0.018
1.04E-95	0.458353	0.666	0.709	2.08E-92	cancer sten	TMEM141	-0.043
1.17E-95	0.541384	0.954	0.968	2.34E-92	cancer sten	HMGB1	-0.014
6.78E-95	0.52656	0.726	0.824	1.36E-91	cancer sten	CD151	-0.098
7.86E-91	0.310678	0.908	0.996	1.57E-87	cancer sten	MTRNR2L	-0.088
7.79E-90	0.486106	0.648	0.713	1.56E-86	cancer sten	EPHX2	-0.065
8.24E-84	0.362178	0.609	0.659	1.65E-80	cancer sten	HIST1H3C	-0.05
1.89E-83	0.444643	0.753	0.786	3.79E-80	cancer sten	CHMP4B	-0.033
2.39E-83	0.38309	0.647	0.673	4.78E-80	cancer sten	MKI67	-0.026
6.30E-81	0.271134	0.785	0.763	1.26E-77	cancer sten	EBPL	0.022
3.43E-79	0.52962	0.63	0.714	6.86E-76	cancer sten	PLXNB2	-0.084
7.89E-78	0.328898	0.741	0.736	1.58E-74	cancer sten	TRAF5	0.005
1.33E-77	0.433722	0.684	0.764	2.66E-74	cancer sten	PDLIM1	-0.08
1.31E-76	0.337089	0.665	0.687	2.61E-73	cancer sten	HIST1H2B	-0.022
5.21E-75	0.42933	0.743	0.767	1.04E-71	cancer sten	PTMS	-0.024
2.12E-69	0.614033	0.62	0.69	4.24E-66	cancer sten	MYO1E	-0.07
5.55E-69	0.567201	0.737	0.757	1.11E-65	cancer sten	MXD1	-0.02
5.69E-69	0.358079	0.7	0.707	1.14E-65	cancer sten	NORAD	-0.007
7.81E-69	0.308008	0.683	0.679	1.56E-65	cancer sten	SRSF6	0.004
2.01E-68	0.37605	0.752	0.785	4.01E-65	cancer sten	INTS6	-0.033
4.19E-68	0.544644	0.881	0.942	8.37E-65	cancer sten	SAT1	-0.061
1.26E-66	0.451384	0.688	0.74	2.53E-63	cancer sten	ZKSCAN1	-0.052
1.88E-66	0.572758	0.708	0.714	3.77E-63	cancer sten	HIST1H1E	-0.006
3.98E-65	0.470536	0.662	0.753	7.96E-62	cancer sten	APP	-0.091
1.18E-61	0.498084	0.832	0.844	2.37E-58	cancer sten	CSTB	-0.012
2.45E-60	0.464598	0.852	0.879	4.91E-57	cancer sten	NME2	-0.027
1.64E-59	0.434442	0.781	0.774	3.29E-56	cancer sten	HMGA1	0.007
2.59E-58	0.41198	0.814	0.825	5.18E-55	cancer sten	RPS27L	-0.011
5.72E-58	0.280931	0.695	0.743	1.14E-54	cancer sten	GIPC1	-0.048

1.67E-57	0.266318	0.629	0.605	3.35E-54	cancer sten	ACSL5	0.024
2.64E-56	0.301337	0.568	0.595	5.28E-53	cancer sten	HIST1H4D	-0.027
3.67E-56	0.495268	0.604	0.695	7.34E-53	cancer sten	TNFRSF12	-0.091
1.19E-52	0.346966	0.773	0.767	2.38E-49	cancer sten	PHB	0.006
1.59E-50	0.298503	0.67	0.652	3.18E-47	cancer sten	HIST1H1C	0.018
2.98E-48	0.322549	0.818	0.841	5.96E-45	cancer sten	FABP5	-0.023
1.66E-44	0.406909	0.609	0.682	3.32E-41	cancer sten	HDHD3	-0.073
2.30E-44	0.72698	0.916	0.927	4.60E-41	cancer sten	HSPH1	-0.011
2.39E-44	0.579488	0.919	0.958	4.77E-41	cancer sten	IER2	-0.039
1.36E-42	0.317197	0.584	0.624	2.73E-39	cancer sten	PYGB	-0.04
5.71E-41	0.489914	0.766	0.789	1.14E-37	cancer sten	TPM4	-0.023
1.22E-39	0.386571	0.568	0.589	2.44E-36	cancer sten	PIK3R3	-0.021
4.06E-39	0.324824	0.651	0.752	8.13E-36	cancer sten	LSR	-0.101
5.83E-39	0.310696	0.597	0.579	1.17E-35	cancer sten	RMRP	0.018
9.99E-39	0.356623	0.828	0.878	2.00E-35	cancer sten	RAC1	-0.05
3.36E-37	0.37118	0.686	0.752	6.71E-34	cancer sten	CENPX	-0.066
3.48E-36	0.343657	0.554	0.63	6.97E-33	cancer sten	NQO1	-0.076
1.82E-35	0.273587	0.517	0.703	3.63E-32	cancer sten	NCOA7	-0.186
1.60E-34	0.438854	0.549	0.649	3.20E-31	cancer sten	GALE	-0.1
4.89E-34	0.269425	0.653	0.719	9.78E-31	cancer sten	GPHN	-0.066
1.25E-33	0.468982	0.671	0.745	2.49E-30	cancer sten	HDLBP	-0.074
1.91E-33	0.285981	0.688	0.7	3.81E-30	cancer sten	SLC20A1	-0.012
9.12E-31	0.285156	0.816	0.806	1.82E-27	cancer sten	NUCKS1	0.01
1.74E-29	0.610147	0.891	0.924	3.47E-26	cancer sten	EGR1	-0.033
2.35E-28	0.292308	0.675	0.711	4.70E-25	cancer sten	IRF2BP2	-0.036
1.15E-27	0.332244	0.604	0.669	2.30E-24	cancer sten	HIST1H2B	-0.065
1.36E-26	0.322269	0.867	0.88	2.72E-23	cancer sten	TPI1	-0.013
5.68E-25	0.281796	0.546	0.57	1.14E-21	cancer sten	HIST2H2B	-0.024
3.38E-22	0.334233	0.677	0.709	6.76E-19	cancer sten	FDPS	-0.032
7.53E-22	0.287125	0.593	0.669	1.51E-18	cancer sten	LGALS3BI	-0.076
1.61E-21	0.29056	0.621	0.668	3.22E-18	cancer sten	TRAPPC9	-0.047
4.98E-21	0.356152	0.485	0.658	9.96E-18	cancer sten	ANKRD36	-0.173
3.37E-20	0.283284	0.705	0.736	6.73E-17	cancer sten	SLC25A39	-0.031
2.72E-18	0.293434	0.691	0.697	5.45E-15	cancer sten	AL118516.	-0.006
1.43E-17	0.29508	0.533	0.739	2.87E-14	cancer sten	HMGCS1	-0.206
1.99E-17	0.273396	0.551	0.625	3.97E-14	cancer sten	MYO19	-0.074
2.62E-16	0.482676	0.6	0.674	5.23E-13	cancer sten	LENG8	-0.074
7.31E-16	0.379886	0.79	0.869	1.46E-12	cancer sten	RHOB	-0.079
1.23E-14	0.309503	0.631	0.695	2.46E-11	cancer sten	SUGP2	-0.064
4.04E-12	0.37386	0.598	0.699	8.08E-09	cancer sten	CSRNP2	-0.101
5.10E-12	0.349258	0.698	0.809	1.02E-08	cancer sten	NUDT4	-0.111
1.59E-10	0.288291	0.575	0.673	3.17E-07	cancer sten	SLC9A1	-0.098
2.55E-10	0.30076	0.554	0.638	5.10E-07	cancer sten	CTNNA1	-0.084
3.37E-10	0.448724	0.579	0.701	6.74E-07	cancer sten	NPDC1	-0.122
1.04E-09	0.487401	0.907	0.946	2.08E-06	cancer sten	HSPA1B	-0.039
9.15E-09	0.265495	0.606	0.711	1.83E-05	cancer sten	ISOC2	-0.105
1.54E-08	0.375394	0.537	0.636	3.08E-05	cancer sten	CYSTM1	-0.099
1.58E-08	0.269762	0.582	0.64	3.17E-05	cancer sten	AL135925.	-0.058
1.83E-08	0.329806	0.688	0.773	3.66E-05	cancer sten	GADD45G	-0.085
6.94E-08	0.382251	0.769	0.8	0.000139	cancer sten	GLUD1	-0.031
2.44E-07	0.275269	0.618	0.706	0.000489	cancer sten	CENPF	-0.088
3.92E-06	0.271106	0.513	0.599	0.007832	cancer sten	MYO1C	-0.086
4.84E-06	0.468654	0.611	0.731	0.009676	cancer sten	RAB11FIP	-0.12
9.54E-06	0.268095	0.513	0.583	0.01908	cancer sten	GFPT1	-0.07
4.36E-166	1.881617	0.983	0.576	8.73E-163	endothelial	PECAM1	0.407
7.11E-140	1.159299	0.969	0.679	1.42E-136	endothelial	NPDC1	0.29

1.31E-128	1.426316	0.927	0.656	2.63E-125	endothelial ENG	0.271
2.58E-117	1.193816	1	0.895	5.17E-114	endothelial VIM	0.105
1.11E-105	1.267896	0.976	0.885	2.21E-102	endothelial IFITM3	0.091
1.62E-94	0.969946	0.958	0.778	3.24E-91	endothelial FKBP1A	0.18
3.57E-93	0.835004	0.868	0.523	7.14E-90	endothelial PLXND1	0.345
4.36E-93	0.88513	0.927	0.737	8.72E-90	endothelial APP	0.19
6.12E-90	0.277423	0.962	0.689	1.22E-86	endothelial SFXN3	0.273
6.73E-90	0.958664	0.882	0.653	1.35E-86	endothelial TCF4	0.229
3.46E-86	0.443402	0.948	0.661	6.92E-83	endothelial PLA2G16	0.287
1.34E-84	0.417483	0.944	0.601	2.69E-81	endothelial TPST2	0.343
3.65E-79	0.283713	0.931	0.699	7.29E-76	endothelial PLXNB2	0.232
8.74E-79	0.332124	0.941	0.641	1.75E-75	endothelial RUVBL2	0.3
3.95E-76	0.871676	0.844	0.651	7.91E-73	endothelial MCAM	0.193
7.51E-76	0.759901	0.896	0.701	1.50E-72	endothelial SPTBN1	0.195
2.27E-75	0.908706	0.924	0.794	4.54E-72	endothelial ID3	0.13
7.07E-74	0.565079	0.889	0.75	1.41E-70	endothelial PON2	0.139
3.89E-73	0.368767	0.934	0.657	7.77E-70	endothelial PSMG4	0.277
1.05E-70	0.807047	0.872	0.75	2.10E-67	endothelial PDLIM1	0.122
2.00E-70	0.851362	0.906	0.668	4.00E-67	endothelial ICAM2	0.238
2.99E-69	0.784197	0.948	0.813	5.98E-66	endothelial SELENOW	0.135
1.20E-66	0.562723	0.955	0.684	2.39E-63	endothelial SELENOS	0.271
7.43E-66	0.732337	0.868	0.649	1.49E-62	endothelial STOM	0.219
1.26E-65	0.665811	0.924	0.722	2.53E-62	endothelial CD63	0.202
3.28E-65	0.747815	0.934	0.755	6.56E-62	endothelial TIMP1	0.179
4.06E-65	0.38491	0.924	0.716	8.12E-62	endothelial DDAH2	0.208
1.06E-64	0.776778	0.83	0.583	2.13E-61	endothelial PIK3R3	0.247
4.93E-64	0.824641	0.868	0.731	9.87E-61	endothelial ITGB1	0.137
1.75E-62	0.801751	0.844	0.64	3.51E-59	endothelial PLPP1	0.204
3.00E-61	0.643945	0.882	0.762	6.01E-58	endothelial PTMS	0.12
1.84E-60	0.46034	0.837	0.761	3.67E-57	endothelial KIAA1211	0.076
3.83E-60	0.860914	0.858	0.815	7.67E-57	endothelial ID1	0.043
9.75E-60	0.784791	0.826	0.645	1.95E-56	endothelial SERPINH1	0.181
1.88E-58	0.823944	0.896	0.758	3.77E-55	endothelial ANXA2	0.138
4.47E-58	0.713663	0.906	0.792	8.93E-55	endothelial CD81	0.114
1.12E-56	0.534894	0.878	0.688	2.24E-53	endothelial PHACTR2	0.19
3.68E-56	0.511327	0.799	0.557	7.35E-53	endothelial GIMAP8	0.242
3.76E-56	0.700627	0.844	0.698	7.53E-53	endothelial PTTG1IP	0.146
8.15E-56	1.107466	0.913	0.837	1.63E-52	endothelial FABP5	0.076
1.66E-55	0.486741	0.941	0.689	3.32E-52	endothelial MAGED2	0.252
2.96E-53	0.746265	0.903	0.771	5.92E-50	endothelial DSTN	0.132
2.92E-51	0.300883	0.851	0.611	5.85E-48	endothelial LRRC8C	0.24
1.21E-49	0.485731	0.941	0.87	2.42E-46	endothelial RAC1	0.071
2.41E-49	0.689434	0.927	0.857	4.83E-46	endothelial RHOB	0.07
6.17E-49	0.605225	0.92	0.83	1.23E-45	endothelial MARCKSI	0.09
2.42E-48	0.702153	0.99	0.904	4.83E-45	endothelial IFITM1	0.086
2.67E-48	0.540964	0.861	0.762	5.33E-45	endothelial TSC22D1	0.099
2.58E-47	0.601055	0.83	0.707	5.15E-44	endothelial TNFRSF1A	0.123
3.02E-47	0.754809	0.854	0.809	6.05E-44	endothelial CD151	0.045
6.48E-47	0.311971	0.868	0.674	1.30E-43	endothelial SMC6	0.194
1.05E-46	0.362914	0.962	0.735	2.09E-43	endothelial ATOX1	0.227
1.17E-46	0.273145	0.809	0.501	2.34E-43	endothelial FBLN5	0.308
1.79E-46	0.283295	0.833	0.648	3.59E-43	endothelial DENND3	0.185
3.48E-46	0.355658	0.934	0.737	6.96E-43	endothelial CHST12	0.197
4.03E-46	0.393185	1	0.993	8.07E-43	endothelial TMSB10	0.007
1.21E-45	0.41444	0.854	0.616	2.41E-42	endothelial ELMO1	0.238
5.39E-45	0.442353	0.844	0.757	1.08E-41	endothelial ADGRG1	0.087

1.06E-44	0.27667	0.84	0.609	2.13E-41	endothelial AAMDC	0.231
1.26E-43	0.632058	0.854	0.727	2.52E-40	endothelial LAPTM4A	0.127
1.66E-43	0.42015	0.934	0.772	3.31E-40	endothelial COMT	0.162
1.66E-43	0.706371	0.868	0.685	3.32E-40	endothelial AHNAK	0.183
5.19E-43	0.416538	0.799	0.514	1.04E-39	endothelial GBP4	0.285
3.17E-42	0.487803	0.84	0.692	6.33E-39	endothelial TPM1	0.148
3.63E-42	0.683465	0.764	0.617	7.27E-39	endothelial VAT1	0.147
1.41E-41	0.425393	0.778	0.58	2.81E-38	endothelial PLEKHG1	0.198
4.05E-41	0.404215	0.917	0.784	8.09E-38	endothelial TPM4	0.133
6.53E-41	0.435327	0.868	0.683	1.31E-37	endothelial EI24	0.185
1.33E-40	0.610054	0.778	0.607	2.66E-37	endothelial APLP2	0.171
4.14E-40	0.390757	0.913	0.855	8.28E-37	endothelial LMNA	0.058
5.46E-40	0.342861	0.955	0.766	1.09E-36	endothelial ANXA1	0.189
7.13E-40	0.419098	0.799	0.658	1.43E-36	endothelial SCARB2	0.141
1.24E-39	0.706168	0.75	0.584	2.49E-36	endothelial RDX	0.166
6.27E-39	0.390293	0.76	0.54	1.25E-35	endothelial QKI	0.22
8.47E-39	0.505456	0.753	0.519	1.69E-35	endothelial LMCD1	0.234
1.15E-38	0.350832	0.892	0.691	2.29E-35	endothelial IFNGR1	0.201
1.30E-38	0.500656	0.757	0.607	2.60E-35	endothelial PKP4	0.15
3.07E-38	0.342164	0.854	0.745	6.13E-35	endothelial HEBP1	0.109
4.72E-37	0.390758	0.833	0.694	9.44E-34	endothelial NME4	0.139
4.82E-37	0.48387	0.809	0.752	9.65E-34	endothelial FCGRT	0.057
5.12E-37	0.416413	0.851	0.775	1.02E-33	endothelial HES1	0.076
7.52E-37	0.373377	0.993	0.968	1.50E-33	endothelial ACTG1	0.025
1.27E-35	0.487122	0.84	0.666	2.54E-32	endothelial LGALS1	0.174
1.34E-35	0.39001	0.75	0.519	2.67E-32	endothelial FMNL3	0.231
1.99E-35	0.552342	0.83	0.725	3.97E-32	endothelial PLEC	0.105
3.37E-35	0.47678	0.802	0.638	6.73E-32	endothelial VAMP3	0.164
6.38E-35	0.605406	0.781	0.658	1.28E-31	endothelial SH3BP5	0.123
1.28E-34	0.564929	0.75	0.632	2.56E-31	endothelial BEX3	0.118
1.45E-34	0.623409	0.757	0.655	2.91E-31	endothelial ECE1	0.102
3.96E-34	0.395863	0.823	0.661	7.91E-31	endothelial LUZP1	0.162
4.01E-34	0.303689	0.795	0.635	8.03E-31	endothelial FAM241A	0.16
6.38E-34	0.639334	0.799	0.704	1.28E-30	endothelial ATP1B3	0.095
8.32E-34	0.275041	0.795	0.683	1.66E-30	endothelial GFOD1	0.112
9.79E-34	0.277401	0.812	0.744	1.96E-30	endothelial GRASP	0.068
1.77E-33	0.577059	0.743	0.677	3.53E-30	endothelial COL18A1	0.066
2.55E-33	0.433742	1	0.983	5.09E-30	endothelial MTRNR2L	0.017
6.82E-33	0.36748	0.979	0.759	1.36E-29	endothelial OSTC	0.22
1.14E-32	0.27454	0.84	0.77	2.28E-29	endothelial BRI3	0.07
1.87E-32	0.277509	0.92	0.746	3.74E-29	endothelial NASP	0.174
2.10E-32	0.530532	0.993	0.915	4.21E-29	endothelial DDIT4	0.078
3.35E-32	0.454163	0.812	0.702	6.69E-29	endothelial MLEC	0.11
1.18E-31	0.503174	0.819	0.724	2.36E-28	endothelial FLNA	0.095
2.25E-31	0.325457	0.889	0.685	4.49E-28	endothelial COA3	0.204
2.99E-31	0.367407	0.983	0.952	5.99E-28	endothelial H3F3A	0.031
5.29E-31	0.519203	0.802	0.669	1.06E-27	endothelial VAMP5	0.133
5.44E-31	0.600127	0.753	0.633	1.09E-27	endothelial NFIC	0.12
3.62E-30	0.408669	0.788	0.645	7.24E-27	endothelial IKBIP	0.143
8.53E-30	0.55888	0.944	0.779	1.71E-26	endothelial MT2A	0.165
1.73E-29	0.440084	0.799	0.703	3.45E-26	endothelial GRN	0.096
3.16E-29	0.441632	0.899	0.762	6.32E-26	endothelial SOCS3	0.137
8.19E-29	0.440755	0.844	0.712	1.64E-25	endothelial NPC2	0.132
8.22E-29	0.496678	0.875	0.672	1.64E-25	endothelial BST2	0.203
2.47E-28	0.623365	0.691	0.477	4.94E-25	endothelial NOTCH4	0.214
5.61E-28	0.533344	0.774	0.617	1.12E-24	endothelial TNFSF10	0.157

5.75E-28	0.579282	0.785	0.728	1.15E-24	endothelial CD9	0.057
5.83E-28	0.553941	0.74	0.624	1.17E-24	endothelial CTNNA1	0.116
7.75E-28	0.339057	0.927	0.764	1.55E-24	endothelial TXNDC17	0.163
1.10E-27	0.608834	0.694	0.557	2.20E-24	endothelial HEG1	0.137
3.74E-27	0.395139	0.76	0.615	7.48E-24	endothelial MEF2A	0.145
4.61E-27	0.289063	0.753	0.585	9.22E-24	endothelial CEP170	0.168
6.39E-27	0.420449	0.934	0.815	1.28E-23	endothelial PRDX1	0.119
1.18E-26	0.477371	0.83	0.74	2.37E-23	endothelial GIMAP4	0.09
1.19E-26	0.42009	0.74	0.631	2.39E-23	endothelial SWAP70	0.109
1.55E-25	0.412852	0.823	0.686	3.10E-22	endothelial CSRP1	0.137
2.64E-25	0.433272	0.872	0.732	5.28E-22	endothelial DUT	0.14
3.53E-25	0.33267	0.941	0.925	7.06E-22	endothelial S100A10	0.016
3.57E-25	0.439639	0.764	0.69	7.13E-22	endothelial NEDD9	0.074
3.93E-25	0.325469	0.757	0.646	7.87E-22	endothelial CTSL	0.111
4.96E-25	0.363318	0.712	0.54	9.92E-22	endothelial RTL8A	0.172
8.70E-25	0.352713	0.785	0.65	1.74E-21	endothelial PICALM	0.135
1.08E-22	0.331016	0.826	0.659	2.17E-19	endothelial GIMAP6	0.167
1.32E-22	0.489346	0.74	0.699	2.63E-19	endothelial SPRY1	0.041
2.91E-22	0.421323	0.785	0.698	5.83E-19	endothelial CYB5A	0.087
3.06E-22	0.349922	0.882	0.79	6.12E-19	endothelial HSP90B1	0.092
3.93E-22	0.368755	0.767	0.636	7.85E-19	endothelial NUDT14	0.131
6.02E-22	0.332753	0.934	0.847	1.20E-18	endothelial TUBB	0.087
7.68E-22	0.280451	0.819	0.649	1.54E-18	endothelial PREX1	0.17
1.37E-21	0.284658	0.851	0.837	2.74E-18	endothelial KLF4	0.014
1.94E-21	0.280703	0.753	0.639	3.89E-18	endothelial MAPK3	0.114
1.95E-21	0.38297	0.802	0.709	3.89E-18	endothelial ANXA5	0.093
2.14E-21	0.36689	0.854	0.789	4.28E-18	endothelial ACTN4	0.065
4.67E-21	0.266151	0.861	0.739	9.35E-18	endothelial HSBP1	0.122
1.10E-20	0.399296	0.83	0.683	2.21E-17	endothelial ITM2A	0.147
2.01E-20	0.31607	0.681	0.549	4.02E-17	endothelial RBMS2	0.132
3.88E-20	0.538672	0.698	0.653	7.77E-17	endothelial TMEM204	0.045
4.03E-20	0.306708	0.823	0.674	8.06E-17	endothelial HEXB	0.149
5.14E-20	0.322701	0.951	0.814	1.03E-16	endothelial H2AFY	0.137
5.39E-20	0.594876	0.736	0.669	1.08E-16	endothelial PRCP	0.067
7.33E-20	0.335126	0.91	0.729	1.47E-16	endothelial TALDO1	0.181
2.16E-19	0.46823	0.694	0.559	4.31E-16	endothelial ITGA1	0.135
2.61E-19	0.416442	0.729	0.662	5.23E-16	endothelial SEC14L1	0.067
5.63E-19	0.496105	0.67	0.581	1.13E-15	endothelial LIMS2	0.089
6.25E-19	0.274511	0.701	0.613	1.25E-15	endothelial SGCB	0.088
6.96E-19	0.634846	0.76	0.733	1.39E-15	endothelial CD320	0.027
8.97E-19	0.353944	0.74	0.61	1.79E-15	endothelial TBCD	0.13
9.55E-19	0.296655	0.948	0.874	1.91E-15	endothelial NME2	0.074
9.96E-19	0.341336	0.878	0.756	1.99E-15	endothelial AP2S1	0.122
2.19E-18	0.324695	0.757	0.636	4.39E-15	endothelial GNG10	0.121
2.26E-18	0.278831	0.896	0.717	4.52E-15	endothelial ETHE1	0.179
2.42E-18	0.352396	0.747	0.613	4.85E-15	endothelial MRPL17	0.134
3.04E-18	0.392958	0.875	0.767	6.08E-15	endothelial GIMAP7	0.108
3.89E-18	0.295841	0.799	0.753	7.78E-15	endothelial YWHAH	0.046
9.04E-18	0.371654	0.701	0.596	1.81E-14	endothelial FEZ2	0.105
9.65E-18	0.356931	0.976	0.866	1.93E-14	endothelial LDHA	0.11
5.97E-17	0.372674	0.694	0.587	1.19E-13	endothelial PAM	0.107
1.73E-16	0.356268	0.74	0.651	3.45E-13	endothelial PRDX4	0.089
2.15E-16	0.288422	0.663	0.509	4.29E-13	endothelial IGFBP2	0.154
3.15E-16	0.331194	0.951	0.87	6.30E-13	endothelial TUBA1A	0.081
6.12E-16	0.386315	0.778	0.711	1.22E-12	endothelial ENTPD1	0.067
1.23E-14	0.529302	0.632	0.465	2.46E-11	endothelial BMPR2	0.167

1.24E-14	0.346554	0.764	0.705	2.47E-11	endothelial PARVB	0.059
1.85E-14	0.426819	0.677	0.651	3.69E-11	endothelial APOLD1	0.026
2.43E-14	0.268467	0.792	0.677	4.87E-11	endothelial PDIA4	0.115
6.04E-14	0.343831	0.854	0.765	1.21E-10	endothelial STMN1	0.089
2.65E-13	0.485654	0.691	0.667	5.29E-10	endothelial RIPOR1	0.024
6.43E-13	0.341408	0.767	0.691	1.29E-09	endothelial CRTAP	0.076
7.30E-13	0.276485	0.917	0.843	1.46E-09	endothelial POMP	0.074
1.24E-12	0.455972	0.705	0.705	2.49E-09	endothelial TSPAN3	0
7.37E-12	0.451948	0.622	0.524	1.47E-08	endothelial CRIM1	0.098
9.01E-12	0.522426	0.691	0.733	1.80E-08	endothelial DUSP23	-0.042
1.31E-11	0.317237	0.729	0.656	2.62E-08	endothelial NUCB2	0.073
6.02E-11	0.313114	0.712	0.741	1.20E-07	endothelial SGK1	-0.029
6.41E-11	0.280547	0.694	0.657	1.28E-07	endothelial PDLIM5	0.037
1.47E-10	0.276142	0.733	0.638	2.94E-07	endothelial SPCS3	0.095
2.45E-10	0.372718	0.733	0.771	4.91E-07	endothelial UPP1	-0.038
4.32E-10	0.273257	0.67	0.636	8.63E-07	endothelial CCDC50	0.034
6.66E-10	0.344794	0.712	0.669	1.33E-06	endothelial PLSCR1	0.043
4.29E-09	0.384402	0.688	0.663	8.58E-06	endothelial EIF2AK4	0.025
4.59E-09	0.316197	0.743	0.711	9.18E-06	endothelial YPEL2	0.032
4.87E-09	0.272684	0.701	0.621	9.73E-06	endothelial LPP	0.08
5.89E-07	0.320417	0.649	0.62	0.001178	endothelial RTL8C	0.029
6.33E-07	0.310755	0.691	0.666	0.001266	endothelial KLF9	0.025
6.74E-07	0.328302	0.705	0.675	0.001348	endothelial NCOA7	0.03
9.05E-07	0.267989	0.635	0.637	0.001811	endothelial PTP4A3	-0.002
9.69E-07	0.294944	0.59	0.425	0.001939	endothelial F2R	0.165
3.79E-06	0.330562	0.663	0.644	0.007572	endothelial ZEB1	0.019
4.94E-06	0.3739	0.653	0.713	0.009871	endothelial RGS3	-0.06
7.29E-06	0.295237	0.635	0.598	0.014579	endothelial LEPROT	0.037
7.40E-06	0.383196	0.597	0.552	0.014808	endothelial TRIM47	0.045
1.42E-05	0.313931	0.635	0.609	0.028384	endothelial RHOC	0.026
2.36E-05	0.344997	0.615	0.531	0.047148	endothelial ATL3	0.084
1.76E-299	0.564379	0.975	0.613	3.51E-296	Fibroblasts FXYD1	0.362
1.62E-290	0.439332	0.966	0.565	3.23E-287	Fibroblasts AC245595.	0.401
1.92E-279	1.525789	0.945	0.6	3.83E-276	Fibroblasts MFGE8	0.345
6.25E-274	1.440895	0.951	0.672	1.25E-270	Fibroblasts COL18A1	0.279
4.92E-273	1.164509	0.972	0.753	9.83E-270	Fibroblasts NBL1	0.219
5.96E-271	1.951257	0.981	0.66	1.19E-267	Fibroblasts LGALS1	0.321
1.36E-262	1.358077	0.929	0.681	2.71E-259	Fibroblasts PCOLCE	0.248
6.00E-251	1.710251	0.977	0.752	1.20E-247	Fibroblasts TIMP1	0.225
9.88E-248	1.643583	0.993	0.883	1.98E-244	Fibroblasts IFITM3	0.11
7.08E-241	1.345169	0.996	0.894	1.42E-237	Fibroblasts VIM	0.102
5.41E-240	0.985259	0.937	0.628	1.08E-236	Fibroblasts RAB31	0.309
3.88E-235	1.463685	0.931	0.803	7.77E-232	Fibroblasts COL6A3	0.128
8.28E-228	1.699607	0.965	0.792	1.66E-224	Fibroblasts ID3	0.173
1.81E-225	0.764362	0.942	0.647	3.63E-222	Fibroblasts MCAM	0.295
1.12E-222	1.331255	0.926	0.652	2.24E-219	Fibroblasts LGALS3BI	0.274
1.49E-213	1.21722	0.924	0.688	2.99E-210	Fibroblasts TPM1	0.236
1.76E-207	1.070961	0.887	0.596	3.52E-204	Fibroblasts DKK3	0.291
1.12E-206	0.465531	0.956	0.657	2.24E-203	Fibroblasts GLMP	0.299
1.50E-205	0.603014	0.922	0.625	3.00E-202	Fibroblasts ZMIZ1	0.297
1.25E-204	1.132512	0.949	0.728	2.51E-201	Fibroblasts ITGB1	0.221
6.11E-204	1.299061	0.959	0.719	1.22E-200	Fibroblasts CD63	0.24
4.73E-203	1.210295	0.924	0.651	9.46E-200	Fibroblasts SELENOM	0.273
1.42E-202	0.501175	0.961	0.742	2.84E-199	Fibroblasts HEBP1	0.219
5.78E-196	0.957942	0.907	0.641	1.16E-192	Fibroblasts SERPINH1	0.266
7.56E-193	0.459673	0.956	0.721	1.51E-189	Fibroblasts TMEM9	0.235

3.42E-192	1.235762	0.938	0.724	6.84E-189	Fibroblasts	LAPTM4A	0.214
1.73E-191	0.918595	0.928	0.748	3.45E-188	Fibroblasts	PDLIM1	0.18
3.36E-190	0.991085	0.956	0.759	6.71E-187	Fibroblasts	TSC22D1	0.197
4.69E-189	0.730343	0.958	0.69	9.38E-186	Fibroblasts	MT1E	0.268
1.26E-188	0.490163	0.922	0.676	2.52E-185	Fibroblasts	LGMN	0.246
1.99E-187	0.597545	0.954	0.722	3.98E-184	Fibroblasts	KLF10	0.232
9.49E-183	0.39114	0.958	0.586	1.90E-179	Fibroblasts	PGM1	0.372
2.60E-177	1.224479	0.894	0.695	5.21E-174	Fibroblasts	SPON2	0.199
7.94E-176	0.387636	0.882	0.613	1.59E-172	Fibroblasts	ST3GAL4	0.269
3.60E-175	0.87793	0.959	0.731	7.20E-172	Fibroblasts	GLUL	0.228
1.04E-173	0.484685	0.919	0.622	2.08E-170	Fibroblasts	SCPEP1	0.297
1.03E-172	0.922351	0.919	0.76	2.05E-169	Fibroblasts	PTMS	0.159
2.38E-172	0.604998	0.907	0.591	4.75E-169	Fibroblasts	LEPROT	0.316
8.77E-172	0.288615	0.901	0.55	1.75E-168	Fibroblasts	STAT2	0.351
2.55E-170	0.28164	0.931	0.682	5.10E-167	Fibroblasts	RASD1	0.249
1.84E-168	0.929001	0.845	0.568	3.69E-165	Fibroblasts	COL5A1	0.277
7.70E-168	0.35885	0.949	0.687	1.54E-164	Fibroblasts	ZBTB16	0.262
2.80E-166	0.673147	0.933	0.735	5.59E-163	Fibroblasts	SMIM3	0.198
1.22E-165	0.610871	0.891	0.669	2.44E-162	Fibroblasts	TNS3	0.222
8.41E-165	1.068246	0.933	0.769	1.68E-161	Fibroblasts	DSTN	0.164
1.56E-164	0.304167	0.892	0.58	3.12E-161	Fibroblasts	CYTH3	0.312
2.64E-164	1.585245	0.864	0.544	5.27E-161	Fibroblasts	ACTA2	0.32
1.33E-163	0.835035	0.905	0.736	2.67E-160	Fibroblasts	APP	0.169
1.36E-163	1.001908	0.952	0.705	2.72E-160	Fibroblasts	ANXA5	0.247
1.41E-159	0.868896	0.889	0.636	2.81E-156	Fibroblasts	PLPP1	0.253
7.72E-159	0.772464	0.97	0.853	1.54E-155	Fibroblasts	LMNA	0.117
8.42E-159	0.969008	0.887	0.725	1.68E-155	Fibroblasts	LTBP4	0.162
7.72E-158	0.582796	0.919	0.703	1.54E-154	Fibroblasts	SPATS2L	0.216
1.92E-156	0.917677	0.905	0.687	3.85E-153	Fibroblasts	CRTAP	0.218
2.20E-156	0.55073	0.951	0.723	4.40E-153	Fibroblasts	CD9	0.228
2.34E-153	0.506894	0.896	0.642	4.68E-150	Fibroblasts	CTSL	0.254
2.09E-152	0.812048	0.864	0.628	4.19E-149	Fibroblasts	BEX3	0.236
2.87E-152	0.432528	0.951	0.681	5.74E-149	Fibroblasts	COA3	0.27
3.80E-152	0.527292	0.898	0.613	7.60E-149	Fibroblasts	VAT1	0.285
7.08E-152	0.769093	0.885	0.628	1.42E-148	Fibroblasts	NFIC	0.257
4.98E-150	0.514094	0.924	0.619	9.96E-147	Fibroblasts	CTNNA1	0.305
6.83E-150	0.784965	0.924	0.699	1.37E-146	Fibroblasts	GRN	0.225
3.55E-149	0.722511	0.922	0.645	7.10E-146	Fibroblasts	STOM	0.277
7.13E-147	0.300693	0.917	0.657	1.43E-143	Fibroblasts	EIF2AK4	0.26
9.16E-147	0.511216	0.869	0.534	1.83E-143	Fibroblasts	RTL8A	0.335
1.81E-145	0.272355	0.947	0.639	3.62E-142	Fibroblasts	BCL7C	0.308
1.60E-142	0.781504	0.97	0.854	3.20E-139	Fibroblasts	PPIB	0.116
2.63E-141	0.534826	0.961	0.789	5.26E-138	Fibroblasts	CD81	0.172
4.34E-141	0.424472	0.956	0.756	8.68E-138	Fibroblasts	GADD45G	0.2
7.24E-141	0.518648	0.841	0.497	1.45E-137	Fibroblasts	FBLN5	0.344
4.54E-135	0.512915	0.956	0.835	9.07E-132	Fibroblasts	KLF4	0.121
7.23E-135	0.267735	0.903	0.633	1.45E-131	Fibroblasts	GSTM2	0.27
1.18E-132	0.647078	0.951	0.708	2.36E-129	Fibroblasts	NPC2	0.243
1.85E-132	0.405424	0.908	0.718	3.70E-129	Fibroblasts	SPTSSA	0.19
3.32E-132	0.743937	0.947	0.782	6.65E-129	Fibroblasts	TPM4	0.165
1.05E-131	0.309523	0.852	0.575	2.10E-128	Fibroblasts	GPX7	0.277
3.10E-126	0.421675	0.896	0.681	6.21E-123	Fibroblasts	TCEAL8	0.215
1.52E-125	1.354327	0.965	0.776	3.04E-122	Fibroblasts	MT2A	0.189
3.70E-124	0.274179	0.944	0.67	7.39E-121	Fibroblasts	ALG5	0.274
6.42E-124	0.362821	0.912	0.614	1.28E-120	Fibroblasts	SIGMAR1	0.298
1.37E-123	0.456661	0.869	0.661	2.73E-120	Fibroblasts	KLF9	0.208

1.84E-123	0.713311	0.954	0.799	3.68E-120	Fibroblasts	PSAP	0.155
6.98E-123	0.848716	0.908	0.72	1.40E-119	Fibroblasts	FLNA	0.188
2.58E-122	0.313159	0.912	0.722	5.16E-119	Fibroblasts	PMEPA1	0.19
2.94E-122	0.834413	0.938	0.756	5.89E-119	Fibroblasts	ANXA2	0.182
2.12E-120	0.610363	0.82	0.583	4.25E-117	Fibroblasts	PAM	0.237
4.11E-118	0.551937	0.831	0.634	8.22E-115	Fibroblasts	VCL	0.197
6.75E-118	0.561527	0.966	0.769	1.35E-114	Fibroblasts	MT1X	0.197
7.88E-118	0.322526	0.959	0.711	1.58E-114	Fibroblasts	MRPL36	0.248
9.40E-118	0.549676	0.848	0.649	1.88E-114	Fibroblasts	TMEM204	0.199
1.62E-117	0.356656	0.921	0.664	3.24E-114	Fibroblasts	PRCP	0.257
2.59E-116	0.72794	0.958	0.835	5.18E-113	Fibroblasts	CEBPD	0.123
7.43E-116	0.721336	0.843	0.711	1.49E-112	Fibroblasts	ACTN1	0.132
7.93E-116	0.632529	0.963	0.868	1.59E-112	Fibroblasts	RAC1	0.095
7.37E-114	0.576671	0.834	0.681	1.47E-110	Fibroblasts	PKIG	0.153
8.29E-114	0.656745	0.831	0.616	1.66E-110	Fibroblasts	RTL8C	0.215
1.37E-113	0.314758	0.887	0.661	2.74E-110	Fibroblasts	HYI	0.226
2.09E-112	0.381454	0.942	0.647	4.19E-109	Fibroblasts	AKR1B1	0.295
4.01E-112	0.50712	0.998	0.991	8.01E-109	Fibroblasts	FTH1	0.007
2.18E-111	0.617542	0.917	0.73	4.36E-108	Fibroblasts	HDLBP	0.187
2.12E-109	0.54436	0.834	0.652	4.25E-106	Fibroblasts	TCF4	0.182
2.42E-109	0.462711	0.869	0.627	4.83E-106	Fibroblasts	OTULINL	0.242
6.99E-109	0.629839	0.892	0.808	1.40E-105	Fibroblasts	CD151	0.084
9.65E-108	0.363731	0.91	0.666	1.93E-104	Fibroblasts	LMO4	0.244
7.90E-107	0.504817	0.91	0.687	1.58E-103	Fibroblasts	MAGED2	0.223
1.15E-106	0.49261	0.84	0.707	2.29E-103	Fibroblasts	CHPF	0.133
4.15E-106	0.464897	0.949	0.716	8.30E-103	Fibroblasts	TM9SF2	0.233
1.38E-105	0.468382	0.857	0.642	2.76E-102	Fibroblasts	IKBIP	0.215
3.39E-105	0.711587	0.915	0.797	6.78E-102	Fibroblasts	P4HB	0.118
8.86E-103	0.720746	0.966	0.811	1.77E-99	Fibroblasts	SELENOW	0.155
3.16E-101	0.733641	0.857	0.684	6.32E-98	Fibroblasts	CSRP1	0.173
2.00E-99	0.73382	0.824	0.718	3.99E-96	Fibroblasts	GEM	0.106
6.64E-99	0.337623	0.924	0.675	1.33E-95	Fibroblasts	C20orf27	0.249
9.51E-97	0.422237	0.882	0.774	1.90E-93	Fibroblasts	RRBP1	0.108
5.69E-95	0.476182	0.794	0.54	1.14E-91	Fibroblasts	ISYNA1	0.254
2.24E-94	0.576447	0.806	0.666	4.48E-91	Fibroblasts	PDLIM7	0.14
2.51E-94	0.294794	0.974	0.757	5.01E-91	Fibroblasts	MRPL51	0.217
8.49E-93	0.36563	0.882	0.677	1.70E-89	Fibroblasts	FOXO3	0.205
2.99E-92	0.689755	0.792	0.622	5.98E-89	Fibroblasts	CALU	0.17
2.27E-91	0.507583	0.979	0.807	4.53E-88	Fibroblasts	EIF5A	0.172
1.46E-89	0.592974	0.952	0.759	2.93E-86	Fibroblasts	SOCS3	0.193
4.71E-89	0.34696	0.855	0.627	9.42E-86	Fibroblasts	SLC39A7	0.228
1.60E-88	0.394183	0.961	0.835	3.20E-85	Fibroblasts	FABP5	0.126
3.04E-86	0.421567	0.938	0.771	6.09E-83	Fibroblasts	MIR4435-2	0.167
3.78E-86	0.437432	0.864	0.75	7.56E-83	Fibroblasts	FCGRT	0.114
3.95E-86	0.593913	0.827	0.702	7.91E-83	Fibroblasts	TSPAN3	0.125
9.20E-86	0.680537	0.954	0.846	1.84E-82	Fibroblasts	TUBB	0.108
9.48E-86	0.411552	0.877	0.615	1.90E-82	Fibroblasts	CRELD2	0.262
1.96E-83	0.534109	0.875	0.658	3.91E-80	Fibroblasts	ITM2C	0.217
1.64E-81	0.464986	0.857	0.678	3.28E-78	Fibroblasts	PLD3	0.179
2.81E-81	0.493247	0.79	0.657	5.62E-78	Fibroblasts	SCARB2	0.133
2.57E-80	0.286648	0.956	0.847	5.13E-77	Fibroblasts	HSPB1	0.109
4.39E-80	0.490887	0.929	0.788	8.77E-77	Fibroblasts	HSP90B1	0.141
9.44E-80	0.478987	0.762	0.611	1.89E-76	Fibroblasts	SGCB	0.151
1.55E-79	0.538205	0.841	0.699	3.11E-76	Fibroblasts	MAP1LC3.	0.142
1.62E-79	0.439591	0.84	0.706	3.24E-76	Fibroblasts	TNFRSF1A	0.134
3.54E-79	0.471769	0.854	0.632	7.09E-76	Fibroblasts	PTP4A3	0.222

8.87E-79	0.427023	0.94	0.716	1.77E-75	Fibroblasts PRNP	0.224
2.07E-75	0.414118	0.898	0.737	4.15E-72	Fibroblasts HSBP1	0.161
2.32E-75	0.450721	0.758	0.578	4.65E-72	Fibroblasts P3H1	0.18
5.95E-75	0.412765	0.824	0.654	1.19E-71	Fibroblasts PDLIM5	0.17
7.42E-75	0.393256	0.998	0.974	1.48E-71	Fibroblasts FTL	0.024
2.52E-74	0.536545	0.959	0.802	5.04E-71	Fibroblasts PKM	0.157
3.05E-74	0.520393	0.891	0.752	6.11E-71	Fibroblasts KDELR2	0.139
3.95E-74	0.441474	0.878	0.733	7.91E-71	Fibroblasts IL6ST	0.145
8.22E-74	0.437621	0.926	0.771	1.64E-70	Fibroblasts COMT	0.155
3.57E-73	0.322844	0.808	0.632	7.14E-70	Fibroblasts CSF1	0.176
1.15E-71	0.366444	0.88	0.814	2.31E-68	Fibroblasts ID1	0.066
1.96E-71	0.329619	0.817	0.671	3.91E-68	Fibroblasts FNDC3B	0.146
3.86E-70	0.493813	0.905	0.692	7.72E-67	Fibroblasts RPN2	0.213
2.88E-69	0.58135	0.854	0.683	5.76E-66	Fibroblasts AHNAK	0.171
7.74E-68	0.447199	0.91	0.769	1.55E-64	Fibroblasts MYDGF	0.141
2.69E-66	0.435655	0.788	0.688	5.37E-63	Fibroblasts PHACTR2	0.1
3.50E-66	0.349629	0.905	0.724	7.01E-63	Fibroblasts ADI1	0.181
3.58E-66	0.301501	0.85	0.582	7.16E-63	Fibroblasts ZEB2	0.268
2.28E-65	0.380462	0.908	0.689	4.57E-62	Fibroblasts CNIH1	0.219
6.26E-65	0.48684	0.869	0.672	1.25E-61	Fibroblasts HEXB	0.197
1.24E-64	0.436388	0.818	0.7	2.49E-61	Fibroblasts SPTBN1	0.118
2.75E-64	0.619679	0.989	0.926	5.51E-61	Fibroblasts ZFP36L1	0.063
1.12E-63	0.355979	0.797	0.661	2.24E-60	Fibroblasts FUCA2	0.136
1.17E-63	0.319095	0.917	0.705	2.34E-60	Fibroblasts FKBP2	0.212
3.09E-62	0.602118	0.972	0.918	6.19E-59	Fibroblasts EGR1	0.054
5.44E-62	0.442706	0.908	0.705	1.09E-58	Fibroblasts SSR3	0.203
6.88E-62	0.494197	0.859	0.736	1.38E-58	Fibroblasts CANX	0.123
7.29E-62	0.437032	0.792	0.731	1.46E-58	Fibroblasts TCEAL4	0.061
1.31E-61	0.361871	0.764	0.612	2.62E-58	Fibroblasts MAGED1	0.152
1.17E-60	0.538298	0.97	0.869	2.34E-57	Fibroblasts TUBA1A	0.101
3.47E-59	0.326436	0.977	0.904	6.95E-56	Fibroblasts S100A11	0.073
4.58E-59	0.575897	0.984	0.901	9.17E-56	Fibroblasts GADD45B	0.083
1.72E-58	0.386025	0.832	0.61	3.44E-55	Fibroblasts TYMP	0.222
4.27E-57	0.416639	0.875	0.751	8.54E-54	Fibroblasts YWHAH	0.124
5.87E-57	0.277868	0.979	0.804	1.17E-53	Fibroblasts NUCKS1	0.175
1.01E-56	0.284578	0.877	0.666	2.03E-53	Fibroblasts VAMP5	0.211
1.09E-55	0.408436	0.982	0.841	2.18E-52	Fibroblasts SEC61B	0.141
5.32E-55	0.523308	0.785	0.698	1.06E-51	Fibroblasts PTTG1IP	0.087
7.36E-55	0.302448	0.855	0.694	1.47E-51	Fibroblasts UGCG	0.161
1.91E-54	0.42381	0.841	0.725	3.82E-51	Fibroblasts CTSB	0.116
7.39E-54	0.263169	0.764	0.591	1.48E-50	Fibroblasts MAGEF1	0.173
1.10E-53	0.512858	0.743	0.604	2.20E-50	Fibroblasts WARS	0.139
1.50E-53	0.488113	0.794	0.659	3.01E-50	Fibroblasts ERLEC1	0.135
3.97E-53	0.455359	0.937	0.813	7.94E-50	Fibroblasts PRDX1	0.124
1.88E-52	0.424582	0.774	0.65	3.77E-49	Fibroblasts PRDX4	0.124
2.65E-52	0.369287	0.914	0.755	5.29E-49	Fibroblasts EMP3	0.159
9.50E-52	0.40635	0.901	0.73	1.90E-48	Fibroblasts DUT	0.171
1.14E-51	0.424675	0.951	0.855	2.28E-48	Fibroblasts RHOB	0.096
1.31E-51	0.307558	0.753	0.657	2.62E-48	Fibroblasts ENG	0.096
1.88E-50	0.277286	0.78	0.593	3.77E-47	Fibroblasts FEZ2	0.187
2.03E-50	0.359303	0.799	0.61	4.06E-47	Fibroblasts CCPG1	0.189
3.96E-50	0.458552	0.854	0.772	7.91E-47	Fibroblasts PHLDA1	0.082
4.72E-50	0.405809	0.792	0.704	9.44E-47	Fibroblasts NORAD	0.088
8.27E-50	0.3491	0.732	0.563	1.65E-46	Fibroblasts CPQ	0.169
1.98E-49	0.367732	0.982	0.838	3.96E-46	Fibroblasts CSTB	0.144
5.04E-49	0.50205	0.85	0.735	1.01E-45	Fibroblasts CTSC	0.115

2.21E-48	0.355178	0.781	0.668	4.42E-45	Fibroblasts	QSOX1	0.113
6.10E-48	0.409563	0.995	0.903	1.22E-44	Fibroblasts	IFITM1	0.092
1.03E-47	0.404549	0.887	0.806	2.06E-44	Fibroblasts	CLTA	0.081
1.91E-47	0.409607	0.78	0.694	3.82E-44	Fibroblasts	NME4	0.086
1.34E-46	0.284141	0.831	0.687	2.67E-43	Fibroblasts	AGTRAP	0.144
2.29E-46	0.296885	0.772	0.634	4.58E-43	Fibroblasts	GNG10	0.138
4.21E-46	0.308344	0.744	0.644	8.42E-43	Fibroblasts	FHL3	0.1
5.77E-45	0.402179	0.908	0.76	1.15E-41	Fibroblasts	PGAM1	0.148
9.92E-44	0.322049	0.942	0.843	1.98E-40	Fibroblasts	MYADM	0.099
1.82E-43	0.345328	0.815	0.658	3.63E-40	Fibroblasts	CTNNB1	0.157
1.26E-42	0.366907	0.802	0.676	2.52E-39	Fibroblasts	CTSA	0.126
1.52E-41	0.344388	0.877	0.794	3.05E-38	Fibroblasts	IER3	0.083
2.91E-41	0.358938	0.836	0.697	5.82E-38	Fibroblasts	IMPDH2	0.139
1.17E-40	0.392452	0.771	0.674	2.33E-37	Fibroblasts	REXO2	0.097
3.88E-40	0.367394	0.827	0.719	7.75E-37	Fibroblasts	PDIA6	0.108
9.82E-40	0.264474	0.924	0.836	1.96E-36	Fibroblasts	LGALS3	0.088
1.91E-39	0.350285	0.924	0.776	3.82E-36	Fibroblasts	FKBP1A	0.148
2.26E-39	0.362215	0.753	0.554	4.52E-36	Fibroblasts	IFI6	0.199
4.58E-39	0.389746	0.908	0.795	9.15E-36	Fibroblasts	SOD2	0.113
1.39E-38	0.273448	0.965	0.784	2.79E-35	Fibroblasts	ARPC5	0.181
2.35E-38	0.399971	0.947	0.904	4.69E-35	Fibroblasts	TUBA1B	0.043
5.09E-37	0.39123	0.698	0.557	1.02E-33	Fibroblasts	ITGA1	0.141
9.94E-37	0.284202	0.817	0.763	1.99E-33	Fibroblasts	ETS2	0.054
2.47E-36	0.336278	0.958	0.873	4.94E-33	Fibroblasts	NME2	0.085
1.33E-35	0.374915	0.728	0.671	2.65E-32	Fibroblasts	WASL	0.057
6.91E-34	0.575209	0.991	0.978	1.38E-30	Fibroblasts	FOS	0.013
2.06E-33	0.267812	0.82	0.684	4.12E-30	Fibroblasts	SELENOS	0.136
2.58E-33	0.323306	0.843	0.707	5.16E-30	Fibroblasts	UQCRC1	0.136
6.68E-33	0.270568	0.97	0.856	1.34E-29	Fibroblasts	HMGN2	0.114
1.13E-32	0.296103	0.861	0.724	2.25E-29	Fibroblasts	PHPT1	0.137
1.98E-32	0.365341	0.728	0.62	3.95E-29	Fibroblasts	LPP	0.108
1.24E-31	0.293339	0.975	0.873	2.47E-28	Fibroblasts	HSPA1A	0.102
1.40E-30	0.339452	0.776	0.764	2.81E-27	Fibroblasts	EPS8	0.012
2.89E-30	0.273084	0.753	0.591	5.78E-27	Fibroblasts	GSTO1	0.162
8.12E-30	0.305007	0.67	0.518	1.62E-26	Fibroblasts	LMCD1	0.152
8.34E-30	0.354098	0.949	0.866	1.67E-26	Fibroblasts	ZFAND5	0.083
1.60E-29	0.354799	0.774	0.673	3.19E-26	Fibroblasts	SSRP1	0.101
4.98E-29	0.370344	0.795	0.747	9.97E-26	Fibroblasts	ICAM1	0.048
5.02E-28	0.279371	0.698	0.624	1.00E-24	Fibroblasts	TCEAL3	0.074
6.47E-28	0.265673	0.848	0.759	1.29E-24	Fibroblasts	OSTC	0.089
2.26E-27	0.341357	0.78	0.686	4.52E-24	Fibroblasts	TMED3	0.094
3.59E-26	0.294465	0.746	0.62	7.18E-23	Fibroblasts	LMAN1	0.126
2.82E-25	0.281514	0.928	0.842	5.64E-22	Fibroblasts	POMP	0.086
8.20E-24	0.327392	0.637	0.422	1.64E-20	Fibroblasts	F2R	0.215
7.44E-23	0.320234	0.709	0.642	1.49E-19	Fibroblasts	ZEB1	0.067
3.86E-22	0.376493	0.693	0.607	7.72E-19	Fibroblasts	APLP2	0.086
1.71E-21	0.276205	0.698	0.681	3.41E-18	Fibroblasts	TNFRSF12	0.017
1.17E-20	0.278563	0.704	0.655	2.34E-17	Fibroblasts	ECE1	0.049
4.00E-10	0.263371	0.905	0.858	7.99E-07	Fibroblasts	CDKN1A	0.047
1.64E-70	0.824398	0.975	0.759	3.29E-67	Mast cells	AF213884.	0.216
7.60E-63	0.681469	0.975	0.612	1.52E-59	Mast cells	SLC44A1	0.363
1.88E-62	0.630018	0.967	0.638	3.77E-59	Mast cells	GSTM2	0.329
3.71E-61	0.267094	0.984	0.643	7.42E-58	Mast cells	UBAC1	0.341
3.09E-60	1.358892	0.926	0.5	6.19E-57	Mast cells	LTC4S	0.426
4.38E-59	1.447693	0.967	0.735	8.76E-56	Mast cells	GLUL	0.232
1.74E-58	0.426635	0.959	0.598	3.47E-55	Mast cells	WDFY1	0.361

3.55E-58	1.820988	0.943	0.715	7.10E-55	Mast cells	HPGD	0.228
9.36E-58	0.941807	0.992	0.837	1.87E-54	Mast cells	NFKB1	0.155
1.13E-57	0.71666	0.975	0.691	2.26E-54	Mast cells	RIN3	0.284
2.52E-57	0.319924	0.984	0.658	5.05E-54	Mast cells	MTHFD1	0.326
4.38E-57	0.420956	0.967	0.653	8.76E-54	Mast cells	SIAH1	0.314
1.42E-56	1.260488	1	0.842	2.83E-53	Mast cells	NFKBIZ	0.158
1.10E-55	0.328031	0.984	0.684	2.19E-52	Mast cells	TCEAL8	0.3
3.16E-55	0.314347	0.967	0.737	6.32E-52	Mast cells	RRP12	0.23
7.11E-54	0.538335	0.992	0.705	1.42E-50	Mast cells	DUSP10	0.287
7.89E-54	0.316865	0.959	0.63	1.58E-50	Mast cells	WDR77	0.329
2.82E-53	0.297682	0.951	0.58	5.64E-50	Mast cells	DSE	0.371
3.94E-53	1.13423	1	0.756	7.88E-50	Mast cells	TIMP1	0.244
7.81E-53	0.859956	0.992	0.74	1.56E-49	Mast cells	NR4A3	0.252
1.18E-52	1.681854	0.975	0.871	2.35E-49	Mast cells	AREG	0.104
1.49E-52	0.277481	0.943	0.634	2.99E-49	Mast cells	TRERF1	0.309
3.98E-52	0.664059	0.934	0.665	7.97E-49	Mast cells	ADRB2	0.269
8.21E-52	0.966674	0.951	0.678	1.64E-48	Mast cells	TYROBP	0.273
1.30E-51	0.832992	1	0.991	2.61E-48	Mast cells	FTH1	0.009
2.85E-50	0.566781	0.959	0.626	5.71E-47	Mast cells	PCNX4	0.333
3.85E-50	1.169009	0.885	0.504	7.70E-47	Mast cells	VWA5A	0.381
5.30E-50	0.414443	0.975	0.67	1.06E-46	Mast cells	ZDHHC12	0.305
7.52E-50	0.584265	0.943	0.638	1.50E-46	Mast cells	GNA15	0.305
3.07E-49	0.623543	0.984	0.739	6.14E-46	Mast cells	IL4R	0.245
3.66E-49	0.410911	0.975	0.63	7.31E-46	Mast cells	STX11	0.345
4.57E-48	0.957971	0.926	0.64	9.14E-45	Mast cells	RNF130	0.286
1.17E-47	1.132261	0.91	0.667	2.34E-44	Mast cells	FCER1G	0.243
1.41E-47	0.380656	0.943	0.581	2.82E-44	Mast cells	RASSF2	0.362
1.77E-47	0.695155	0.959	0.747	3.55E-44	Mast cells	ICAM1	0.212
2.51E-46	0.35595	0.951	0.699	5.03E-43	Mast cells	3-Mar	0.252
2.77E-46	0.793614	0.951	0.671	5.54E-43	Mast cells	LMO4	0.28
1.13E-45	0.515267	0.984	0.763	2.26E-42	Mast cells	BCL3	0.221
1.45E-45	1.032047	0.992	0.896	2.90E-42	Mast cells	VIM	0.096
1.64E-45	0.85342	0.951	0.738	3.28E-42	Mast cells	SMIM3	0.213
1.94E-45	0.971102	0.992	0.855	3.88E-42	Mast cells	LMNA	0.137
3.88E-45	0.266566	0.959	0.707	7.76E-42	Mast cells	SPATS2L	0.252
7.60E-45	0.590154	0.975	0.662	1.52E-41	Mast cells	ANKRD28	0.313
1.49E-44	0.902681	0.926	0.694	2.98E-41	Mast cells	TENT5A	0.232
5.75E-44	0.593495	0.934	0.654	1.15E-40	Mast cells	IL18R1	0.28
1.15E-43	0.851963	0.984	0.818	2.31E-40	Mast cells	AC020916.	0.166
1.31E-43	0.482096	0.926	0.683	2.62E-40	Mast cells	TSC22D2	0.243
7.65E-43	0.437681	0.918	0.643	1.53E-39	Mast cells	SPTLC2	0.275
9.01E-43	0.513425	0.893	0.535	1.80E-39	Mast cells	AC084033.	0.358
5.99E-42	1.008222	0.992	0.844	1.20E-38	Mast cells	MYADM	0.148
2.28E-41	1.01921	0.943	0.724	4.55E-38	Mast cells	CD63	0.219
3.85E-41	0.451785	0.975	0.703	7.71E-38	Mast cells	TIPARP	0.272
9.00E-41	0.313577	0.984	0.738	1.80E-37	Mast cells	SHMT2	0.246
9.55E-41	0.862032	0.918	0.657	1.91E-37	Mast cells	ARHGAP1	0.261
1.46E-40	0.757069	0.902	0.618	2.92E-37	Mast cells	VAT1	0.284
1.83E-40	0.519818	0.885	0.631	3.66E-37	Mast cells	GNS	0.254
2.84E-40	0.767768	0.959	0.737	5.69E-37	Mast cells	GPR65	0.222
3.90E-40	0.661498	0.902	0.63	7.81E-37	Mast cells	BEX4	0.272
2.37E-39	0.829619	0.934	0.728	4.75E-36	Mast cells	CAPG	0.206
3.18E-39	0.929666	0.885	0.635	6.37E-36	Mast cells	CSF1	0.25
3.38E-39	0.789462	0.902	0.728	6.76E-36	Mast cells	CD9	0.174
6.11E-39	1.095563	0.984	0.854	1.22E-35	Mast cells	SOCS1	0.13
6.42E-39	0.645459	0.984	0.757	1.28E-35	Mast cells	MRPS6	0.227

1.80E-38	0.49262	0.943	0.629	3.61E-35	Mast cells	ARMCX3	0.314
1.87E-38	0.31542	0.951	0.688	3.74E-35	Mast cells	BCL2L1	0.263
3.24E-38	0.273641	0.975	0.717	6.49E-35	Mast cells	CDK4	0.258
5.54E-38	0.89504	0.951	0.724	1.11E-34	Mast cells	ALOX5AP	0.227
8.26E-38	0.940644	0.877	0.713	1.65E-34	Mast cells	BACE2	0.164
5.72E-37	0.337277	0.967	0.587	1.14E-33	Mast cells	RFTN1	0.38
5.94E-37	0.780456	0.951	0.712	1.19E-33	Mast cells	NPC2	0.239
7.99E-37	0.634783	0.959	0.704	1.60E-33	Mast cells	BHLHE40	0.255
8.66E-37	0.884379	0.992	0.919	1.73E-33	Mast cells	EGR1	0.073
8.79E-37	0.957017	1	0.978	1.76E-33	Mast cells	FOS	0.022
2.65E-36	0.279125	0.984	0.778	5.30E-33	Mast cells	DNAJB9	0.206
9.04E-36	0.523552	0.934	0.617	1.81E-32	Mast cells	ELMO1	0.317
4.29E-35	0.609861	0.926	0.703	8.57E-32	Mast cells	AP1S2	0.223
5.39E-35	0.377504	0.844	0.553	1.08E-31	Mast cells	STAP1	0.291
7.94E-35	0.286784	0.967	0.655	1.59E-31	Mast cells	NUCB2	0.312
4.08E-34	0.831599	0.951	0.767	8.16E-31	Mast cells	ANXA1	0.184
5.30E-34	0.369766	0.975	0.702	1.06E-30	Mast cells	MLEC	0.273
1.28E-33	0.895723	1	0.953	2.55E-30	Mast cells	FOSB	0.047
2.03E-33	0.761584	0.91	0.654	4.07E-30	Mast cells	CD82	0.256
3.55E-33	0.747742	0.992	0.816	7.11E-30	Mast cells	ANKRD37	0.176
6.02E-33	0.708423	0.984	0.848	1.20E-29	Mast cells	TSPYL2	0.136
8.64E-33	0.451845	1	0.995	1.73E-29	Mast cells	MALAT1	0.005
8.69E-33	0.583135	0.967	0.658	1.74E-29	Mast cells	HIF1A	0.309
1.03E-32	0.283618	0.951	0.653	2.06E-29	Mast cells	HTATIP2	0.298
1.22E-32	0.37641	0.967	0.837	2.45E-29	Mast cells	KLF4	0.13
1.70E-32	0.805137	0.918	0.76	3.40E-29	Mast cells	ZC3H12A	0.158
1.74E-32	0.421843	0.828	0.618	3.48E-29	Mast cells	ST3GAL4	0.21
3.28E-32	0.519157	0.943	0.72	6.57E-29	Mast cells	PRNP	0.223
6.46E-32	0.877985	0.992	0.925	1.29E-28	Mast cells	NR4A1	0.067
1.07E-30	0.381637	0.893	0.681	2.15E-27	Mast cells	TMEM154	0.212
2.62E-30	0.302879	0.877	0.636	5.25E-27	Mast cells	TBXAS1	0.241
7.78E-30	0.774824	0.967	0.79	1.56E-26	Mast cells	HSP90B1	0.177
1.14E-29	0.396755	0.893	0.694	2.27E-26	Mast cells	MAST4	0.199
1.57E-29	0.757436	0.893	0.71	3.14E-26	Mast cells	NSMCE1	0.183
1.61E-29	0.472378	0.992	0.975	3.22E-26	Mast cells	FTL	0.017
5.31E-29	0.463153	0.852	0.639	1.06E-25	Mast cells	PAK1	0.213
5.52E-28	0.648462	0.967	0.721	1.10E-24	Mast cells	BIRC3	0.246
6.16E-28	0.64157	0.861	0.618	1.23E-24	Mast cells	TNFSF10	0.243
1.12E-27	0.669147	0.967	0.757	2.23E-24	Mast cells	EMP3	0.21
4.73E-27	0.694544	1	0.924	9.47E-24	Mast cells	SRGN	0.076
5.55E-27	0.679935	0.975	0.82	1.11E-23	Mast cells	REL	0.155
9.77E-27	0.356571	0.934	0.76	1.95E-23	Mast cells	RBKS	0.174
9.94E-27	0.575034	0.836	0.593	1.99E-23	Mast cells	CD38	0.243
2.14E-26	0.909072	1	0.945	4.27E-23	Mast cells	BTG2	0.055
9.30E-26	0.654285	1	0.985	1.86E-22	Mast cells	JUN	0.015
1.11E-25	0.410614	0.902	0.652	2.21E-22	Mast cells	PSMD1	0.25
1.45E-25	0.266481	0.926	0.698	2.89E-22	Mast cells	NFKB2	0.228
3.26E-25	0.692713	0.992	0.947	6.52E-22	Mast cells	NFKBIA	0.045
3.63E-25	0.431632	0.91	0.74	7.26E-22	Mast cells	SGK1	0.17
1.19E-24	0.408566	0.885	0.718	2.37E-21	Mast cells	DDAH2	0.167
2.27E-23	0.460009	0.836	0.646	4.54E-20	Mast cells	C1orf162	0.19
2.50E-23	0.622589	0.787	0.558	4.99E-20	Mast cells	ACSL4	0.229
3.75E-23	0.692616	1	0.947	7.50E-20	Mast cells	CD69	0.053
4.43E-23	0.294067	0.844	0.679	8.86E-20	Mast cells	ANXA4	0.165
4.79E-23	0.536091	0.836	0.587	9.59E-20	Mast cells	ZEB2	0.249
6.10E-23	0.645548	0.918	0.686	1.22E-19	Mast cells	AHNAK	0.232

1.14E-22	0.305666	0.91	0.692	2.29E-19	Mast cells	IFNGR1	0.218
1.17E-22	0.299779	0.934	0.766	2.35E-19	Mast cells	LYPLA1	0.168
1.27E-22	0.434109	0.984	0.885	2.55E-19	Mast cells	IFITM3	0.099
1.41E-22	0.498959	0.869	0.733	2.81E-19	Mast cells	AHR	0.136
2.49E-22	0.836612	1	0.877	4.98E-19	Mast cells	TNFAIP3	0.123
1.03E-21	0.332476	0.893	0.765	2.06E-18	Mast cells	STMN1	0.128
1.07E-21	0.506975	0.91	0.673	2.14E-18	Mast cells	BST2	0.237
1.50E-21	0.32027	0.926	0.738	3.00E-18	Mast cells	CANX	0.188
1.62E-21	0.361553	0.951	0.801	3.24E-18	Mast cells	PSAP	0.15
2.10E-21	0.530125	0.918	0.726	4.20E-18	Mast cells	SAMSN1	0.192
2.83E-21	0.564991	0.926	0.792	5.66E-18	Mast cells	RGS2	0.134
3.45E-21	0.350518	0.975	0.751	6.91E-18	Mast cells	CDK2AP2	0.224
4.02E-21	0.420601	0.918	0.693	8.04E-18	Mast cells	CNIH1	0.225
1.70E-20	0.29501	0.902	0.666	3.40E-17	Mast cells	IFNGR2	0.236
2.10E-20	0.406803	0.811	0.675	4.21E-17	Mast cells	FAM110A	0.136
2.36E-20	0.328298	0.91	0.752	4.73E-17	Mast cells	DDX39A	0.158
2.55E-20	0.417512	0.934	0.728	5.10E-17	Mast cells	NR3C1	0.206
5.94E-20	0.422364	0.934	0.797	1.19E-16	Mast cells	MTHFD2	0.137
1.02E-19	0.474816	0.893	0.777	2.03E-16	Mast cells	TWISTNB	0.116
1.39E-19	0.387243	0.803	0.717	2.77E-16	Mast cells	LAT2	0.086
1.56E-19	0.350849	0.844	0.552	3.11E-16	Mast cells	IGFLR1	0.292
1.76E-19	0.501429	0.844	0.728	3.52E-16	Mast cells	LAPTM4A	0.116
1.30E-18	0.478473	0.828	0.685	2.59E-15	Mast cells	TESPA1	0.143
2.25E-18	0.37422	0.828	0.712	4.50E-15	Mast cells	LAIR1	0.116
4.47E-18	0.499245	0.918	0.766	8.95E-15	Mast cells	PLIN2	0.152
2.15E-17	0.466278	0.779	0.568	4.30E-14	Mast cells	ARHGEF6	0.211
3.30E-17	0.314053	0.943	0.709	6.60E-14	Mast cells	FKBP2	0.234
5.66E-17	0.428774	0.951	0.78	1.13E-13	Mast cells	MT2A	0.171
6.59E-17	0.48222	0.738	0.584	1.32E-13	Mast cells	BTK	0.154
1.31E-16	0.329685	0.811	0.663	2.63E-13	Mast cells	MBOAT7	0.148
2.29E-16	0.506374	0.926	0.857	4.58E-13	Mast cells	HSPA5	0.069
1.26E-15	0.410162	1	0.952	2.53E-12	Mast cells	IER2	0.048
3.28E-15	0.335099	0.77	0.631	6.56E-12	Mast cells	SWAP70	0.139
3.70E-15	0.387571	0.943	0.774	7.40E-12	Mast cells	SYTL3	0.169
4.42E-15	0.362527	0.82	0.674	8.84E-12	Mast cells	BCL2A1	0.146
1.30E-14	0.322997	0.828	0.714	2.60E-11	Mast cells	SIL1	0.114
3.20E-14	0.300197	0.885	0.739	6.41E-11	Mast cells	CHST12	0.146
3.68E-14	0.360463	0.779	0.672	7.37E-11	Mast cells	ALAS1	0.107
1.10E-13	0.33176	1	0.857	2.21E-10	Mast cells	CREM	0.143
1.53E-13	0.432615	0.828	0.763	3.07E-10	Mast cells	TSC22D1	0.065
1.88E-13	0.377522	0.828	0.739	3.76E-10	Mast cells	DNAJB4	0.089
2.89E-13	0.308916	0.943	0.802	5.79E-10	Mast cells	TKT	0.141
1.29E-12	0.504227	0.992	0.892	2.58E-09	Mast cells	DUSP2	0.1
2.70E-12	0.413186	0.738	0.587	5.40E-09	Mast cells	BLVRA	0.151
1.29E-10	0.274065	0.689	0.556	2.58E-07	Mast cells	ATP8B4	0.133
2.65E-10	0.315748	0.967	0.859	5.30E-07	Mast cells	CDKN1A	0.108
1.54E-09	0.306843	0.967	0.819	3.07E-06	Mast cells	DBI	0.148
2.45E-09	0.34815	0.975	0.895	4.90E-06	Mast cells	NR4A2	0.08
2.88E-06	0.274064	0.951	0.916	0.005766	Mast cells	DDIT4	0.035
0	2.635493	0.993	0.669	0	Myeloid ce	TYROBP	0.324
0	2.174833	0.992	0.755	0	Myeloid ce	HLA-DRA	0.237
0	2.060709	0.989	0.775	0	Myeloid ce	HLA-DRB	0.214
0	1.996107	0.972	0.768	0	Myeloid ce	HLA-DPA	0.204
0	1.968425	1	0.974	0	Myeloid ce	FTL	0.026
0	1.911501	0.987	0.796	0	Myeloid ce	PSAP	0.191
0	1.904417	0.994	0.876	0	Myeloid ce	CD74	0.118

0	1.750975	0.97	0.642	0	Myeloid ce	AIF1	0.328
0	1.695424	0.955	0.581	0	Myeloid ce	IFI30	0.374
0	1.689474	0.964	0.736	0	Myeloid ce	HLA-DPB1	0.228
0	1.658015	0.96	0.705	0	Myeloid ce	NPC2	0.255
0	1.640785	0.949	0.678	0	Myeloid ce	HLA-DRB:	0.271
0	1.618813	0.954	0.658	0	Myeloid ce	FCER1G	0.296
0	1.618725	0.97	0.679	0	Myeloid ce	HLA-DQB	0.291
0	1.47173	0.945	0.696	0	Myeloid ce	GRN	0.249
0	1.303426	0.972	0.63	0	Myeloid ce	RNF130	0.342
0	1.292071	0.983	0.674	0	Myeloid ce	HLA-DMB	0.309
0	1.242159	0.938	0.746	0	Myeloid ce	FCGRT	0.192
0	1.225109	0.962	0.689	0	Myeloid ce	HLA-DMA	0.273
0	1.193854	0.998	0.93	0	Myeloid ce	SAT1	0.068
0	1.192666	0.95	0.675	0	Myeloid ce	CTSS	0.275
0	1.103897	0.999	0.991	0	Myeloid ce	FTH1	0.008
0	1.097281	0.943	0.584	0	Myeloid ce	LST1	0.359
0	1.019238	0.943	0.627	0	Myeloid ce	CTSZ	0.316
0	0.895292	0.905	0.425	0	Myeloid ce	FGL2	0.48
0	0.891351	0.939	0.637	0	Myeloid ce	C1orf162	0.302
0	0.580387	0.941	0.555	0	Myeloid ce	NUDT16	0.386
0	0.558058	0.939	0.615	0	Myeloid ce	CCRL2	0.324
0	0.53094	0.948	0.567	0	Myeloid ce	GPR34	0.381
0	0.37829	0.902	0.585	0	Myeloid ce	PLD4	0.317
6.42E-306	1.102897	0.93	0.675	1.28E-302	Myeloid ce	CTSD	0.255
1.03E-305	0.326607	0.949	0.62	2.05E-302	Myeloid ce	NFKBIE	0.329
1.99E-304	0.706817	0.922	0.628	3.99E-301	Myeloid ce	TBXAS1	0.294
6.02E-304	0.456186	0.96	0.66	1.20E-300	Myeloid ce	PRCP	0.3
1.96E-302	0.733463	0.949	0.662	3.92E-299	Myeloid ce	MFSD1	0.287
3.87E-302	1.356594	0.953	0.658	7.74E-299	Myeloid ce	LGALS1	0.295
1.49E-300	0.848186	0.913	0.58	2.99E-297	Myeloid ce	PLEK	0.333
5.30E-298	0.856756	0.892	0.61	1.06E-294	Myeloid ce	HMOX1	0.282
7.95E-297	1.167439	0.951	0.729	1.59E-293	Myeloid ce	GLUL	0.222
1.04E-296	0.552985	0.888	0.59	2.07E-293	Myeloid ce	CLEC7A	0.298
3.07E-296	0.291859	0.939	0.636	6.15E-293	Myeloid ce	FHL3	0.303
2.92E-292	0.50072	0.902	0.52	5.83E-289	Myeloid ce	C3AR1	0.382
1.15E-290	0.754971	0.902	0.625	2.30E-287	Myeloid ce	RAB31	0.277
2.56E-287	0.496167	0.956	0.706	5.13E-284	Myeloid ce	ARL5B	0.25
2.55E-281	0.576988	0.955	0.765	5.10E-278	Myeloid ce	LYN	0.19
4.78E-275	1.022486	0.937	0.671	9.56E-272	Myeloid ce	ASAH1	0.266
2.35E-270	1.10408	0.959	0.792	4.71E-267	Myeloid ce	SOD2	0.167
2.44E-269	0.450161	0.934	0.659	4.88E-266	Myeloid ce	AP1B1	0.275
7.03E-268	0.709435	0.927	0.577	1.41E-264	Myeloid ce	ZEB2	0.35
1.98E-266	0.609475	0.962	0.781	3.96E-263	Myeloid ce	MAN2B1	0.181
2.42E-258	0.320269	0.871	0.442	4.84E-255	Myeloid ce	CAMK1	0.429
4.65E-256	1.600782	0.92	0.721	9.30E-253	Myeloid ce	CTSB	0.199
1.72E-254	0.334143	0.926	0.738	3.44E-251	Myeloid ce	GRASP	0.188
1.92E-251	0.302745	0.883	0.641	3.85E-248	Myeloid ce	1-Mar	0.242
1.20E-250	0.285866	0.904	0.571	2.40E-247	Myeloid ce	RASSF2	0.333
2.05E-249	0.396361	0.931	0.655	4.09E-246	Myeloid ce	MBOAT7	0.276
2.36E-246	0.58501	0.914	0.645	4.71E-243	Myeloid ce	PGD	0.269
4.94E-245	0.918413	0.953	0.731	9.88E-242	Myeloid ce	ITGB2	0.222
1.69E-244	0.47022	0.887	0.611	3.38E-241	Myeloid ce	BASP1	0.276
3.02E-241	0.395557	0.916	0.711	6.04E-238	Myeloid ce	LAT2	0.205
4.58E-241	1.091177	0.894	0.642	9.16E-238	Myeloid ce	CCL3	0.252
6.23E-239	0.62232	0.868	0.683	1.25E-235	Myeloid ce	RNASE6	0.185
9.59E-236	0.410141	0.852	0.507	1.92E-232	Myeloid ce	CCDC88A	0.345

2.77E-235	0.339988	0.896	0.553	5.54E-232	Myeloid ce	HEXA	0.343
3.89E-235	1.314666	0.902	0.691	7.77E-232	Myeloid ce	HLA-DQA	0.211
7.26E-235	0.615785	0.966	0.734	1.45E-231	Myeloid ce	NR4A3	0.232
1.67E-233	0.398626	0.897	0.575	3.35E-230	Myeloid ce	GCA	0.322
6.19E-229	0.88106	0.894	0.605	1.24E-225	Myeloid ce	TYMP	0.289
9.78E-229	0.80956	0.92	0.713	1.96E-225	Myeloid ce	CTSH	0.207
1.90E-227	0.494165	0.875	0.705	3.80E-224	Myeloid ce	LY86	0.17
9.95E-227	0.854046	0.888	0.674	1.99E-223	Myeloid ce	LGMN	0.214
4.53E-225	0.444809	0.87	0.566	9.06E-222	Myeloid ce	ADA2	0.304
5.76E-225	0.418586	0.939	0.649	1.15E-221	Myeloid ce	ARHGAP1	0.29
1.85E-223	0.291889	0.931	0.667	3.71E-220	Myeloid ce	IRF8	0.264
6.99E-223	0.303598	0.914	0.631	1.40E-219	Myeloid ce	STX7	0.283
1.23E-221	0.269948	0.913	0.606	2.46E-218	Myeloid ce	MEF2A	0.307
6.97E-219	0.782037	0.922	0.649	1.39E-215	Myeloid ce	PPT1	0.273
1.73E-215	0.535808	0.919	0.714	3.45E-212	Myeloid ce	SLC16A3	0.205
2.57E-214	0.274127	0.914	0.592	5.14E-211	Myeloid ce	FUOM	0.322
4.19E-214	0.956371	0.948	0.791	8.38E-211	Myeloid ce	IER3	0.157
1.11E-213	0.508421	0.917	0.594	2.21E-210	Myeloid ce	TPP1	0.323
5.38E-212	0.477557	0.941	0.644	1.08E-208	Myeloid ce	AKR1B1	0.297
2.81E-208	0.745211	0.944	0.702	5.62E-205	Myeloid ce	ANXA5	0.242
9.07E-208	0.334327	0.896	0.622	1.81E-204	Myeloid ce	FUCA1	0.274
4.15E-207	0.935605	0.869	0.64	8.31E-204	Myeloid ce	CTSL	0.229
4.48E-207	0.874541	0.968	0.834	8.95E-204	Myeloid ce	CEBPD	0.134
1.36E-206	0.787129	0.926	0.753	2.73E-203	Myeloid ce	GRINA	0.173
1.93E-206	0.449472	0.914	0.697	3.86E-203	Myeloid ce	AP1S2	0.217
2.94E-206	0.673943	0.914	0.685	5.88E-203	Myeloid ce	NINJ1	0.229
8.18E-206	0.409272	0.95	0.692	1.64E-202	Myeloid ce	RASGEF1I	0.258
8.10E-204	0.375231	0.876	0.624	1.62E-200	Myeloid ce	GNS	0.252
1.24E-203	0.704142	0.971	0.84	2.49E-200	Myeloid ce	CD83	0.131
2.08E-201	0.340389	0.925	0.667	4.16E-198	Myeloid ce	FAM110A	0.258
2.69E-198	0.375696	0.865	0.641	5.38E-195	Myeloid ce	SYK	0.224
1.14E-196	0.454605	0.862	0.634	2.27E-193	Myeloid ce	THEMIS2	0.228
6.35E-195	0.687388	0.914	0.698	1.27E-191	Myeloid ce	PYCARD	0.216
2.87E-194	0.628384	0.917	0.759	5.73E-191	Myeloid ce	ETS2	0.158
8.10E-193	0.336422	0.911	0.677	1.62E-189	Myeloid ce	ABHD12	0.234
9.14E-192	0.496245	0.937	0.685	1.83E-188	Myeloid ce	IFNGR1	0.252
3.55E-188	0.683559	0.91	0.743	7.10E-185	Myeloid ce	ICAM1	0.167
1.14E-186	0.29255	0.882	0.698	2.28E-183	Myeloid ce	MEF2C	0.184
1.70E-186	0.545494	0.95	0.768	3.39E-183	Myeloid ce	COMT	0.182
3.09E-185	0.265256	0.877	0.652	6.17E-182	Myeloid ce	ENG	0.225
2.74E-182	0.319304	0.926	0.682	5.49E-179	Myeloid ce	AGTRAP	0.244
3.99E-182	0.870581	0.988	0.902	7.97E-179	Myeloid ce	S100A11	0.086
4.57E-181	0.399383	0.841	0.515	9.14E-178	Myeloid ce	SNX10	0.326
3.83E-178	0.337041	0.951	0.834	7.66E-175	Myeloid ce	KLF4	0.117
6.74E-177	0.401999	0.941	0.683	1.35E-173	Myeloid ce	CRTAP	0.258
6.81E-176	0.483458	0.863	0.684	1.36E-172	Myeloid ce	CREG1	0.179
1.21E-174	0.651357	0.96	0.746	2.42E-171	Myeloid ce	YWHAH	0.214
6.63E-174	0.444438	0.947	0.718	1.33E-170	Myeloid ce	GLRX	0.229
7.56E-174	0.673943	0.961	0.836	1.51E-170	Myeloid ce	RNASET2	0.125
2.56E-172	0.874608	0.921	0.718	5.13E-169	Myeloid ce	CD63	0.203
6.97E-172	0.375286	0.924	0.658	1.39E-168	Myeloid ce	SERPINB9	0.266
7.17E-172	0.505873	0.953	0.754	1.43E-168	Myeloid ce	RBKS	0.199
1.57E-171	0.414443	0.865	0.615	3.13E-168	Myeloid ce	LGALS9	0.25
2.60E-169	0.36186	0.953	0.736	5.21E-166	Myeloid ce	SPINT2	0.217
7.90E-169	0.677903	0.933	0.722	1.58E-165	Myeloid ce	CAPG	0.211
1.38E-168	1.112959	0.971	0.868	2.77E-165	Myeloid ce	AREG	0.103

1.25E-165	0.283665	0.93	0.749	2.51E-162	Myeloid ce	EIF4EBP1	0.181
1.39E-165	0.371787	0.947	0.683	2.79E-162	Myeloid ce	JAML	0.264
1.28E-164	1.038174	0.908	0.752	2.56E-161	Myeloid ce	TIMP1	0.156
1.15E-162	0.556509	0.857	0.661	2.31E-159	Myeloid ce	IFNGR2	0.196
1.50E-162	0.701073	0.972	0.836	3.01E-159	Myeloid ce	COTL1	0.136
3.42E-160	0.650156	0.966	0.8	6.84E-157	Myeloid ce	PKM	0.166
2.73E-158	0.348662	0.858	0.621	5.45E-155	Myeloid ce	SCPEP1	0.237
5.99E-156	0.36923	0.921	0.717	1.20E-152	Myeloid ce	SNX2	0.204
6.20E-156	0.521347	0.921	0.706	1.24E-152	Myeloid ce	MGAT1	0.215
2.63E-153	0.652611	0.956	0.831	5.27E-150	Myeloid ce	LITAF	0.125
2.98E-152	0.873641	0.848	0.669	5.95E-149	Myeloid ce	BCL2A1	0.179
2.13E-151	0.36351	0.903	0.766	4.27E-148	Myeloid ce	BRI3	0.137
1.44E-150	0.369501	0.9	0.663	2.87E-147	Myeloid ce	NCOA4	0.237
2.42E-148	0.735266	0.919	0.782	4.84E-145	Myeloid ce	NAMPT	0.137
3.68E-147	0.386741	0.914	0.668	7.35E-144	Myeloid ce	CD4	0.246
5.93E-147	0.299171	0.917	0.748	1.19E-143	Myeloid ce	GLA	0.169
8.50E-147	0.321936	0.809	0.51	1.70E-143	Myeloid ce	SKAP2	0.299
1.00E-146	0.70818	0.93	0.761	2.00E-143	Myeloid ce	PLIN2	0.169
4.16E-146	0.405423	0.801	0.592	8.32E-143	Myeloid ce	ACSL1	0.209
1.87E-144	0.57418	0.904	0.735	3.74E-141	Myeloid ce	SGK1	0.169
1.07E-143	0.571188	0.9	0.671	2.14E-140	Myeloid ce	CTSA	0.229
5.93E-143	0.430857	0.9	0.759	1.19E-139	Myeloid ce	GK	0.141
3.60E-140	0.53284	0.864	0.702	7.19E-137	Myeloid ce	PPIF	0.162
2.61E-138	0.413423	0.925	0.72	5.23E-135	Myeloid ce	CCL4	0.205
1.83E-137	0.411277	0.886	0.714	3.65E-134	Myeloid ce	PTPRE	0.172
2.15E-136	0.408771	0.941	0.702	4.30E-133	Myeloid ce	UQCRC1	0.239
1.60E-134	0.534043	0.958	0.842	3.19E-131	Myeloid ce	CEBPB	0.116
2.84E-134	0.636637	0.937	0.754	5.67E-131	Myeloid ce	ANXA2	0.183
6.59E-134	0.598236	0.932	0.792	1.32E-130	Myeloid ce	KDM6B	0.14
1.63E-132	0.485557	1	0.987	3.26E-129	Myeloid ce	ACTB	0.013
4.76E-132	0.563283	0.903	0.752	9.52E-129	Myeloid ce	AP2S1	0.151
2.13E-131	0.276985	0.897	0.655	4.26E-128	Myeloid ce	SEC14L1	0.242
1.44E-130	0.499303	0.903	0.676	2.89E-127	Myeloid ce	AKR1A1	0.227
2.85E-130	0.281565	0.955	0.757	5.70E-127	Myeloid ce	PTMS	0.198
1.77E-128	0.4678	0.837	0.601	3.54E-125	Myeloid ce	APLP2	0.236
8.70E-128	0.433102	0.835	0.651	1.74E-124	Myeloid ce	BID	0.184
8.23E-126	0.606422	0.885	0.675	1.65E-122	Myeloid ce	PLD3	0.21
7.33E-124	0.570303	0.943	0.665	1.47E-120	Myeloid ce	BST2	0.278
6.63E-122	0.475671	0.949	0.783	1.33E-118	Myeloid ce	ARPC5	0.166
1.04E-121	0.695561	0.953	0.73	2.08E-118	Myeloid ce	CTSC	0.223
1.82E-120	0.347744	0.865	0.691	3.64E-117	Myeloid ce	ATP2B1-A	0.174
3.53E-119	0.305226	0.959	0.781	7.06E-116	Myeloid ce	PER1	0.178
1.21E-118	0.518135	0.953	0.803	2.41E-115	Myeloid ce	CLTA	0.15
3.45E-118	0.5492	0.937	0.788	6.90E-115	Myeloid ce	RGS2	0.149
1.79E-115	0.502925	0.904	0.743	3.58E-112	Myeloid ce	VSIR	0.161
6.91E-115	0.282393	0.876	0.745	1.38E-111	Myeloid ce	BLVRB	0.131
4.21E-110	0.348574	0.931	0.761	8.42E-107	Myeloid ce	RILPL2	0.17
4.94E-108	0.52402	0.892	0.726	9.89E-105	Myeloid ce	TALDO1	0.166
4.05E-107	0.285198	0.816	0.623	8.11E-104	Myeloid ce	ATP6V1B2	0.193
2.95E-106	0.326337	0.899	0.735	5.89E-103	Myeloid ce	HSBP1	0.164
1.52E-102	0.429888	0.936	0.786	3.04E-99	Myeloid ce	SYNGR2	0.15
1.15E-100	0.277465	0.777	0.575	2.30E-97	Myeloid ce	DSE	0.202
2.18E-97	0.383798	0.972	0.855	4.36E-94	Myeloid ce	GSTP1	0.117
1.62E-96	0.439633	0.928	0.798	3.24E-93	Myeloid ce	TKT	0.13
3.33E-96	0.340696	0.907	0.733	6.66E-93	Myeloid ce	CANX	0.174
1.12E-95	0.352029	0.93	0.766	2.24E-92	Myeloid ce	MYDGF	0.164

1.20E-93	0.498371	0.948	0.781	2.39E-90	Myeloid ce	LSP1	0.167
9.83E-93	0.425821	0.891	0.714	1.97E-89	Myeloid ce	SERPINB1	0.177
5.77E-92	0.314704	0.916	0.759	1.15E-88	Myeloid ce	CHMP1B	0.157
1.71E-90	0.294034	0.881	0.68	3.42E-87	Myeloid ce	SAMHD1	0.201
3.16E-88	0.358406	0.927	0.781	6.32E-85	Myeloid ce	TPM4	0.146
1.55E-87	0.59111	0.853	0.704	3.10E-84	Myeloid ce	ACP5	0.149
8.56E-87	0.301785	0.778	0.581	1.71E-83	Myeloid ce	BLVRA	0.197
2.92E-84	0.276128	0.96	0.801	5.85E-81	Myeloid ce	JPT1	0.159
8.00E-83	0.418862	0.987	0.951	1.60E-79	Myeloid ce	H3F3A	0.036
4.67E-82	0.421446	0.892	0.732	9.34E-79	Myeloid ce	ATOX1	0.16
3.27E-80	0.47844	0.934	0.718	6.55E-77	Myeloid ce	ALOX5AP	0.216
5.40E-80	0.602109	0.758	0.565	1.08E-76	Myeloid ce	CCL4L2	0.193
1.02E-78	0.292624	0.945	0.834	2.04E-75	Myeloid ce	FABP5	0.111
3.22E-77	0.468737	0.994	0.922	6.44E-74	Myeloid ce	SRGN	0.072
3.23E-76	0.324223	0.956	0.815	6.45E-73	Myeloid ce	DBI	0.141
3.61E-75	0.319138	0.999	0.993	7.23E-72	Myeloid ce	TMSB10	0.006
8.59E-75	0.322653	0.954	0.868	1.72E-71	Myeloid ce	RAC1	0.086
6.30E-74	0.395118	0.933	0.752	1.26E-70	Myeloid ce	EMP3	0.181
2.37E-71	0.304943	0.934	0.807	4.73E-68	Myeloid ce	EIF4A3	0.127
1.13E-65	0.349362	0.976	0.897	2.27E-62	Myeloid ce	SLC25A5	0.079
1.20E-65	0.461251	0.934	0.839	2.41E-62	Myeloid ce	CSTB	0.095
4.12E-64	0.316206	0.976	0.843	8.24E-61	Myeloid ce	ATP5MC3	0.133
1.74E-63	0.322145	0.977	0.894	3.49E-60	Myeloid ce	VIM	0.083
2.41E-57	0.395469	0.976	0.872	4.82E-54	Myeloid ce	HSPA1A	0.104
5.49E-57	0.356874	0.945	0.817	1.10E-53	Myeloid ce	REL	0.128
2.37E-56	0.476485	0.752	0.627	4.74E-53	Myeloid ce	LIPA	0.125
3.20E-55	0.266155	0.769	0.589	6.40E-52	Myeloid ce	GSTO1	0.18
6.35E-55	0.280526	0.984	0.855	1.27E-51	Myeloid ce	HMGN2	0.129
1.18E-54	0.299162	1	0.988	2.36E-51	Myeloid ce	TMSB4X	0.012
4.32E-54	0.279024	0.718	0.563	8.64E-51	Myeloid ce	TNFSF13B	0.155
5.05E-43	0.414082	0.822	0.769	1.01E-39	Myeloid ce	UPP1	0.053
3.50E-33	0.309787	0.62	0.378	7.01E-30	Myeloid ce	CALHM6	0.242
4.33E-30	0.302821	0.959	0.903	8.67E-27	Myeloid ce	TUBA1B	0.056
0	2.328513	0.995	0.758	0	NK cells	NKG7	0.237
0	2.107638	0.98	0.598	0	NK cells	KLRD1	0.382
5.07E-298	2.824422	0.975	0.723	1.01E-294	NK cells	GNLY	0.252
1.69E-272	1.596004	0.966	0.673	3.39E-269	NK cells	TYROBP	0.293
3.31E-261	1.675957	0.953	0.661	6.61E-258	NK cells	PRF1	0.292
3.77E-242	1.47914	0.944	0.69	7.53E-239	NK cells	CTSW	0.254
4.58E-233	1.462226	0.921	0.657	9.16E-230	NK cells	CLIC3	0.264
3.05E-232	1.729674	0.939	0.735	6.10E-229	NK cells	GZMB	0.204
1.56E-216	2.031765	0.975	0.869	3.13E-213	NK cells	AREG	0.106
6.43E-215	1.305068	0.96	0.698	1.29E-211	NK cells	CST7	0.262
5.55E-208	1.199604	0.897	0.663	1.11E-204	NK cells	FCER1G	0.234
5.68E-202	2.187539	0.937	0.722	1.14E-198	NK cells	CCL4	0.215
5.47E-201	0.715249	0.856	0.602	1.09E-197	NK cells	PTGDR	0.254
4.42E-195	0.694422	0.847	0.503	8.84E-192	NK cells	IGFBP2	0.344
9.14E-193	1.390989	0.991	0.89	1.83E-189	NK cells	DUSP2	0.101
1.19E-186	1.23834	0.987	0.816	2.39E-183	NK cells	CD7	0.171
3.70E-181	1.363008	0.901	0.695	7.40E-178	NK cells	SPON2	0.206
5.90E-175	0.999215	0.906	0.761	1.18E-171	NK cells	TMIGD2	0.145
1.38E-169	0.897969	0.993	0.923	2.77E-166	NK cells	SRGN	0.07
1.20E-166	1.745118	0.858	0.645	2.40E-163	NK cells	CCL3	0.213
4.65E-164	0.954737	0.876	0.674	9.30E-161	NK cells	TXK	0.202
3.44E-163	1.016109	0.897	0.715	6.88E-160	NK cells	CMC1	0.182
4.90E-155	0.932932	0.944	0.833	9.81E-152	NK cells	LITAF	0.111

6.39E-149	0.91	0.973	0.852	1.28E-145	NK cells	SOCS1	0.121
1.11E-145	1.003679	0.939	0.779	2.22E-142	NK cells	CCL5	0.16
1.73E-142	0.794184	0.951	0.836	3.46E-139	NK cells	CEBPD	0.115
8.20E-140	0.959989	0.991	0.967	1.64E-136	NK cells	ZFP36	0.024
8.76E-137	0.908656	0.858	0.652	1.75E-133	NK cells	HOPX	0.206
9.36E-137	0.44539	0.825	0.681	1.87E-133	NK cells	GFOD1	0.144
1.12E-132	0.791747	0.923	0.772	2.25E-129	NK cells	SYTL3	0.151
1.25E-131	0.852866	0.917	0.795	2.50E-128	NK cells	CD247	0.122
9.26E-130	0.726499	0.973	0.903	1.85E-126	NK cells	IFITM1	0.07
1.69E-128	0.400971	0.85	0.742	3.38E-125	NK cells	GRASP	0.108
2.40E-122	0.295476	0.825	0.671	4.79E-119	NK cells	TLE1	0.154
3.81E-114	0.794281	0.95	0.809	7.62E-111	NK cells	KLRB1	0.141
7.46E-113	0.609191	0.795	0.647	1.49E-109	NK cells	AOAH	0.148
2.58E-111	0.518097	0.811	0.663	5.15E-108	NK cells	ADRB2	0.148
2.58E-110	0.545855	0.858	0.737	5.16E-107	NK cells	CHST12	0.121
3.67E-107	0.366234	1	0.982	7.34E-104	NK cells	MT-CO1	0.018
3.33E-105	0.870044	0.87	0.702	6.66E-102	NK cells	GZMA	0.168
3.71E-104	0.776346	0.95	0.894	7.43E-101	NK cells	NR4A2	0.056
1.16E-98	0.689486	0.98	0.952	2.32E-95	NK cells	IER2	0.028
1.45E-98	0.734685	0.993	0.961	2.90E-95	NK cells	DUSP1	0.032
1.60E-95	0.604738	0.868	0.758	3.20E-92	NK cells	RBKS	0.11
1.38E-91	0.498244	0.807	0.653	2.76E-88	NK cells	PLAC8	0.154
1.76E-91	0.755198	0.96	0.902	3.53E-88	NK cells	GADD45B	0.058
7.38E-90	0.66672	0.814	0.662	1.48E-86	NK cells	IL2RB	0.152
4.30E-86	0.596477	0.85	0.735	8.60E-83	NK cells	GPR65	0.115
1.86E-81	0.346094	0.786	0.69	3.72E-78	NK cells	RIN3	0.096
5.01E-81	0.613673	0.924	0.848	1.00E-77	NK cells	PMAIP1	0.076
5.95E-81	0.37671	0.834	0.69	1.19E-77	NK cells	ZBTB16	0.144
2.01E-79	0.598618	0.991	0.946	4.01E-76	NK cells	CD69	0.045
2.07E-78	0.604494	0.995	0.978	4.14E-75	NK cells	FOS	0.017
3.75E-78	0.32181	0.822	0.71	7.50E-75	NK cells	LAIR1	0.112
1.22E-77	0.760918	0.975	0.945	2.43E-74	NK cells	BTG2	0.03
1.74E-77	0.448291	0.894	0.816	3.48E-74	NK cells	H2AFX	0.078
6.74E-77	0.575182	0.915	0.803	1.35E-73	NK cells	LCP1	0.112
1.01E-75	0.493331	0.953	0.882	2.01E-72	NK cells	CITED2	0.071
2.90E-75	0.526115	0.899	0.778	5.81E-72	NK cells	CLEC2B	0.121
5.34E-75	0.582821	0.962	0.915	1.07E-71	NK cells	DDIT4	0.047
1.95E-74	0.312671	0.85	0.758	3.91E-71	NK cells	FAM53C	0.092
3.37E-72	0.499878	0.874	0.768	6.74E-69	NK cells	UPP1	0.106
5.71E-71	0.543753	0.827	0.718	1.14E-67	NK cells	EFHD2	0.109
5.50E-70	0.428095	0.753	0.651	1.10E-66	NK cells	PTGER2	0.102
6.79E-70	0.370355	0.82	0.765	1.36E-66	NK cells	PXN	0.055
3.37E-69	0.26749	0.825	0.703	6.75E-66	NK cells	SH3BP1	0.122
1.13E-66	0.533541	0.777	0.637	2.26E-63	NK cells	APOBEC3	0.14
1.31E-66	0.48753	0.84	0.739	2.63E-63	NK cells	SH2D2A	0.101
5.01E-66	0.307939	0.843	0.695	1.00E-62	NK cells	S1PR4	0.148
7.45E-66	0.392449	0.84	0.707	1.49E-62	NK cells	ABI3	0.133
3.41E-64	0.435374	0.912	0.814	6.82E-61	NK cells	ID2	0.098
6.91E-64	0.388777	0.802	0.704	1.38E-60	NK cells	GABARAI	0.098
2.16E-63	0.614264	0.955	0.901	4.32E-60	NK cells	KLF2	0.054
2.22E-63	0.383333	0.903	0.842	4.44E-60	NK cells	DDIT3	0.061
1.81E-60	0.483248	0.755	0.758	3.63E-57	NK cells	ADGRG1	-0.003
4.33E-60	0.293458	0.829	0.681	8.65E-57	NK cells	LYST	0.148
6.90E-60	0.333368	0.832	0.698	1.38E-56	NK cells	RASGEF1I	0.134
2.05E-59	0.28971	0.845	0.762	4.10E-56	NK cells	BCL3	0.083
6.72E-59	0.39786	0.712	0.589	1.34E-55	NK cells	PLEK	0.123

1.07E-58	0.632515	0.98	0.947	2.14E-55	NK cells	NFKBIA	0.033
3.19E-57	0.440445	0.885	0.758	6.39E-54	NK cells	ZC3H12A	0.127
1.91E-55	0.540243	0.865	0.722	3.83E-52	NK cells	ALOX5AP	0.143
4.75E-54	0.302591	0.741	0.665	9.50E-51	NK cells	LPCAT1	0.076
1.66E-53	0.389281	0.917	0.856	3.32E-50	NK cells	RHOB	0.061
3.98E-53	0.410181	0.759	0.695	7.95E-50	NK cells	GLIPR2	0.064
1.35E-52	0.285736	0.789	0.718	2.70E-49	NK cells	PTPRE	0.071
2.50E-52	0.338808	0.807	0.689	5.00E-49	NK cells	TNFRSF18	0.118
6.82E-52	0.332242	0.987	0.953	1.36E-48	NK cells	PFN1	0.034
1.23E-51	0.335107	0.883	0.791	2.46E-48	NK cells	RGS2	0.092
4.33E-51	0.452722	0.726	0.585	8.67E-48	NK cells	ZEB2	0.141
2.02E-48	0.384048	0.829	0.711	4.03E-45	NK cells	SLA	0.118
5.05E-45	0.315291	0.814	0.747	1.01E-41	NK cells	ICAM1	0.067
7.57E-45	0.296564	0.809	0.702	1.51E-41	NK cells	TIPARP	0.107
2.11E-44	0.434299	0.822	0.722	4.21E-41	NK cells	GNG2	0.1
6.75E-44	0.386464	0.798	0.759	1.35E-40	NK cells	MAP3K8	0.039
1.31E-43	0.548268	0.863	0.766	2.63E-40	NK cells	ANXA1	0.097
1.50E-43	0.371437	0.975	0.953	3.00E-40	NK cells	FOSB	0.022
4.19E-43	0.36128	0.787	0.704	8.37E-40	NK cells	BHLHE40	0.083
2.67E-42	0.305244	0.82	0.711	5.35E-39	NK cells	PIM1	0.109
4.61E-42	0.531264	0.912	0.882	9.22E-39	NK cells	RGCC	0.03
6.41E-41	0.37273	0.759	0.704	1.28E-37	NK cells	LYAR	0.055
9.77E-41	0.266173	0.856	0.766	1.95E-37	NK cells	GIMAP7	0.09
9.22E-40	0.356396	0.823	0.736	1.84E-36	NK cells	ITGB2	0.087
4.46E-39	0.317779	0.807	0.697	8.91E-36	NK cells	ARL4C	0.11
2.27E-38	0.314679	0.818	0.754	4.54E-35	NK cells	PTP4A1	0.064
2.44E-37	0.296879	0.942	0.887	4.87E-34	NK cells	TAGAP	0.055
3.71E-37	0.374685	0.996	0.982	7.42E-34	NK cells	JUNB	0.014
1.23E-36	0.332035	0.721	0.718	2.47E-33	NK cells	LAT2	0.003
1.26E-36	0.301561	0.712	0.636	2.51E-33	NK cells	RNF125	0.076
1.11E-35	0.354362	0.924	0.866	2.21E-32	NK cells	LDHA	0.058
3.45E-35	0.287587	0.854	0.733	6.91E-32	NK cells	GLIPR1	0.121
8.36E-35	0.279266	0.773	0.681	1.67E-31	NK cells	DOK2	0.092
1.85E-34	0.466509	0.894	0.778	3.71E-31	NK cells	MT2A	0.116
2.95E-34	0.488029	0.915	0.841	5.89E-31	NK cells	NFKBIZ	0.074
3.01E-34	0.294169	0.95	0.925	6.01E-31	NK cells	NR4A1	0.025
4.50E-34	0.329432	0.76	0.72	8.99E-31	NK cells	SLC16A3	0.04
1.76E-33	0.32021	0.874	0.806	3.53E-30	NK cells	XBP1	0.068
6.24E-33	0.283224	0.764	0.736	1.25E-29	NK cells	RUNX3	0.028
3.08E-31	0.344439	0.912	0.878	6.17E-28	NK cells	ATF3	0.034
9.30E-31	0.294323	0.852	0.837	1.86E-27	NK cells	NFKB1	0.015
9.23E-27	0.583777	0.645	0.57	1.85E-23	NK cells	CCL4L2	0.075
5.33E-26	0.268278	0.742	0.692	1.07E-22	NK cells	IFNGR1	0.05
3.51E-25	0.315316	0.67	0.604	7.01E-22	NK cells	TPST2	0.066
1.37E-24	0.271255	0.831	0.778	2.74E-21	NK cells	NEU1	0.053
2.22E-23	0.263659	0.98	0.956	4.44E-20	NK cells	KLF6	0.024
1.51E-22	0.39598	0.946	0.919	3.03E-19	NK cells	EGR1	0.027
1.32E-17	0.266828	0.946	0.874	2.64E-14	NK cells	HSPA1A	0.072
1.75E-12	0.32228	0.989	0.985	3.49E-09	NK cells	JUN	0.004
0	1.843763	0.956	0.755	0	T cells	IL7R	0.201
0	1.692324	0.939	0.763	0	T cells	CD3D	0.176
0	1.504735	0.826	0.709	0	T cells	CCL5	0.117
0	1.413319	0.963	0.808	0	T cells	IL32	0.155
0	1.282829	0.958	0.743	0	T cells	TNFAIP3	0.215
0	1.16029	0.852	0.746	0	T cells	KLRB1	0.106
0	1.116741	0.906	0.774	0	T cells	S100A4	0.132

0	1.067105	0.891	0.702	0	T cells	CD7	0.189
0	1.04126	0.816	0.763	0	T cells	BATF	0.053
0	0.996307	0.779	0.583	0	T cells	GZMA	0.196
0	0.951372	0.93	0.804	0	T cells	RGCC	0.126
0	0.914015	0.844	0.711	0	T cells	SPOCK2	0.133
0	0.908658	0.982	0.829	0	T cells	SRGN	0.153
0	0.85693	0.876	0.752	0	T cells	RGS1	0.124
0	0.817469	0.847	0.735	0	T cells	TRBC2	0.112
0	0.790858	0.816	0.692	0	T cells	RORA	0.124
0	0.777678	0.99	0.937	0	T cells	DNAJB1	0.053
0	0.773213	0.838	0.65	0	T cells	ANXA1	0.188
0	0.771848	0.912	0.766	0	T cells	CREM	0.146
0	0.770515	0.843	0.657	0	T cells	LAT	0.186
0	0.759128	0.832	0.663	0	T cells	GIMAP7	0.169
0	0.699581	0.841	0.726	0	T cells	CD247	0.115
0	0.68482	0.978	0.897	0	T cells	NFKBIA	0.081
0	0.684003	0.85	0.632	0	T cells	AQP3	0.218
0	0.683002	0.933	0.801	0	T cells	CITED2	0.132
0	0.673505	0.947	0.859	0	T cells	CORO1A	0.088
0	0.667473	0.756	0.565	0	T cells	ITM2A	0.191
0	0.661322	0.811	0.715	0	T cells	SYTL3	0.096
0	0.642573	0.926	0.826	0	T cells	TAGAP	0.1
0	0.633949	0.728	0.664	0	T cells	CST7	0.064
0	0.613463	0.746	0.667	0	T cells	DUSP4	0.079
0	0.605766	0.771	0.693	0	T cells	SH2D2A	0.078
0	0.60437	0.948	0.799	0	T cells	DUSP2	0.149
0	0.600451	0.729	0.564	0	T cells	TNFRSF4	0.165
0	0.594523	0.735	0.637	0	T cells	ITK	0.098
0	0.593671	0.84	0.712	0	T cells	CD27	0.128
0	0.591493	0.984	0.911	0	T cells	KLF6	0.073
0	0.580975	0.803	0.669	0	T cells	SCML4	0.134
0	0.568496	0.814	0.582	0	T cells	SAMSN1	0.232
0	0.56475	0.775	0.655	0	T cells	TRAT1	0.12
0	0.555345	0.768	0.697	0	T cells	GIMAP4	0.071
0	0.549118	0.987	0.935	0	T cells	ZFP36	0.052
0	0.540688	0.908	0.766	0	T cells	SOCS1	0.142
0	0.535264	0.978	0.895	0	T cells	CD69	0.083
0	0.531173	0.897	0.773	0	T cells	TUBA4A	0.124
0	0.529931	0.977	0.916	0	T cells	PFN1	0.061
0	0.529697	0.832	0.672	0	T cells	PTGER4	0.16
0	0.527766	0.749	0.655	0	T cells	SLA	0.094
0	0.527515	0.71	0.543	0	T cells	TRAC	0.167
0	0.526894	0.897	0.789	0	T cells	CACYBP	0.108
0	0.521068	0.756	0.592	0	T cells	SLAMF1	0.164
0	0.520858	0.63	0.511	0	T cells	CTLA4	0.119
0	0.515418	0.783	0.73	0	T cells	PRDM1	0.053
0	0.509915	0.742	0.607	0	T cells	TNFRSF18	0.135
0	0.503974	0.936	0.776	0	T cells	HSPA1A	0.16
0	0.497235	0.75	0.595	0	T cells	FLT3LG	0.155
0	0.494905	0.786	0.621	0	T cells	GNG2	0.165
0	0.493089	0.813	0.693	0	T cells	PTPN7	0.12
0	0.492529	0.839	0.712	0	T cells	FKBP5	0.127
0	0.479907	0.917	0.783	0	T cells	LDHA	0.134
0	0.477217	0.935	0.851	0	T cells	CD52	0.084
0	0.469643	0.793	0.707	0	T cells	TNFRSF1E	0.086
0	0.462558	0.764	0.67	0	T cells	NR3C1	0.094

0	0.456863	0.998	0.968	0	T cells	ACTB	0.03
0	0.456798	0.832	0.696	0	T cells	CLEC2B	0.136
0	0.442617	0.891	0.756	0	T cells	COTL1	0.135
0	0.440009	0.792	0.641	0	T cells	GLIPR1	0.151
0	0.438479	0.945	0.838	0	T cells	IFITM1	0.107
0	0.434616	0.754	0.61	0	T cells	ARL4C	0.144
0	0.424729	0.995	0.979	0	T cells	TMSB4X	0.016
0	0.423541	0.994	0.964	0	T cells	JUNB	0.03
0	0.422994	0.959	0.792	0	T cells	VIM	0.167
0	0.416427	0.795	0.713	0	T cells	TMIGD2	0.082
0	0.413754	0.796	0.631	0	T cells	LEF1	0.165
0	0.411255	0.885	0.739	0	T cells	PIM2	0.146
0	0.410618	0.833	0.761	0	T cells	LCP1	0.072
0	0.408919	0.794	0.674	0	T cells	PASK	0.12
0	0.408145	0.988	0.936	0	T cells	ACTG1	0.052
0	0.404651	0.853	0.677	0	T cells	LSP1	0.176
0	0.40267	0.904	0.804	0	T cells	ZNF331	0.1
0	0.400605	0.67	0.558	0	T cells	LINC0187	0.112
0	0.399986	0.803	0.624	0	T cells	IL6ST	0.179
0	0.397774	0.731	0.518	0	T cells	FAS	0.213
0	0.397038	0.732	0.521	0	T cells	GBP5	0.211
0	0.384783	0.803	0.687	0	T cells	TCF7	0.116
0	0.38268	0.723	0.618	0	T cells	DOK2	0.105
0	0.381525	0.739	0.561	0	T cells	CCL20	0.178
0	0.380374	0.83	0.643	0	T cells	ARID5B	0.187
0	0.378728	0.999	0.989	0	T cells	EEF1A1	0.01
0	0.378147	0.999	0.995	0	T cells	RPLP1	0.004
0	0.376446	0.913	0.834	0	T cells	SLC2A3	0.079
0	0.375108	0.835	0.729	0	T cells	GPR183	0.106
0	0.373128	0.71	0.523	0	T cells	LAG3	0.187
0	0.370667	0.838	0.698	0	T cells	ITGAE	0.14
0	0.370556	0.795	0.666	0	T cells	CORO1B	0.129
0	0.369132	0.724	0.558	0	T cells	CARD16	0.166
0	0.36865	0.739	0.613	0	T cells	ITGA4	0.126
0	0.366745	0.734	0.652	0	T cells	SIT1	0.082
0	0.352309	0.882	0.714	0	T cells	PDE4B	0.168
0	0.350744	0.716	0.653	0	T cells	GPRIN3	0.063
0	0.350488	0.867	0.693	0	T cells	UCP2	0.174
0	0.344644	0.766	0.627	0	T cells	PIM1	0.139
0	0.337341	0.801	0.609	0	T cells	FAM107B	0.192
0	0.335705	0.738	0.574	0	T cells	CD4	0.164
0	0.334863	0.767	0.568	0	T cells	JAML	0.199
0	0.32724	0.736	0.635	0	T cells	PDE3B	0.101
0	0.326753	0.729	0.589	0	T cells	FBLN7	0.14
0	0.320313	0.712	0.588	0	T cells	IL2RB	0.124
0	0.311624	0.832	0.693	0	T cells	FKBP1A	0.139
0	0.311178	0.812	0.682	0	T cells	PGAM1	0.13
0	0.308524	0.819	0.67	0	T cells	NKG7	0.149
0	0.306775	0.77	0.638	0	T cells	SUSD3	0.132
0	0.306315	0.719	0.63	0	T cells	TESPA1	0.089
0	0.30624	0.813	0.639	0	T cells	VSIR	0.174
0	0.304082	0.741	0.596	0	T cells	SAMHD1	0.145
0	0.302938	0.732	0.621	0	T cells	FAM129A	0.111
0	0.300813	0.662	0.484	0	T cells	GBP2	0.178
0	0.296323	0.772	0.68	0	T cells	GPR65	0.092
0	0.285603	0.698	0.602	0	T cells	JAK3	0.096

0	0.269898	0.709	0.551	0	T cells	TNIK	0.158
0	0.267442	0.762	0.611	0	T cells	LYAR	0.151
0	0.266899	0.689	0.534	0	T cells	TSPAN5	0.155
0	0.266362	0.813	0.682	0	T cells	RAB9A	0.131
0	0.264118	0.919	0.783	0	T cells	ZFAND5	0.136
1.07E-303	0.267656	0.926	0.779	2.14E-300	T cells	TUBA1A	0.147
4.23E-299	0.475993	0.798	0.734	8.45E-296	T cells	PHLDA1	0.064
5.26E-299	0.344054	0.783	0.663	1.05E-295	T cells	CTSC	0.12
3.63E-298	0.348669	0.748	0.718	7.26E-295	T cells	RUNX3	0.03
1.09E-294	0.40569	0.828	0.799	2.19E-291	T cells	LTB	0.029
2.04E-290	0.321194	0.992	0.966	4.08E-287	T cells	HSP90AA1	0.026
7.05E-290	0.354389	0.801	0.704	1.41E-286	T cells	HMGB2	0.097
9.65E-286	0.314135	0.736	0.663	1.93E-282	T cells	TNF	0.073
3.66E-284	0.311278	0.782	0.687	7.33E-281	T cells	BTG3	0.095
1.82E-279	0.432732	0.708	0.644	3.64E-276	T cells	CKLF	0.064
1.29E-278	0.354306	0.93	0.84	2.57E-275	T cells	H2AFZ	0.09
3.41E-278	0.269981	0.989	0.916	6.83E-275	T cells	DUSP1	0.073
1.55E-274	0.303747	0.785	0.678	3.09E-271	T cells	LDLRAD4	0.107
3.50E-274	0.297557	0.913	0.847	6.99E-271	T cells	RAN	0.066
3.58E-273	0.380272	0.656	0.545	7.15E-270	T cells	CD8B	0.111
9.11E-273	0.311074	0.75	0.63	1.82E-269	T cells	LIMS1	0.12
6.48E-272	0.337568	0.75	0.641	1.30E-268	T cells	RBPJ	0.109
1.87E-269	0.307301	0.674	0.584	3.73E-266	T cells	GALM	0.09
7.69E-267	0.44178	0.721	0.666	1.54E-263	T cells	CCR6	0.055
1.76E-264	0.415951	0.733	0.688	3.52E-261	T cells	CYTOR	0.045
3.94E-258	0.338337	0.662	0.555	7.87E-255	T cells	PTPN22	0.107
1.46E-249	0.267293	0.772	0.723	2.92E-246	T cells	PDE4D	0.049
3.38E-246	0.34051	0.636	0.563	6.77E-243	T cells	GPR171	0.073
9.91E-242	0.390197	0.738	0.704	1.98E-238	T cells	ALOX5AP	0.034
1.62E-239	0.376484	0.782	0.635	3.23E-236	T cells	CCL4	0.147
4.30E-210	0.272637	0.817	0.751	8.59E-207	T cells	H2AFV	0.066
9.37E-188	0.276383	0.756	0.698	1.87E-184	T cells	ERN1	0.058

Table S6. A list of 29 CSC marker genes associated with DSS

	p.value	HR	Low 95%CI	High 95%CI
PHLDA2	0.0442528	1.3100187	1.0069674	1.7042745
FKBP4	0.0084394	1.5981636	1.1274683	2.2653649
LMNA	0.0025966	1.9363306	1.2595856	2.9766744
BRI3	0.0360618	1.5789595	1.0301373	2.4201756
IER5L	0.0015373	1.6100161	1.1990875	2.1617703
EPS8L2	0.0201196	1.6566603	1.0823056	2.5358118
CPNE7	0.0409451	1.2064298	1.007753	1.4442754
LENG8	0.0181466	1.4400699	1.0641249	1.9488325
DDX11	0.0111707	1.7101988	1.1298694	2.5886001
HAGHL	0.0049022	1.3904706	1.1051657	1.7494286
HOOK2	0.0313762	1.6544065	1.0460095	2.6166692
BOP1	0.0299342	1.4216611	1.0347855	1.9531782
AC087239.1	0.000221	2.2479922	1.4626036	3.4551184
CEBPB	0.0361249	1.4619397	1.0248704	2.0854027
COMTD1	0.0363979	1.3209917	1.0177829	1.7145299
STXBP2	0.0159027	1.8457118	1.1215152	3.0375443
HSPB1	0.0123111	1.3835061	1.0729779	1.7839034
AGPAT2	0.028885	1.4823833	1.0413845	2.1101336
PLCG2	0.000493	1.7455357	1.2759943	2.3878592
PLEC	0.0011933	1.6686078	1.2242377	2.274274
NCOA7	0.0208422	0.7298907	0.5588279	0.9533177
USP38	0.0499219	0.6681203	0.4464461	0.9998625
HMGCS1	0.0364108	0.7422845	0.5614714	0.9813256
CD24	0.0007552	0.7193951	0.5939496	0.8713355
GFPT1	0.017982	0.6716049	0.4829437	0.9339664
ETS2	0.0007904	0.5840588	0.4266462	0.7995493
NUDT4	0.0248463	0.713533	0.531337	0.9582043
KIF9	0.0377269	0.6014288	0.3722994	0.9715746
GK	0.0208628	0.7033306	0.5218009	0.9480129
